# miR-33 deletion in hepatocytes attenuates MASLD-MASH-HCC progression

**DOI:** 10.1172/jci.insight.168476

**Published:** 2024-08-27

**Authors:** Pablo Fernández-Tussy, Magdalena P. Cardelo, Hanming Zhang, Jonathan Sun, Nathan L. Price, Nabil E. Boutagy, Leigh Goedeke, Martí Cadena-Sandoval, Chrysovalantou E. Xirouchaki, Wendy Brown, Xiaoyong Yang, Oscar Pastor-Rojo, Rebecca A. Haeusler, Anton M. Bennett, Tony Tiganis, Yajaira Suárez, Carlos Fernández-Hernando

**Affiliations:** 1 Vascular Biology and Therapeutics Program,; 2 Department of Comparative Medicine,; 3 Yale Center for Molecular and System Metabolism, and; 4 Department of Pathology, Yale University School of Medicine, New Haven, Connecticut, USA.; 5 Experimental Gerontology Section, Translational Gerontology Branch, National Institute on Aging, NIH, Baltimore, Maryland, USA.; 6 Department of Pharmacology, Yale University School of Medicine, New Haven, Connecticut, USA.; 7 Cardiovascular Research Institute and Division of Cardiology, Department of Medicine; and; 8 Diabetes, Obesity and Metabolism Institute and Division of Endocrinology, Diabetes and Bone Disease, Department of Medicine, Icahn School of Medicine at Mount Sinai, New York, New York, USA.; 9 Department of Pathology & Cell Biology and Naomi Berrie Diabetes Center, Columbia University, New York, New York, USA.; 10 Monash Biomedicine Discovery Institute, Monash University, Clayton, Victoria, Australia.; 11 Department of Surgery, Alfred Hospital and Monash University, Melbourne, Victoria, Australia.; 12 Department of Biochemistry and Molecular Biology, Monash University, Clayton, Victoria, Australia.; 13 Department of Molecular and Cellular Physiology, Yale University School of Medicine, New Haven, Connecticut, USA.; 14 Servicio de Bioquímica Clínica, Hospital Universitario Ramón y Cajal IRYCIS, Madrid, Spain.; 15 Departamento de Biología de Sistemas, Universidad de Alcalá de Henares, Madrid, Spain.

**Keywords:** Hepatology, Metabolism, Fatty acid oxidation, Liver cancer, Noncoding RNAs

## Abstract

The complexity of the mechanisms underlying metabolic dysfunction–associated steatotic liver disease (MASLD) progression remains a significant challenge for the development of effective therapeutics. miRNAs have shown great promise as regulators of biological processes and as therapeutic targets for complex diseases. Here, we study the role of hepatic miR-33, an important regulator of lipid metabolism, during the progression of MASLD and the development of hepatocellular carcinoma (HCC). We report that miR-33 was elevated in the livers of humans and mice with MASLD and that its deletion in hepatocytes (miR-33 *HKO*) improved multiple aspects of the disease, including steatosis and inflammation, limiting the progression to metabolic dysfunction–associated steatotic hepatitis (MASH), fibrosis, and HCC. Mechanistically, hepatic miR-33 deletion reduced lipid synthesis and promoted mitochondrial fatty acid oxidation, reducing lipid burden. Additionally, absence of miR-33 altered the expression of several known miR-33 target genes involved in metabolism and resulted in improved mitochondrial function and reduced oxidative stress. The reduction in lipid accumulation and liver injury resulted in decreased YAP/TAZ pathway activation, which may be involved in the reduced HCC progression in *HKO* livers. Together, these results suggest suppressing hepatic miR-33 may be an effective therapeutic approach to temper the development of MASLD, MASH, and HCC in obesity.

## Abstract 2

Hepatocyte miR-33 deficieny protects against NAFLD-NASH-HCC progression by improving hepatic lipid metabolism and mitochondrial function elucidating a novel potential target againts fatty liver disease complications

## Introduction

Metabolic dysfunction–associated liver disease (MASLD) stands as the most common chronic liver disease worldwide, affecting around 25% of the global population (1–8). Ranging from metabolic dysfunction–associated steatotic liver (MASL) to metabolic dysfunction–associated steatotic hepatitis (MASH), MASLD can progress to severe fibrosis or cirrhosis, ultimately leading to end-stage liver disease or hepatocellular carcinoma (HCC) (9, 10). The rapid rise in MASLD/MASH prevalence mirrors the increase in obesity and diabetes, making MASH the fastest growing cause of HCC globally, especially in Western populations (3, 9–12). While the primary driver of hepatic steatosis is fat accumulation in the liver, the progression of MASL to MASH is influenced by a wide variety of factors, including genetics, inflammation, oxidative stress, mitochondrial malfunction, endoplasmic reticulum (ER) stress, lipotoxicity, insulin resistance, and gut dysbiosis (3, 13, 14).

Despite the global health and economic burden associated with MASLD/MASH, approved therapies are still lacking. Therefore, there is a pressing need to identify potential therapeutic options to halt the progression of the disease and its rapid growth (15, 16). Several studies have associated MASLD with multiple metabolic alterations (9, 17–19), and impaired mitochondrial function is one of the most prominent observed in MASLD. Mitochondria are integral organelles for oxidative energy production, a process that encompasses numerous pathways including fatty acid β-oxidation (FAO), tricarboxylic acid cycle, electron transport chain (ETC), and adenosine triphosphate generation. Mitochondrial dysfunction can vary depending on the stage of MASLD, but in advanced disease it frequently includes alterations in mitochondrial number, mitochondrial DNA (mtDNA), mitochondrial biogenesis, mitochondrial dynamics, and mitochondrial recycling (18, 20–26). Coordinated regulation of these processes is essential to enhance mitochondrial activity without producing detrimental effects associated with excess mitochondrially derived reactive oxygen species (ROS) formation and redox imbalance. Conversely, targeting de novo lipogenesis (DNL) has also arisen as a therapeutic option to mitigate MASLD pathogenesis (27–30).

miRNAs have shown great promise as potential therapeutic targets for the treatment of metabolic disease, due to their ability to target numerous mRNAs and pathways simultaneously (31, 32). Previous research from our group and others identified miR-33 as an intronic miRNA hosted within the sterol regulatory element-binding protein 2 (*SREBF2*) gene (33–35). miR-33 plays a pivotal role in metabolism through the regulation of mRNA transcripts involved in a wide variety of metabolic processes, including lipid and glucose metabolism (33–40). Notably, miR-33 coordinates the expression of genes associated with mitochondrial function and homeostasis (37, 41), and increased miR-33 levels in the liver (42) and serum (43) have been associated with MASLD in humans.

Here, we elucidate a role of hepatocyte miR-33 in regulating obesity-driven MASL-MASH-HCC progression. Genetic ablation of miR-33 in hepatocytes improves metabolic function in the liver, enhancing glucose tolerance and insulin sensitivity and attenuating dyslipidemia, steatosis, and MASH. These improvements contribute to long-term reductions in liver injury and the development HCC. Mechanistically, we found that hepatocyte deletion of miR-33 increases mitochondrial FAO and alters mitochondrial dynamics, correlating with increased expression of miR-33 target genes, such as carnitine palmitoyltransferase 1A (CPT1α), PPARG coactivator 1 alpha (PGC1α), and AMPKα. miR-33 regulation of AMPKα contributes to the regulation of a subset of downstream targets and pathways, which have been recently implicated in MASH progression (44–49). Additionally, attenuation of lipid and cholesterol accumulation in the liver reduces hepatic injury, protecting from MASLD-induced HCC development in the long term. Overall, this work indicates that the deletion of miR-33 in hepatocytes is sufficient to regulate several pathways altered throughout the development of MASL/MASH/HCC, impeding the progression of the disease.

## Results

### Loss of hepatic miR-33 improves glucose tolerance, insulin sensitivity, and dyslipidemia during obesity-driven MASLD.

To study the specific role of hepatic miR-33 in MASL and its progression to MASH and HCC, we employed the conditional miR-33–knockout murine model (*miR-33^loxP/loxP^*) bred with an Albumin-Cre to induce the deletion of miR-33 in hepatocytes (*HKO*) (50). WT and *HKO* littermates were then fed a choline-deficient, high-fat diet (CD-HFD) for 3, 6, and 15 months to induce simple steatosis/MASL, steatohepatitis/MASH, and HCC, respectively (Supplemental Figure 1A; supplemental material available online with this article; https://doi.org/10.1172/jci.insight.168476DS1), as previously described (51).

**Figure 1:**
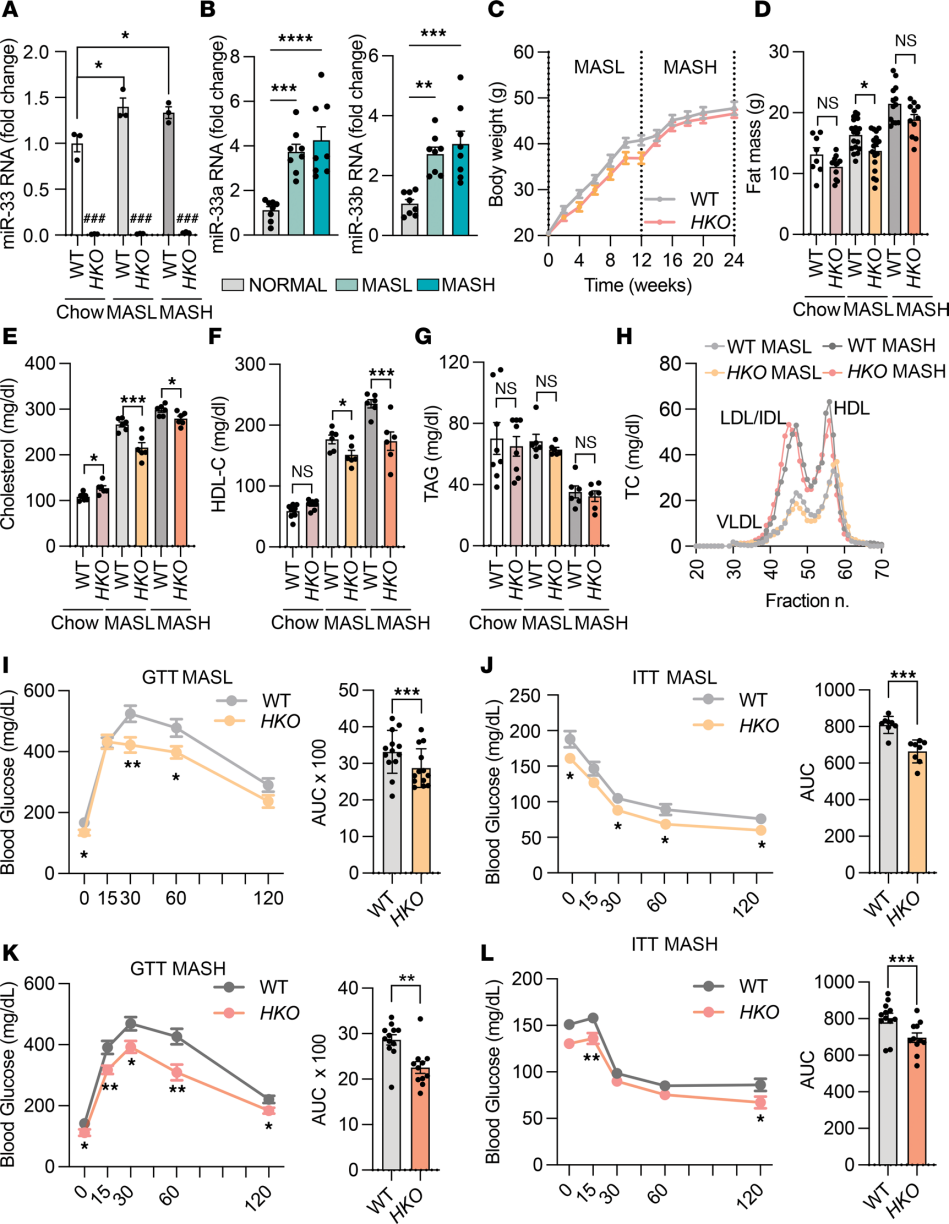
Hepatic miR-33 deficiency improves systemic metabolism in MASL/MASH. (**A**) qPCR analysis of miR-33 expression in WT and *HKO* hepatocytes fed a control diet and CD-HFD for 3 (MASL) and 6 months (MASH) (*n* = 3). (**B**) qPCR analysis of miR-33a and miR-33b expression in livers from healthy, MASL, and MASH human patients (*n* = 8). (**C** and **D**) Body weight (**C**) and body composition (**D**) analysis of WT and *HKO* mice during MASL/MASH time course (*n* = 18 WT and 16 *HKO*). (**E**–**G**) Levels of total cholesterol (**E**), HDL-C (**F**), and TAGs (**G**) in plasma of WT and *HKO* mice (*n* = 8 WT and 8 *HKO* chow; 6 WT and 6 *HKO*; MASL; 6 WT and 6 *HKO* MASH). (**H**) Cholesterol content of fast protein liquid chromatography–fractionated lipoproteins from pooled plasma of 6 WT and 6 *HKO* mice fed a CD-HFD for 3 (MASL) and 6 months (MASH). (**I**–**L**) GTT (*n* = 13, MASL; *n* = 12 WT and 11 *HKO* MASH) (**I** and **K**) and ITT (*n* = 8; *n* = 12 WT and 11 *HKO* MASH) (**J** and **L**) in WT and *HKO* mice with areas under the curve. Data represent the mean ± SEM. *P ≤ 0.05, **P ≤ 0.01, ***P ≤ 0.001, ^###^*P* < 0.001 comparing WT and HKO compared with WT animals, 2-way ANOVA followed by multiple comparison (**A**, **B**, and **D**–**G**) and unpaired 2-sided Student’s *t* test for 2-group comparisons (**I**–**L**).

To investigate the role of hepatic miR-33 in systemic metabolism and liver function during MASLD progression, we analyzed miR-33 levels after 3 and 6 months on a CD-HFD. Quantitative PCR (qPCR) analysis of freshly isolated hepatocytes confirmed miR-33 deletion in *HKO* mice, while revealing increased miR-33 levels in diet-induced MASL and MASH in control mice (Figure 1A). These findings align with recent studies showing enhanced *SREBP2* (the host gene of miR-33a) transcriptional activation in humans and other mouse models of MASL (52). We further verified this observation by measuring *SREBP2* and *SREBP1* and miR-33a/b levels in core liver biopsies from obese nonsteatotic (BMI 36–61, MAFLD activity score [MAS] = 0), obese steatotic (BMI 36–61, MAS = 1–2), and obese MASH (BMI 36–61; MAS > 5, fibrosis score = 1–2) patients. The results showed that both *SREBP1* and *SREBP2*, as well as their intronic miRNAs (miR-33b and miR-33a, respectively), were elevated in obese steatosis and obese MASH individuals compared with obese healthy individuals (Supplemental Figure 1 and Figure 1B). In agreement with the coordinated expression of SREBPs and miR-33a/b isoforms, a positive correlation was observed in their expression levels in the livers of those patients (Supplemental Figure 1C). As fatty liver and CD-HFD–induced MASLD models have been associated with metabolic dysfunctions such as obesity, dyslipidemia, and insulin resistance, we first sought to determine whether miR-33 deficiency in hepatocytes influenced obesity-driven MASLD progression (51). To this end, we analyzed the development of obesity, dyslipidemia, and insulin resistance in WT and *HKO* mice after 3 and 6 months on a CD-HFD. While no changes in body weight were observed (Figure 1C), the slight decrease in body fat accumulation in *HKO* mice (Figure 1D) was gradually attenuated over time, indicating no relevant changes in body weight or fat accumulation in our model. Circulating lipids, including total cholesterol and HDL-cholesterol, were also reduced in *HKO* mice in MASL and MASH while no changes were observed in circulating triglycerides (TAGs) (Figure 1, E–H). Finally, we assessed the regulation of glucose homeostasis and insulin sensitivity in WT and *HKO* mice by glucose tolerance test and insulin tolerance test (GTT and ITT). We found that *HKO* mice showed improved glucose metabolism after both 3 and 6 months on a CD-HFD (Figure 1, I–L). These results are consistent with our previous study showing improved systemic metabolism in *HKO* mice and reinforces the metabolic benefit of depleting miR-33 in hepatocytes, independent of the underlying dietary factors driving fatty liver progression (50).

### Genetic ablation of miR-33 in hepatocytes reduces liver steatosis by enhancing FAO and decreasing fatty acid synthesis.

**Figure 2:**
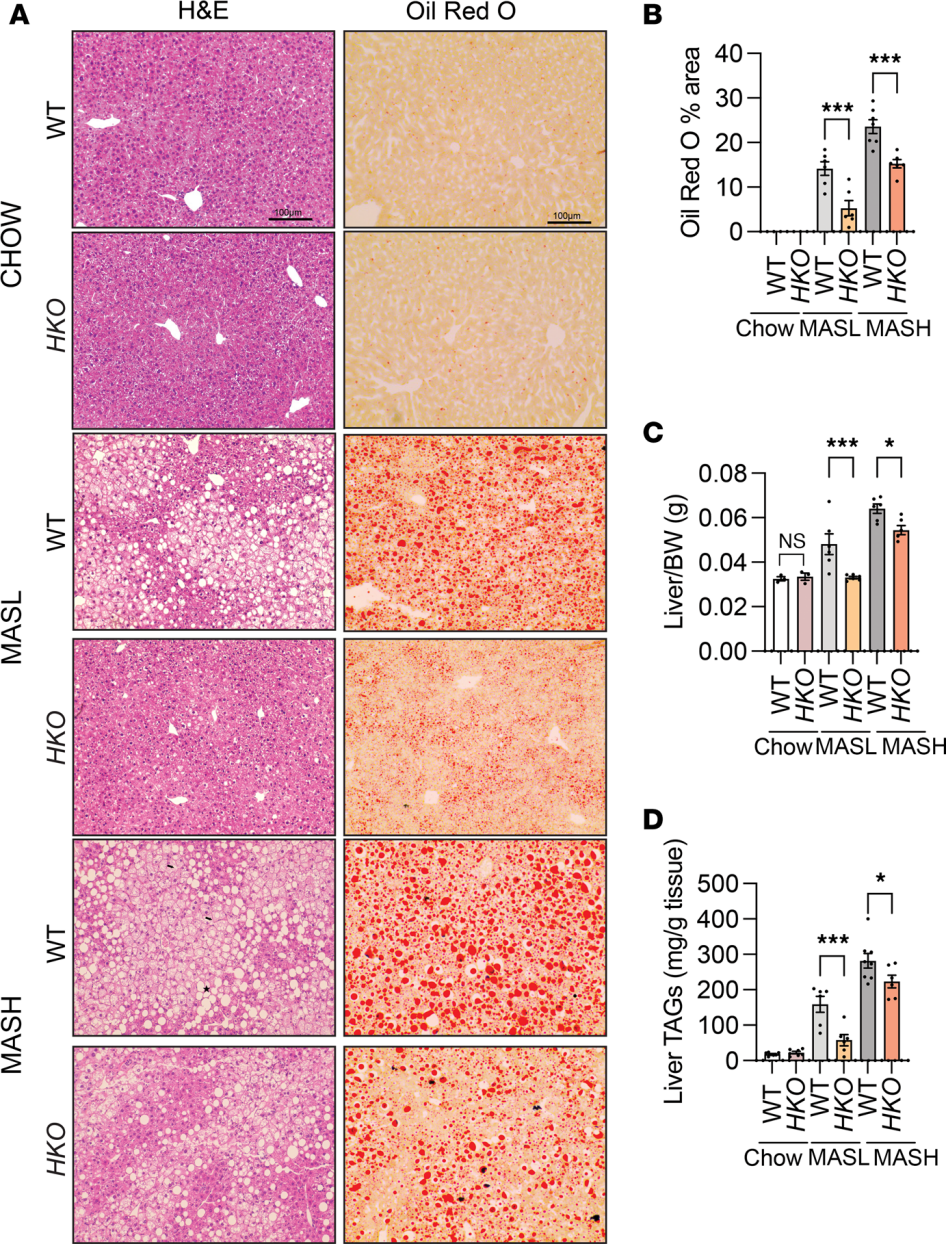
miR-33 deficiency in hepatocytes reduces liver steatosis through regulation of FA synthesis and FAO. (**A**) Representative images of H&E- and Oil Red O–stained livers from WT and *HKO* mice and (**B**) quantification of ORO staining (*n* = 3 WT and 3 *HKO* chow; 6 WT and 6 *HKO*; MASL; 7 WT and 6 *HKO* MASH). Liver weight (*n* = 3 WT and 3 *HKO* chow; 6 WT and 6 *HKO*; MASL; 7 WT and 6 *HKO* MASH) (**C**) and liver TAG (*n* = 7 WT and 7 *HKO* chow; 6 WT and 6 *HKO* MASL; 7 WT and 6 *HKO* MASH (**D**) in WT and *HKO* mice fed with a chow diet or CD-HFD. Data represent the mean ± SEM. *P ≤ 0.05, ***P ≤ 0.001 compared with WT animals, 2-way ANOVA followed by multiple comparison. FA, fatty acid.

**Figure 3:**
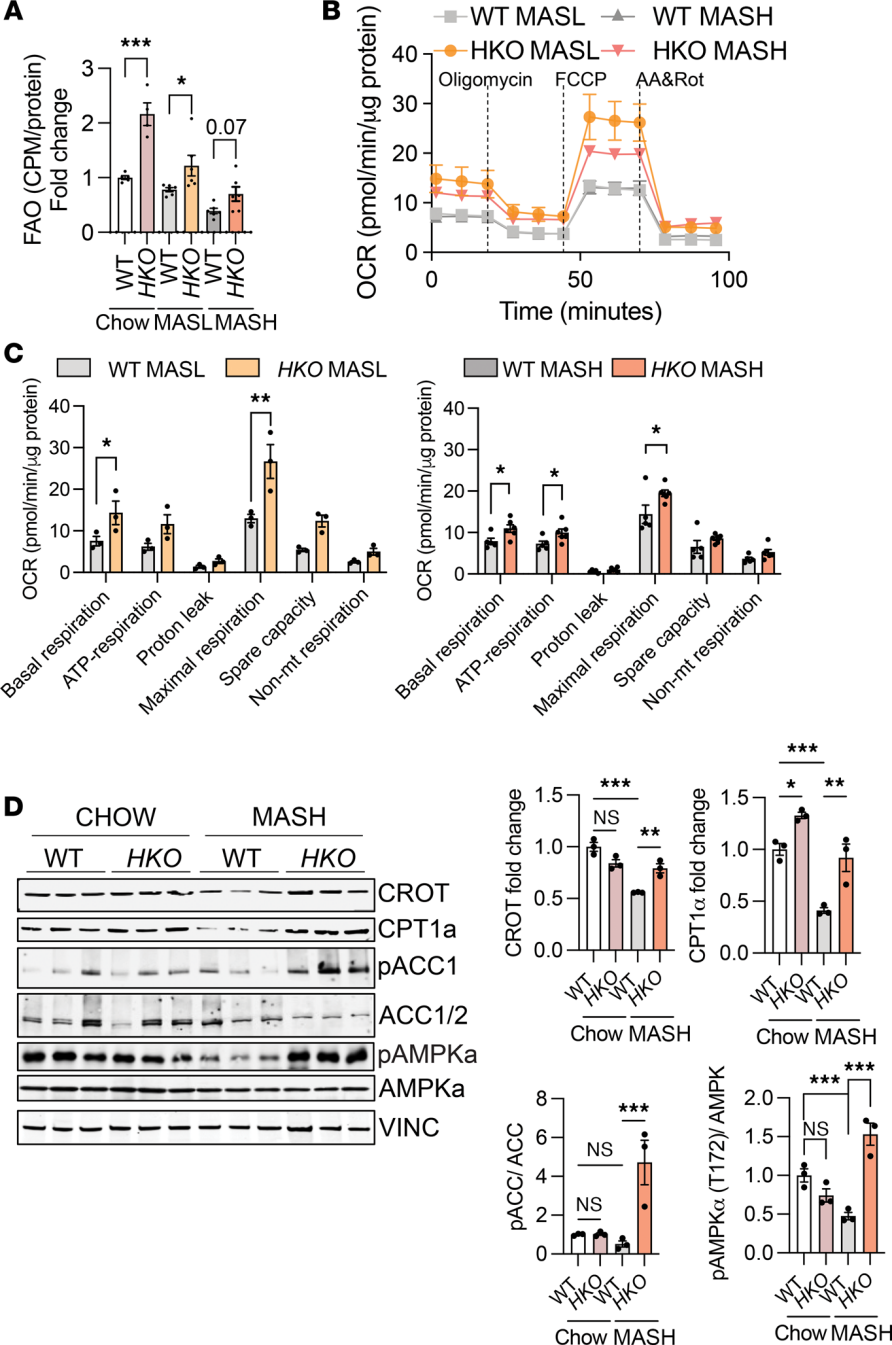
miR-33 deficiency in hepatocytes increases FAO synthesis and decreases FA synthesis. (**A**) Ex vivo analysis of FAO in WT and *HKO* livers (*n* = 5 WT and 4 *HKO* chow; 6 WT and 6 *HKO*; MASL; 6 WT and 6 *HKO* MASH). (**B** and **C**) Mitochondrial respiratory analysis inferred from oxygen consumption rate measurements of primary mouse hepatocytes isolated from WT and miR-33 *HKO* livers (*n* = 4 WT and 3 *HKO* MASL; 5 WT and 5 *HKO* MASH). (**D**) Western blot and densitometric analysis of CROT, CPT1α, phosphorylated acetyl-CoA carboxylase (p-ACC) (Ser79), total ACC, p-AMPKα (T172), total AMPKα, and housekeeping standard VINCULIN in WT and *HKO* livers. Data represent the mean ± SEM. *P ≤ 0.05, **P ≤ 0.01, ***P ≤ 0.001 compared with WT animals, unpaired 2-sided Student’s *t* test for 2-group (**B**) comparisons and 2-way ANOVA followed by multiple comparison (**A** and **D**). CPM, counts per million.

Excess hepatic lipid accumulation results from the dysregulation of one or more pathways leading to an imbalance among lipid uptake, synthesis, and oxidation (9). Our results showed a reduction in steatosis after feeding mice a CD-HFD for 3 and 6 months, which was verified by histological analysis, including H&E and Oil Red O staining (Figure 2, A and B). Additionally, liver/body weight ratio and TAG content in the livers were also reduced in *HKO* mice (Figure 2, C and D). miR-33 is an important posttranscriptional regulator of numerous genes that participate in FAO (36, 53); thus, we first sought to determine if the regulation of FAO was occurring in our model of MASLD. Ex vivo analysis of the rate of [^14^C]-palmitate oxidation showed increased liver FAO in *HKO* mice (Figure 3A). We further characterized the contribution of miR-33 to mitochondrial metabolism by measuring the respiratory capacity of freshly isolated hepatocytes from CD-HFD fed WT and *HKO* mice, verifying the increase in mitochondrial respiration in hepatocytes lacking miR-33 (Figure 3, B and C). Mechanistically, we observed that carnitine O-octanoyltransferase (CROT) and the mitochondrial FA transporter, CPT1α, both bona fide targets of miR-33 and key molecules that participate in FAO, were significantly upregulated in *HKO* livers (Figure 3D and Supplemental Figure 2D).

Next, we aimed to determine whether hepatocyte miR-33 deficiency influenced DNL during MASLD progression. To this end, we assessed the activities of fatty acid synthase (FASN) (the enzyme involved in the synthesis of FAs from acetyl-CoA and malonyl-CoA) and 3-hydroxy-3-methylglutaryl–CoA reductase (HMGCR) (the rate-limiting enzyme for cholesterol synthesis) in freshly isolated liver homogenates from WT and *HKO* mice. The results showed decreased activity of both enzymes in *HKO* livers (Supplemental Figure 2, A and B). Additionally, ex vivo measurement of *DNL* was assessed in WT and *HKO* livers, verifying a decreased rate of acetate incorporation into lipids in *HKO* livers after CD-HFD feeding (Supplemental Figure 2C). Consistently, we observed that *HKO* livers had increased Ser79 phosphorylation of acetyl-CoA carboxylase (ACC) (Figure 3D and Supplemental Figure 2D). The increased hepatic FAO and suppression of DNL observed in *HKO* mice correlated with a significant increase in AMPKα activation (as assessed by phosphorylation) (Figure 3D and Supplemental Figure 2D). In contrast, the expression and phosphorylation of AMPKβ were not altered (Supplemental Figure 2D). Thus, the attenuation of MASLD progression mediated by miR-33 deletion in hepatocytes is through the regulation of multiple metabolic pathways.

**Figure 4:**
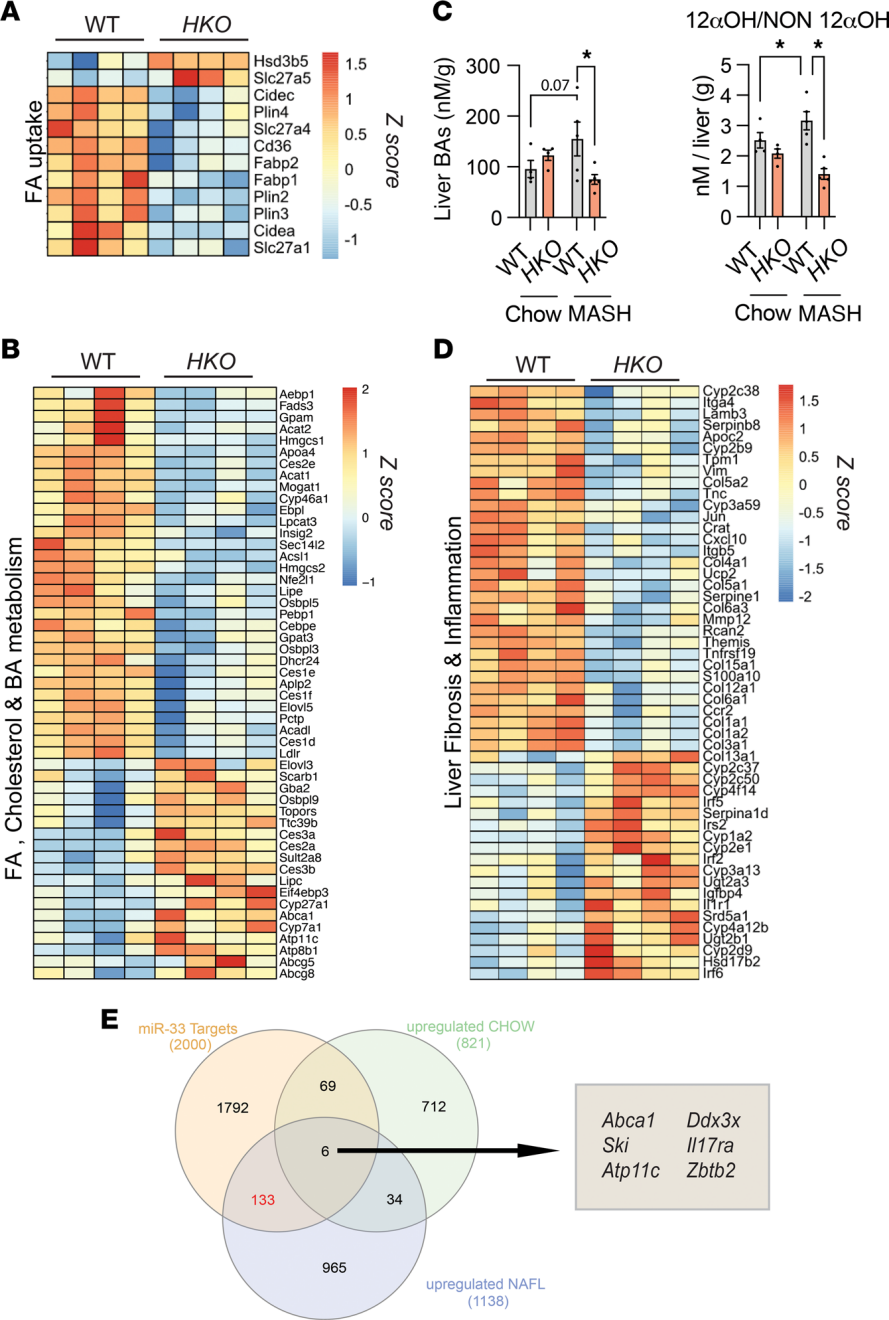
RNA-Seq in MASL livers reveals global changes in gene expression regulated by miR-33. (**A**, **B**, and **D**) Heatmaps of pathways relevant to MASLD progression in livers from WT and *HKO* mice. Cutoff values were settled as fold-change > log_2_1.5 and *Padj* < 0.05. (*n* = 4.) (**C**) Comparison of liver BAs and BA distribution of the 12α-hydroxy/non–12α-hydroxy ratio in WT and *HKO* livers. (*n* = 4–5.) (**E**) Venn diagram depicting the overlap between miR-33 predicted targets and genes upregulated in *HKO* livers versus WT livers in mice fed a chow diet and CD-HFD. Data represent the mean ± SEM (*P ≤ 0.05 compared with WT animals, unpaired 2-sided Student’s *t* test for 2-group comparisons and 2-way ANOVA followed by multiple comparison).

Given the profound metabolic alterations observed in miR-33 *HKO* livers, we next assessed global transcriptional changes by RNA-Seq analysis in the livers of WT and *HKO* MASL mice, aiming to identify specific genes or upstream regulators involved in these functions. We found 1,082 differentially expressed genes (DEGs) (421 upregulated and 661 downregulated in *HKO*, adjusted *P* [*Padj*.] < 0.05), indicating the broad effect that miR-33 deficiency has in the liver during steatosis initiation. Interestingly, genes involved in metabolic functions and pathways altered in obesity-driven MASLD revealed that gene signatures associated with FA uptake, FA synthesis, and cholesterol homeostasis were altered in *HKO* livers (Figure 4, A and B). Of interest was the upregulation of ATP binding cassette subfamily A member 1 (*Abca1*) and cytochrome P450 family 7 subfamily A member 1 (*Cyp7a1*) observed in *HKO* livers, given their participation in cholesterol and bile acid (BA) metabolism and their direct regulation by miR-33 (33, 54). Thus, we sought to determine whether hepatocyte deletion of miR-33 was playing a role in cholesterol and BA metabolism in the liver. ABCA1 and CYP7A1 upregulation in MASLD *HKO* livers was verified by Western blot analysis (Supplemental Figure 3A). Moreover, RNA-Seq analysis also revealed upregulation of other genes related to BA metabolism, including *Abcb11*, *Cyp27a1*, *Abcg8*, *Abcg5*, *Atp11c*, and *Atp8b1* (Figure 4B). Given the observed regulation of BA metabolic genes in our model, we measured BA profiles in livers from WT and *HKO* mice fed a chow diet or a CD-HFD. In mice fed a CD, differences were found only in the levels of cholic acid, with no other differences observed for other BA species or total BA content (Supplemental Figure 3B). By contrast, when mice were fed the CD-HFD, a more profound dysregulation of the hepatic BA profile was observed. Total BAs were increased in WT mice but maintained at standard basal levels in *HKO* livers (Supplemental Figure 3B). Additionally, deoxycholic acid (DCA), one of the most toxic BAs, was reduced in *HKO* livers compared with WT livers fed the CD-HFD (Supplemental Figure 3B). Analysis of different species related to liver pathologies, including 12αOH/NON-12αOH, unconjugated/conjugated, and secondary/primary ratios, revealed different trends in the relative amounts of these BAs in WT livers under CD-HFD feeding but not in *HKO* livers (Figure 4C and Supplemental Figure 3C), correlating with insulin resistance and liver injury (55–60). Finally, we further interrogated our RNA-Seq data for changes in well-known specific processes associated with MASLD progression, including inflammatory, pro-fibrogenic, and CYP450-associated functions (61, 62). We observed downregulation of genes associated with inflammation and fibrogenesis in *HKO* livers, while repression of CYP expression was prevented (Figure 4D). To gain better insight into the direct impact of miR-33 on liver gene expression signature, we sought to identify potential genes directly targeted by miR-33 in the liver in healthy and MASLD conditions. To do this, we assessed the overlap between genes upregulated in *HKO* livers from chow diet (50) and CD-HFD mice and the top 2,000 genes predicted to be miR-33 targets by TargetScan7.2. While only 6 genes, including *Abca1*, *Ski*, and *Atp11c*, were found in the intersection of all 3 conditions, more overlapping of genes (133 genes) was found in the intersection between miR-33 targets and those upregulated in *HKO* under MASLD than in CD-fed mice (Figure 4E and Supplemental Table 1). These findings highlight the potential direct role of several miR-33 targets, including genes known to regulate metabolism and liver injury, such as *Abca1*, *Ski*, *Atp11c*, *Slc25a51*, *Atp8b1*, *Atp11a*, *Slc30a1*, *Irs2*, *Acadsb*, *Serpind1*, *Klf15*, and *Edem1*, in the progression of the disease. Moreover, these results suggest that most of the observed changes in the *HKO* mice are not a consequence of basal gene regulation mediated by miR-33 deletion but are a consequence of changes that occur during the progression of the disease. Overall, our analysis suggests miR-33 *HKO* mice are protected from MASLD progression through the regulation of metabolic function at multiple levels, resulting in increased FAO and mitochondrial function and decreased DNL and cholesterol metabolism.

### miR-33 HKO mice are protected from diet-induced MASH and fibrosis.

**Figure 5:**
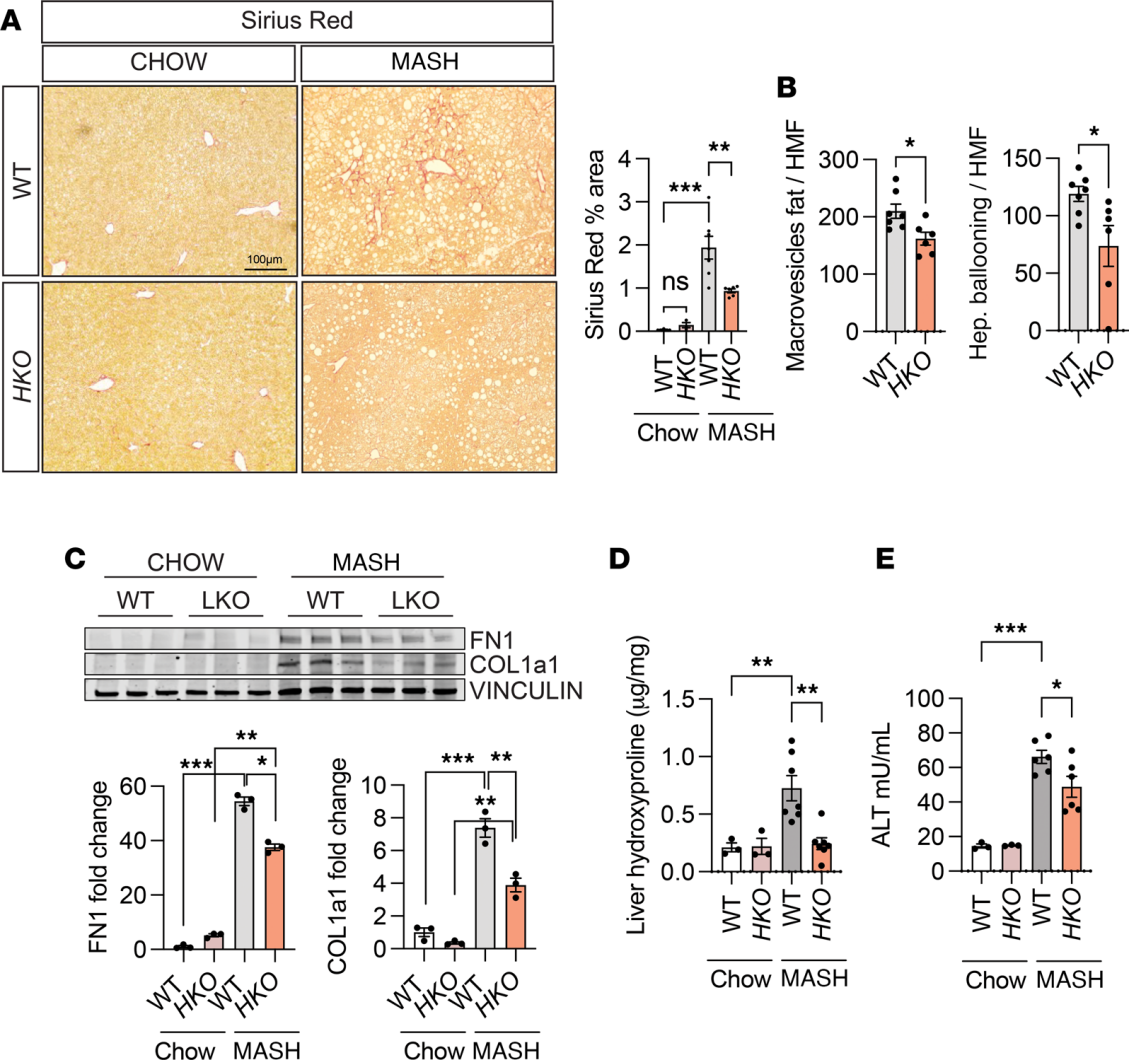
Loss of hepatic miR-33 attenuates liver fibrosis and MASH. (**A**) Representative images of Sirius red–stained livers from WT and *HKO* mice. Indicated quantification on the right (*n* = 3 chow; 7 WT and 6 *HKO* MASH). (**B**) Graphical quantification of macrovesicular fat and hepatocyte ballooning from WT and *HKO* livers normalized per high-magnification field area (HMF) (*n* = 7 WT and 6 *HKO*). (**C**) Western blot and densitometric analysis of FN1, COL1A1, and housekeeping standard VINCULIN in WT and *HKO* livers. (**D**) Hydroxyproline content in MASH WT and *HKO* livers (*n* = 3 chow; 7 MASH). (**E**) Serum ALT in WT and *HKO* mice (*n* = 3 chow; 6 MASH). Data represent the mean ± SEM (*P ≤ 0.05, **P ≤ 0.01, ***P ≤ 0.001 compared with WT animals, unpaired 2-sided Student’s *t* test for 2-group comparisons and 2-way ANOVA followed by multiple comparison).

The adverse outcomes associated with MASH and the subsequent fibrosis encompass the progression to cirrhosis and end-stage liver disease or HCC (9). Based on the improved metabolic function and the beneficial changes in the gene expression profile in *HKO* livers, we examined how miR-33 deletion specifically affects the development of liver fibrosis during MASH. Sirius red staining of liver sections revealed a strong decrease in collagen content in *HKO* livers (Figure 5A). Moreover, histopathological analysis of H&E-stained liver sections from Figure 2A revealed decreased macrovesicular fat content and hepatocyte ballooning in *HKO* livers (Figure 5B). Consistently, we observed a significant reduction in liver fibrosis markers, including fibronectin (FN1) and collagen type I alpha 1 chain (COL1A1), as well as total hydroxyproline content in *HKO* mice (Figure 5, C and D). Attenuation of liver fibrosis in the absence of hepatic miR-33 was not accompanied by significant reduction in liver inflammation (Supplemental Figure 4, A–E). Reduction in liver injury in mice lacking miR-33 in hepatocytes was also verified by reduced serum levels of alanine aminotransferase (ALT) (Figure 5E). Together, our findings suggest that miR-33 deficiency in hepatocytes protects CD-HFD–fed mice from diet-induced liver injury and progression to fibrosis.

### Loss of miR-33 regulates miR-33 target genes exclusively in hepatocytes and triggers metabolic changes and cellular cross-communication in the liver.

**Figure 6:**
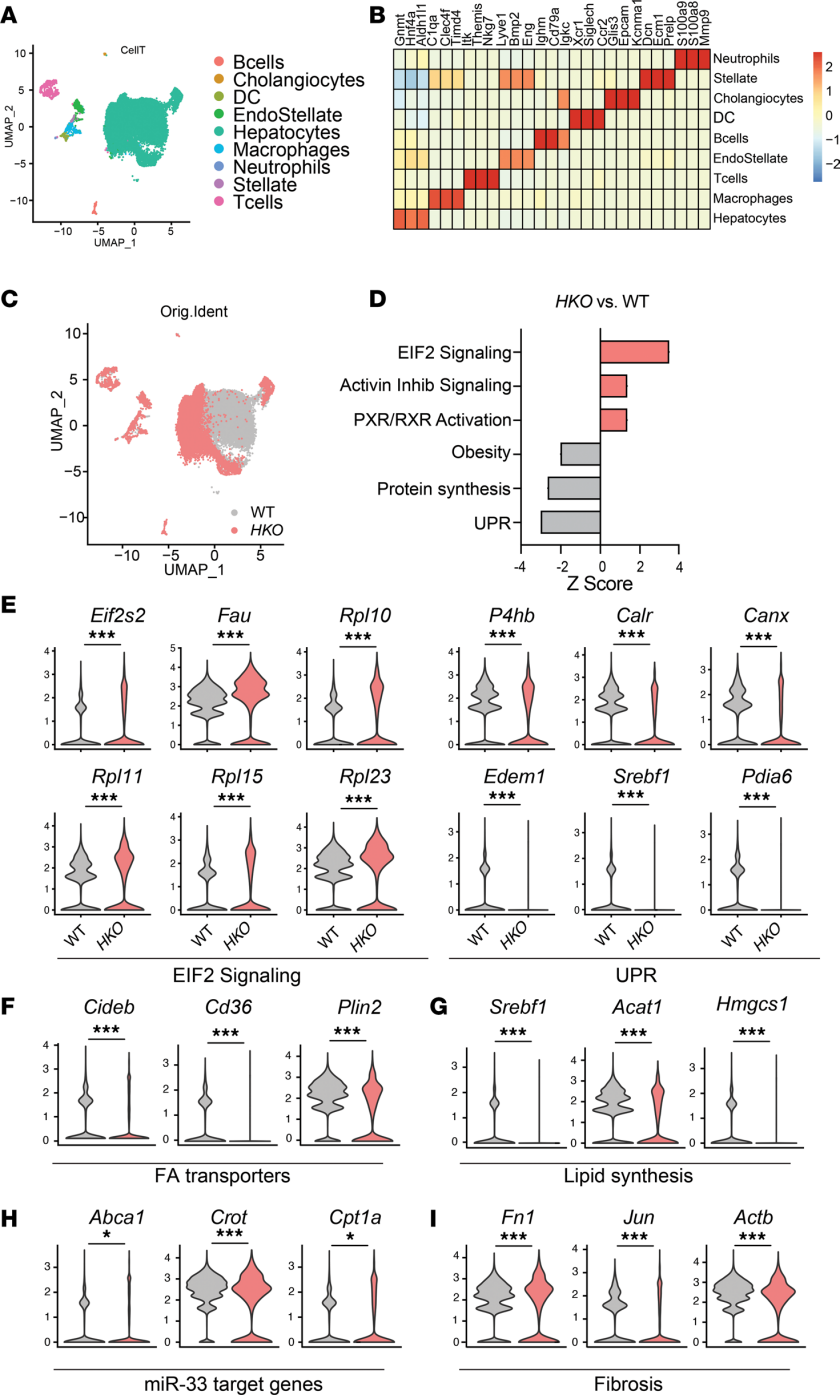
Hepatic loss of miR-33 attenuates liver injury through the crosstalk of different cell types. Uniform manifold approximation and projection (UMAP) (**A**) and heatmap (**B**) representation of cell clusters identified from scRNA-Seq analysis. (**C**) UMAP of single-cell profiles from WT (gray) and *HKO* (red) mouse hepatocytes identified from scRNA-Seq analysis. (**D**) Canonical pathways represented by *z* score among differentially expressed genes in scRNA-Seq analysis of hepatocytes from WT and *HKO* mice. Red bars indicate pathways in which genes are upregulated in *HKO*, and gray indicates downregulated pathways on the predicted *z* score. All represented pathways were significantly changed with a –log *P* > 1.5. (**E**–**I**) Violin plots representing the top upregulated and downregulated genes significantly altered in the indicated pathways in hepatocytes. Violin plot data display single-cell distribution of the indicated experimental groups. Orig.Ident, original identity.

**Figure 7:**
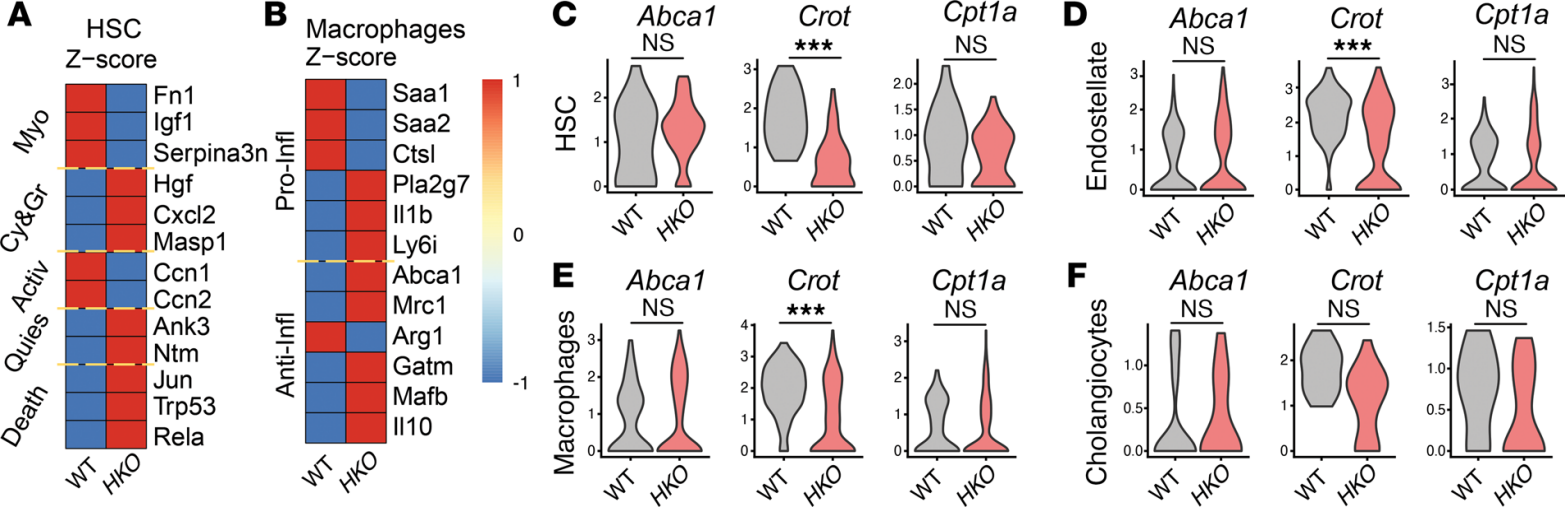
miR-33 deficiency attenuates liver fibrosis without regulating miR-33 target genes in nonhepatocyte liver cells. (**A** and **B**) Heatmap showing HSC activation (**A**) and macrophage inflammatory/noninflammatory markers (**B**) identified from scRNA-Seq from WT and *HKO* mice livers. Color codes referred to *z* score. (**C**–**F**) Violin plots showing expression changes of miR-33 target genes in nonhepatocyte cells, including HSCs (**C**), endostellate cells (**D**), macrophages (**E**), and cholangiocytes (**F**). Violin plot data display single-cell distribution of the indicated experimental groups. ***Padj < 0.001 using default statistical test (Wilcox test) from Seurat package in R studio.

In order to understand the potential regulatory effects of miR-33 regulation in the different cell populations in the liver during the advanced stages of the disease, we performed single-cell RNA-sequencing (scRNA-Seq) in WT and *HKO* livers with MASH (Figure 6, A–C). UMAP analysis of liver cells showed that most evident changes between WT and *HKO* cells were found in the population identified as hepatocytes (Figure 6C). Unbiased analysis of changes within hepatocytes identified downregulation of pathways related to obesity, protein translation, and unfolded protein response in *HKO* hepatocytes, while pathways related to EIF2 signaling, inhibition of activin, and activation of RXR were upregulated in these cells (Figure 6, D and E). Additionally, analysis of hepatocytes verified the alterations in metabolic and fibrogenic genes, as well as miR-33 bona fide target genes (Figure 6, F–I). Moreover, we analyzed different cell populations and interactions involved in liver fibrosis, aiming to understand how miR-33 deletion in hepatocytes could affect other cells. To monitor for hepatic stellate cell (HSC) activation, the genetic profile of HSCs was analyzed based on different characteristics known to contribute to their activation and liver fibrosis (63). HSCs were classified and named according to their activated phenotype and pro- or antifibrogenic function as follows: activated myofibroblast HSC (*Myo*) (pro-fibrogenic), cytokine-producing HSC (*Cy&Gr*) (antifibrogenic), classically activated (*Activ*) (pro-fibrogenic), quiescent (*Quies*) (antifibrogenic), and apoptotic HSC (*Death*) (antifibrogenic) (63). *Myo* and *Activ* markers were increased in WT HSCs, whereas markers of *Cy&Gr*, *Quies*, and *Death* of HSC were higher in HSCs derived from *HKO* livers, indicating reduced activation of HSCs in this group (Figure 7A). On the other hand, although antiinflammatory markers of macrophages showed a trend toward increased activation in *HKO* livers, analysis of inflammatory markers aligned with our previous data showing no clear regulation in *HKO* livers (Figure 7B). Finally, we analyzed different cell types from our scRNA-Seq looking for potential changes in miR-33 target genes that could suggest an existing communication between different cell types and an indirect gene regulation from hepatocyte-derived miR-33. With that purpose, bona fide targets of miR-33 (*Abca1*, *Crot*, and *Cpt1a*) were analyzed in different cell types, including HSCs, endostellate cells, macrophages, and cholangiocytes. While downregulation of some of those genes were observed in some of the groups, the clear lack of upregulation of these bona fide miR-33 target genes in the *HKO* group suggests no direct effect of miR-33 deletion in these cells when miR-33 was removed from the hepatocytes (Figure 7, C–F). We also performed cell-cell communication analysis to get an overall picture of the different interactions occurring in WT and *HKO* livers. Cell-cell communication analysis showed increased total number and strength of cell-cell communication in WT livers compared with *HKO* livers (Supplemental Figure 5, A–C), with hepatocytes being the main cell type contributing to cell communication in the liver (Supplemental Figure 5C). Cell-cell communication analysis also revealed that the most relevant differences in WT versus *HKO* livers were found in pathways related to leukocyte activation and markers of liver fibrosis mostly derived from the hepatocytes (Supplemental Figure 5, D and E). By contrast, signals upregulated in *HKO* livers were more heterogeneous, including signals related to HSC senescence and death and quiescence — chemerin (64, 65), BMPs (66–68), and CALCR (69); signals classically associated with liver fibrosis (TGF-β and PDGF); and signals linked to hepatocyte regeneration and death prevention — TWEAK (70) and NOTCH (Supplemental Figure 5, D and E). Finally, we attempted to analyze specific ligand-receptor differences in WT versus *HKO* cell-cell communication from hepatocytes to other liver cells; however, no differences in terms of signaling were found. Taken together, our results indicate that the main effect produced by miR-33 deletion in hepatocytes is specific to these cells and the effects observed in recruitment and activation of inflammatory and hepatic stellate cells are a consequence of changes produced initially in hepatocytes.

### miR-33 deficiency in hepatocytes prevents mitochondrial dysfunction associated with MASLD-MASH progression.

**Figure 8:**
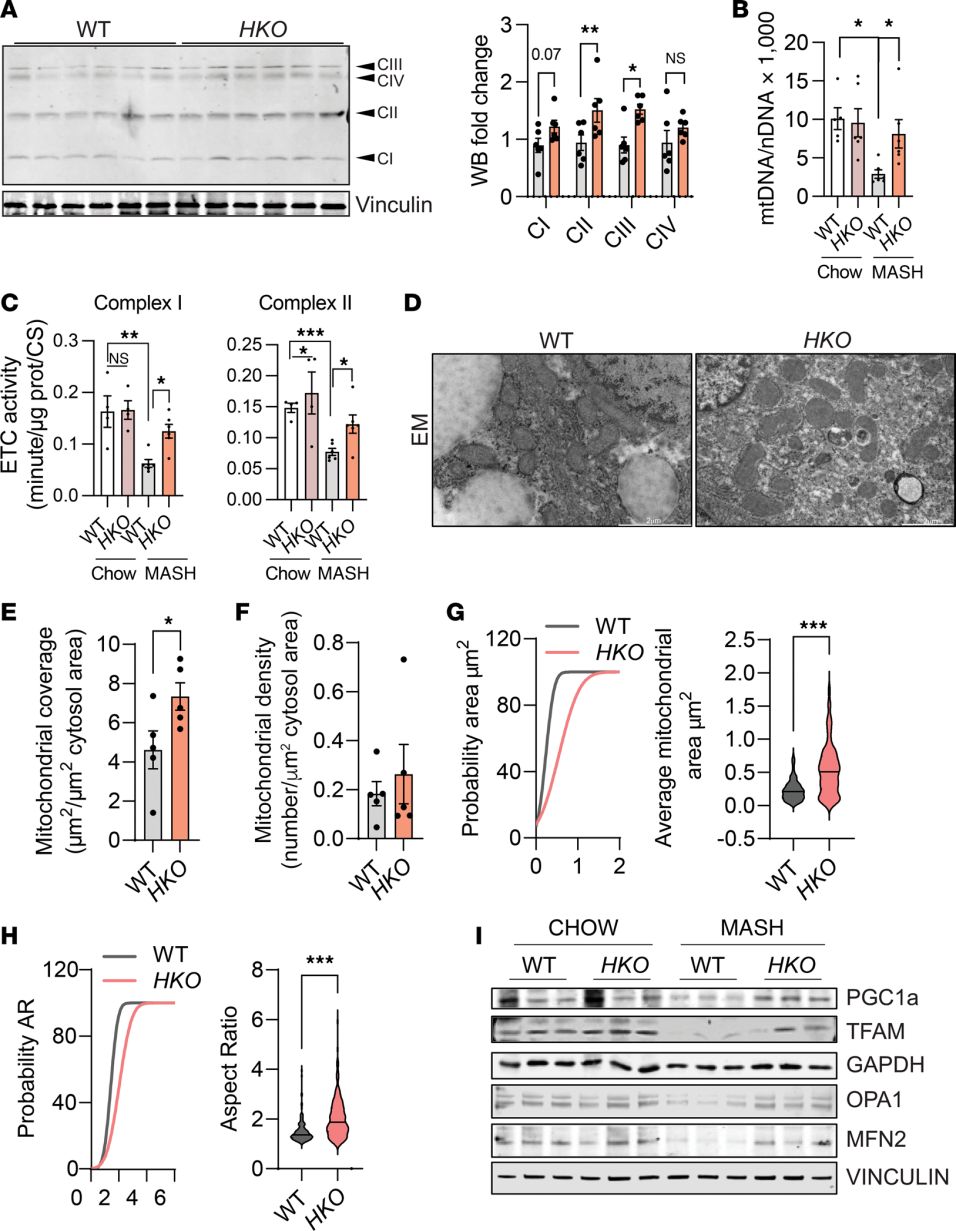
Hepatic miR-33 deficiency improves mitochondrial function and homeostasis. (**A**) Western blot and densitometric analysis of different mitochondrial subunits blotted with the Total OXPHOS Rodent WB Antibody Cocktail (Abcam ab110413) and housekeeping standard VINCULIN in WT and *HKO* livers from mice fed with CD-HFD for 6 months (*n* = 6). (**B**) qPCR analysis of mitochondrial DNA and nuclear DNA in WT and *HKO* livers. Data represented as mtDNA/nDNA (*n* = 6). (**C**) Activity of the ETC complex I and complex II in MASH livers. Enzyme activities are expressed as change in absorbance/min/μg protein/citrate synthase activity (*n* = 4–6). (**D**) Representative electron micrographs of mitochondria profiles in WT and *HKO* hepatocytes from MASH livers. (**E**–**H**) Mitochondrial coverage (**E**), mitochondrial density (**F**), cumulative distribution and mean of mitochondrial area (**G**), and mitochondria aspect ratio (**H**) from WT and *HKO* hepatocytes (*n* = 3–4). (**I**) Western blot of PGC1α, TFAM, MFN2, OPA1, and housekeeping standard VINCULIN or GAPDH in WT and *HKO* livers. Data represent the mean ± SEM. *P ≤ 0.05, **P ≤ 0.01, ***P ≤ 0.001 compared with WT animals, unpaired 2-sided Student’s *t* test for 2-group comparisons and 2-way ANOVA followed by multiple comparison (**B**, **C**, and **I**).

Mitochondrial dysfunction is a common feature underlying MASLD-MASH progression (18, 20–26). miR-33 acts as a critical regulator of mitochondrial function through the targeting of multiple factors, including PGC1α and AMPKα (40, 41, 71). Thus, we next aimed to further characterize the molecular mechanisms that mediate the improved mitochondrial function observed in miR-33–deficient hepatocytes. We found increased mitochondrial content in hepatocytes from *HKO* MASH livers, as assessed by the protein levels of different complexes of the ETC and by the mitochondrial to nuclear DNA ratio (mtDNA/nDNA) (Figure 8, A and B). These findings were further supported by electron microscopy analysis of hepatocytes from MASH mice, which revealed an increase in the coverage and density of mitochondria, as well as mitochondrial elongation in *HKO* mice (Figure 8, D–H). We also observed enhanced mitochondrial ETC activity of complex I and complex II (Figure 8C). The increase in mitochondrial mass found in hepatocytes from *HKO* mice was correlated with elevated levels of PGC1α, which is a direct target of miR-33 (72, 73). Moreover, PGC1α’s downstream target, mitochondrial transcription factor A (TFAM), was also upregulated in livers from miR-33–deficient hepatocytes (Figure 8I). Together, these results demonstrate that absence of miR-33 in hepatocytes improves mitochondrial function by increasing mitochondrial mass and ETC activity.

Mitochondrial homeostasis is critical for the control of mitochondrial health and metabolism (18, 24, 74). Mitochondrial quality control mechanisms include mitochondrial biogenesis and dynamics, a process that involves fusion and fission of mitochondrial membranes and is dysregulated in MASLD. Mitochondrial number and size are also controlled through the balance of mitochondrial dynamics (22, 24, 74, 75). Thus, we sought to characterize mitochondrial dynamics in our MASH model. We found an increase in fusion-related proteins MFN2 and OPA1 but no relevant changes in fission proteins (Figure 8I and Supplemental Figure 6A). Importantly, the increased MFN2 level is consistent with the changes in mitochondrial shape observed by electron microscopy and correlates with the increased respiratory capacity of these mice.

**Figure 9:**
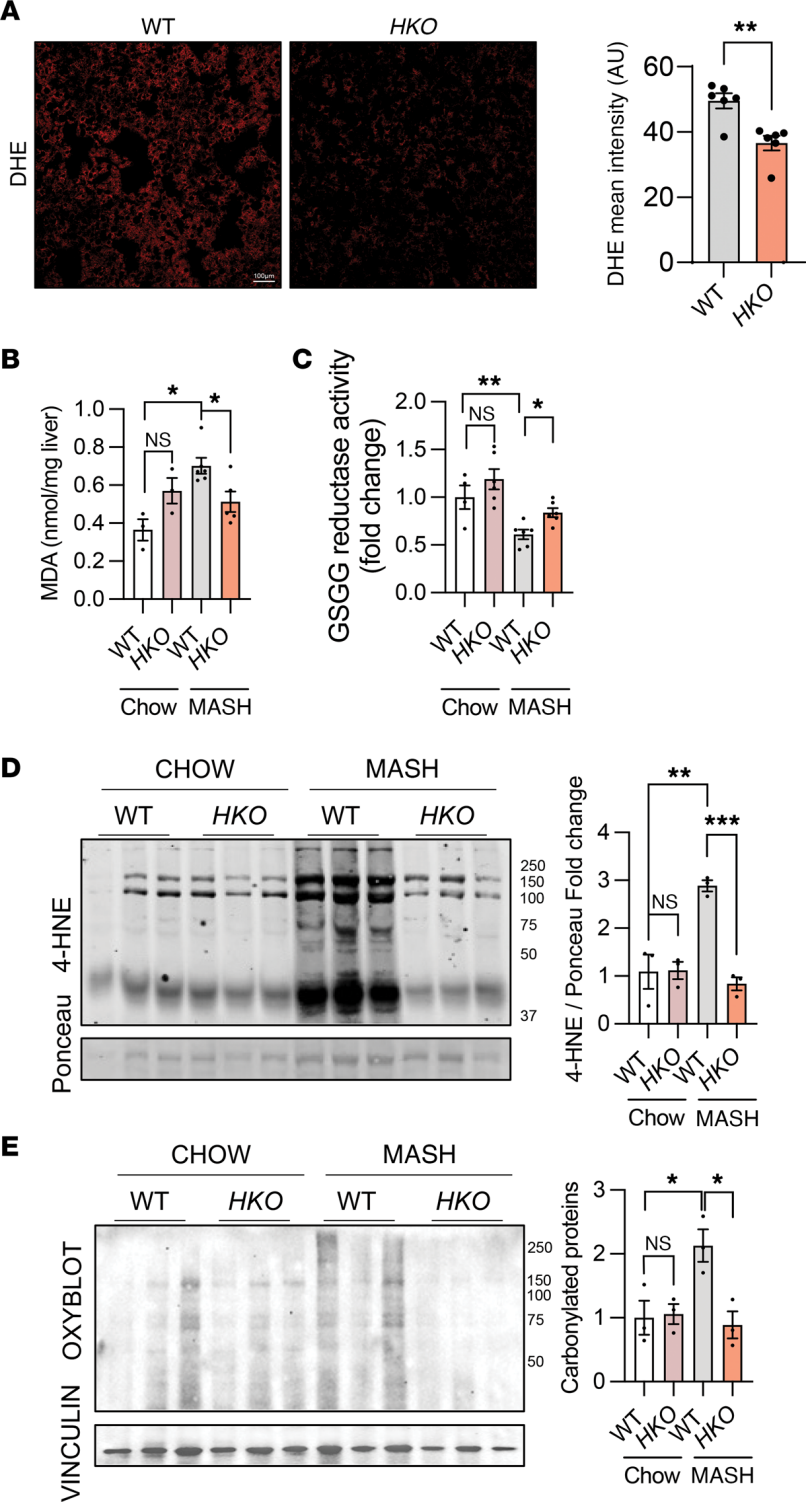
miR-33 *HKO* mice have reduced oxidative stress and cell death in MASH livers. (**A**) Representative DHE staining and quantification in WT and *HKO* livers from mice fed with CD-HFD for 6 months (*n* = 6 WT and 6 *HKO*). (**B**) Lipid peroxidation measured by MDA assay (Invitrogen) in MASH livers (*n* = 3 chow; 7 WT and 6 *HKO* MASH). (**C**) Glutathione reductase activity measured in MASH livers. Data represented as change in absorbance/min/μg of protein) (*n* = 4 WT and 6 *HKO* chow; 6 MASH). (**D**) Western blot and densitometric analysis of 4-HNE and (**E**) protein carbonylation measured by OxyBlot in WT and *HKO* livers. Data represent the mean ± SEM. *P ≤ 0.05, **P ≤ 0.01, ***P ≤ 0.001, compared with WT animals, unpaired 2-sided Student’s *t* test for 2-group comparisons (**A**) and 2-way ANOVA followed by multiple comparison (**B**–**E**).

Lipid overload and excessive mitochondrial activity have been linked with mitochondrial dysfunction in MASLD. Besides the inability to sustain metabolic needs, mitochondrial dysfunction is responsible for the production of large amounts of ROS, which increases mitochondrial damage and can eventually lead to cell death (76). Although the increased mitochondrial number and activity in *HKO* mice could lead to higher ROS production and damage, changes in mitochondrial dynamics can also play a role in ROS regulation, membrane potential, and other downstream processes related to mitochondrial stress (24, 74). To determine whether miR-33 levels in hepatocytes influence ROS production in obesity-driven MASLD/MASH, we monitored ROS accumulation in liver sections by dihydroethidium (DHE) and observed a decrease in *HKO* mice (Figure 9A). Liver lipid peroxidation measured by assessing malondialdehyde (MDA) as a readout of ROS damage also showed a similar decrease in livers from *HKO* mice (Figure 9B). Although no major changes were found in the oxidized or reduced forms of glutathione, or their ratio (Supplemental Figure 6B), an increase in glutathione-reductase (GSGG) activity was found in *HKO* livers, suggesting that changes in the recycling rather than the synthesis of glutathione may contribute to reduced oxidative stress in these livers (Figure 9C). Finally, oxidative stress markers including 4-hydroxynonenal (4-HNE), and OxyBlot, verified increased levels of oxidative stress in WT livers during MASH (Figure 9, D and E). Considering the close link among mitochondrial dynamics, lipid overload, and ER stress, we interrogated *HKO* MASH livers for changes in ER stress response. However, only partial regulation of ATF4 and protein kinase R-like ER kinase was found in those livers, indicating a limited involvement of miR-33 in the regulation of ER stress (Supplemental Figure 6C). Last, the ultimate cellular consequence of mitochondrial dysfunction and oxidative stress, the induction of cell death, was also attenuated in the *HKO* mice under MASH conditions, as seen by caspase-3/6 activity and TUNEL staining (Supplemental Figure 6, D–G). Notably, analysis of gene expression changes related to decreased hepatocyte cell death and increased mitochondrial function were also observed in *HKO* hepatocytes identified from scRNA-Seq (Supplemental Figure 6H). Together, these findings indicate that miR-33 deficiency in hepatocytes improves mitochondrial quality control by enhancing mitochondrial biogenesis and mitochondrial dynamics to sustain high rates of oxidative metabolism without increasing mitochondrial injury and oxidative stress during lipid overload, thereby protecting against hepatocyte cell death. The activation of mitochondrial biogenesis and dynamics in miR-33–deficient hepatocytes through upregulation of PGC1α and AMPKα is in line with previous studies identifying these 2 genes as direct targets of miR-33.

### AMPK signaling pathway is increased in miR-33 HKO livers.

**Figure 10:**
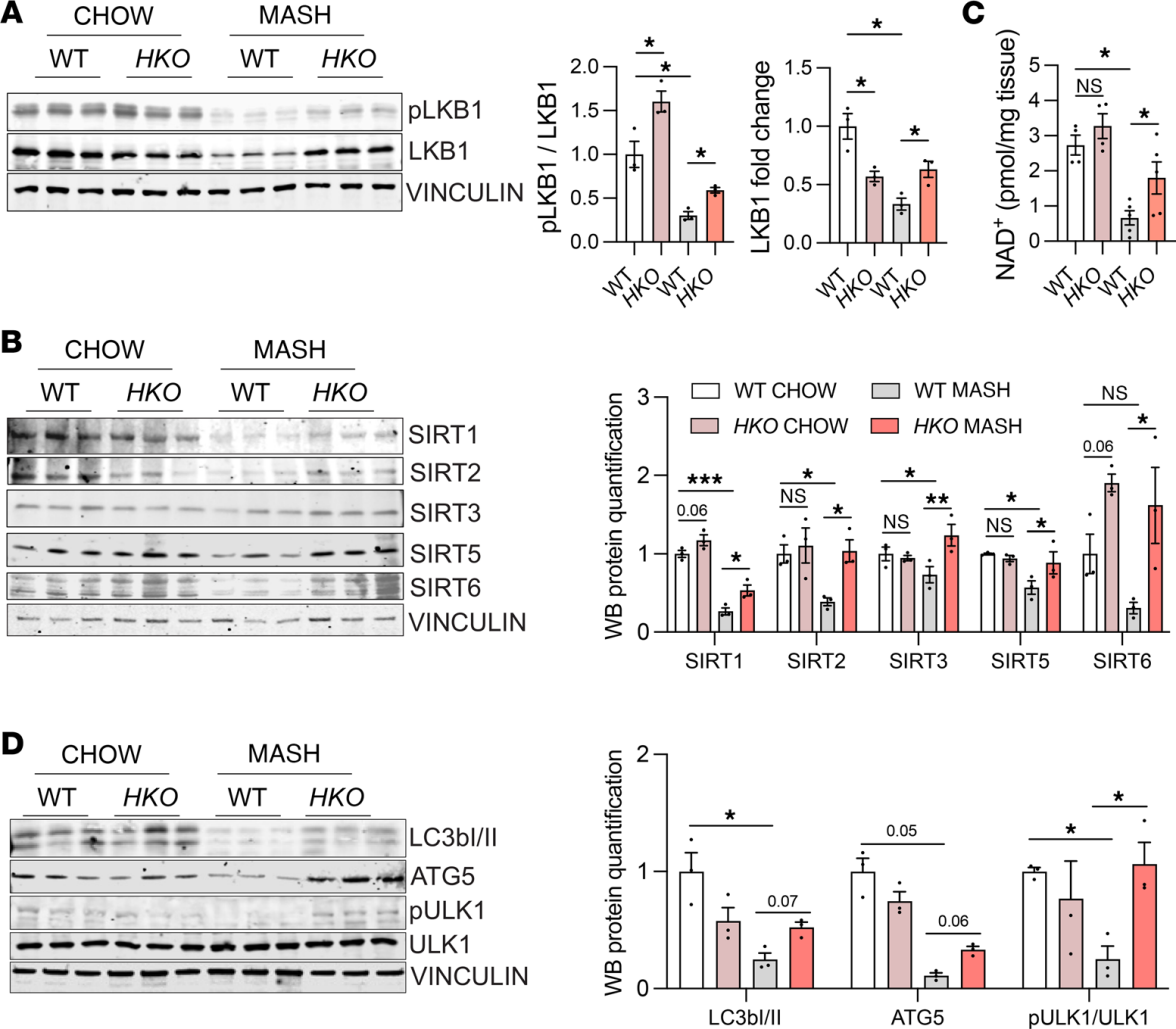
Upstream and downstream analysis of AMPKα signaling pathway. (**A** and **B**) Western blot analysis and densitometric analysis of housekeeping standard VINCULIN and (**A**) p-LKB1 (Ser428), LKB1, (**B**) SIRT1, SIRT2, SIRT3, SIRT5, SIRT6, and SIRT7 in WT and *HKO* livers from mice fed with CD-HFD for 6 months (MASH). (**C**) NAD^+^ levels in WT and *HKO* MASH livers represented as pmol/mg of tissue (*n* = 4 chow; 5 MASH). (**D**) Western blot analysis and densitometric analysis of p-ULK1 (Ser555), ULK1, LC3bI/II, ATG5, and housekeeping standard VINCULIN in WT and *HKO* MASH livers. Data represent the mean ± SEM (*P ≤ 0.05, **P ≤ 0.01, ***P ≤ 0.001 compared with WT animals, unpaired 2-sided Student’s *t* test for 2-group comparisons and 2-way ANOVA followed by multiple comparison).

AMPK is a master regulator of metabolism and mitochondrial homeostasis (77). Our previous results showed that AMPK activation is increased in *HKO* mice compared with WT mice in both MASL and MASH stages, counteracting the progressive decrease otherwise evident in MASLD (44). These results prompted us to characterize additional posttranscriptional mechanisms regulating AMPK in our model. Notably, we found that the activation of liver kinase B1 (LKB1), a kinase that controls AMPK activity, was enhanced as shown by the increased phosphorylation of LKB1 at serine 428 in *HKO* livers (Figure 10A). LKB1 activation is regulated by its subcellular compartmentalization through deacetylation and phosphorylation (78–80), correlating with increased levels of sirtuins, a family of histone and protein deacetylases. In accordance, we found increased levels of sirtuin 1 (SIRT1), SIRT2, SIRT3, and SIRT7 and a trend toward upregulation of SIRT6 in *HKO* livers (Figure 10B). Sirtuin activity is dependent not only on expression levels but also on the availability of NAD^+^. We found that total NAD and NAD^+^ as well as the NAD^+^/NADH ratio were increased in *HKO* livers (Figure 10C and Supplemental Figure 7A). These results point toward the increased activation of upstream regulators of AMPKα. As previously shown in Figure 2, we found increased FAO and decreased FAs, with increased AMPKα/ACC phosphorylation indicating a broad rewiring of metabolism mediated by AMPK signaling in *HKO* livers. Consistent with our observations on the effects of AMPK, we found that phosphorylation of ULK1, a downstream target of AMPKα, was also increased in *HKO* livers, along with the increased levels of LC3bII and ATG5, suggesting a role of AMPKα in the promotion of increased autophagy in *HKO* livers (Figure 10D). Likewise, *HKO* livers displayed increased phosphorylation of caspase-6, which has been described to be regulated by AMPK, consistent with reduced hepatocyte cell death in MASH (44) (Supplemental Figure 6F).

### miR-33 deficiency in hepatocytes reduces MASLD progression to HCC.

**Figure 11:**
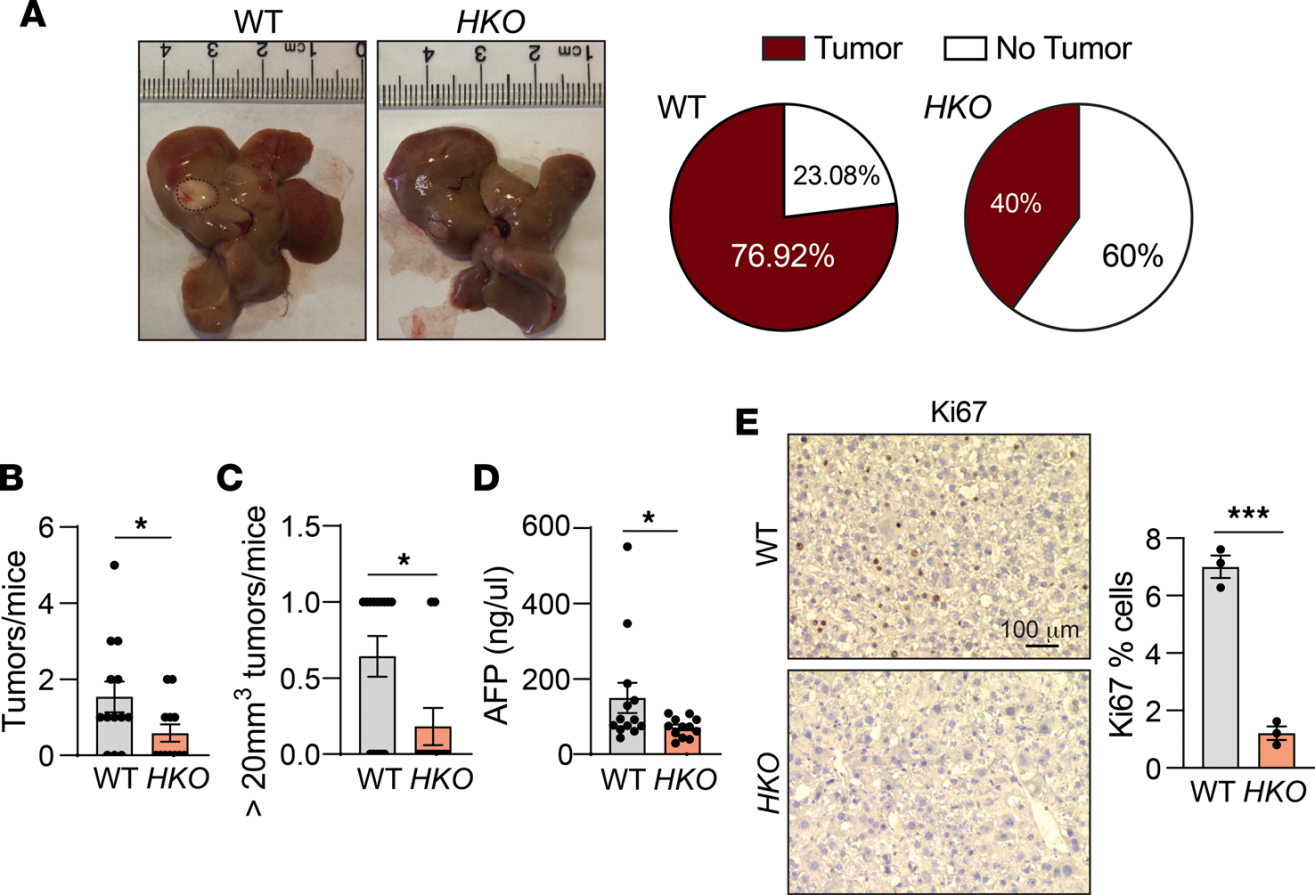
Hepatic miR-33 deficiency reduces diet-induced tumor incidence. (**A**) Representative images of WT and *HKO* livers after 15 months of CD-HFD; dashed line used to outline tumors and relative number of mice with and without tumor. (**B**–**D**) Graphical representation of (**B**) total number of tumors/mouse and (**C**) number of tumors larger > 20 mm^3^/mouse. (**D**) Circulating AFP levels (*n* = 13 WT –and 12 HKO). (**E**) Representative images of Ki67 staining in liver tumors from WT and *HKO* mice after 15 months of CD-HFD. Indicated quantification on the right (*n* = 3). Data represent the mean ± SEM (*P ≤ 0.05, ***P ≤ 0.001 compared with WT animals, unpaired 2-sided Student’s *t* test for 2-group comparisons and 2-way ANOVA followed by multiple comparison).

To analyze whether the improved metabolic function and protection against MASL-MASH progression attenuates the development of HCC in *HKO* mice, we fed WT and *HKO* mice a CD-HFD for 15 months. While 75% of WT mice developed tumors, only 40% of *HKO* mice did (Figure 11A). Tumor quantification revealed a decrease in the average tumor number per mouse in *HKO* mice (Figure 11B), which was particularly pronounced for large tumors (volume > 20 mm^3^) (Figure 11C). In agreement with reduced tumor incidence, serum levels of α-fetoprotein (AFP) were significantly reduced in *HKO* mice compared with WT mice (Figure 11D). Histological analysis of WT and *HKO* tumors also revealed a decrease in proliferative Ki67-positive cells in tumors from *HKO* mice compared with WT mice (Figure 11E). Analysis of miR-33 expression by qPCR in livers fed the CD-HFD compared with a standard chow diet for 15 months showed a modest increase in miR-33 expression in MASH, which was not statistically significant (Supplemental Figure 8A). Additionally, increased expression of miR-33 was detected in tumor samples when directly compared with adjacent healthy liver tissue from the same mice in WT samples (Supplemental Figure 8B), suggesting a potential role of miR-33 directly within the tumor microenvironment. Moreover, miR-33 levels correlated with increased levels of Ki67, a proliferation marker (Supplemental Figure 8, B–E).

**Figure 12:**
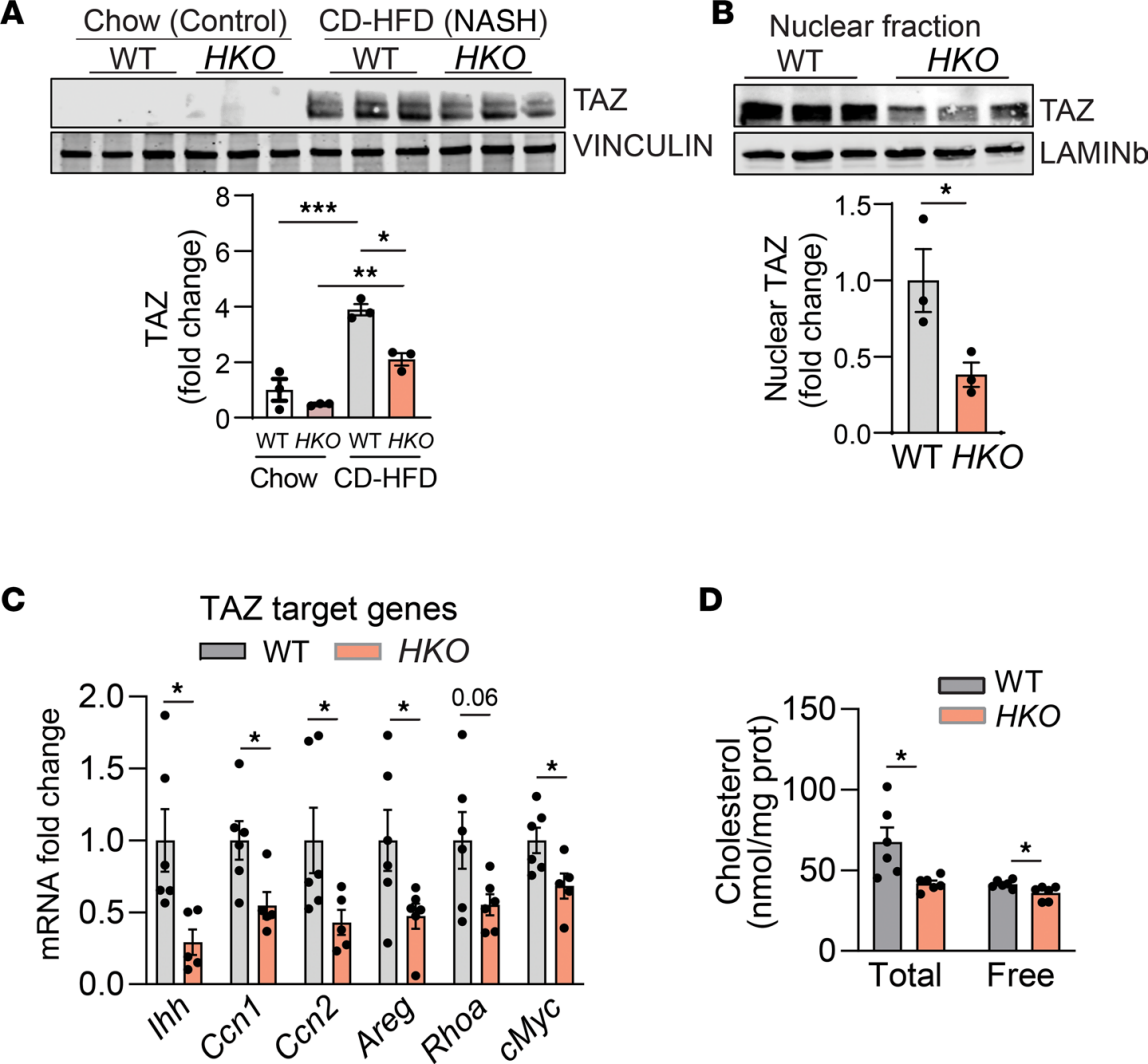
Hepatic miR-33 deficiency reduces Hippo signaling. (**A**) Western blot and densitometric analysis of TAZ and housekeeping standard VINCULIN in WT and *HKO* livers fed chow diet and CD-HFD for 6 months. (**B**) Western blot and densitometric analysis of TAZ and housekeeping standard LAMINb in nuclear fractions of livers from WT and *HKO* mice after 6 months of CD-HFD. (**C**) qPCR analysis of mRNA expression indicated genes in WT and *HKO* livers after 6 months of CD-HFD (*n* = 6 WT and 5 *HKO*). (**D**) Liver total and free cholesterol levels after 6 months of CD-HFD measured by gas chromatography–MS (*n* = 6). Data represented as nmol cholesterol/mg liver protein. Data represent the mean ± SEM. *P ≤ 0.05, **P ≤ 0.01, ***P ≤ 0.001 compared with WT animals, unpaired 2-sided Student’s *t* test for 2-group comparisons (**B**–**D**) and 2-way ANOVA followed by multiple comparison (**A**).

Recent studies have highlighted the role of cholesterol in the activation of the gene regulator TAZ and the importance of this for the severity of MASH and progression to HCC (45, 46, 49, 81, 82). YAP/TAZ are transcriptional coactivators of the Hippo pathway that participate in the initiation and progression of different cancers (82–84). Specifically, TAZ levels in HCC have been associated with its initiation and prognosis (49, 81, 85). TAZ upregulation in MASH and HCC has been associated with both increased cholesterol levels and decreased AMPKα activity, and it has been described to participate in the transcriptional regulation of several genes involved in fibrosis, proliferation, superoxide formation, and regulation of metabolism. Furthermore, its upregulation has been described in the pretumor MASH stage (49, 81, 85). Given the role of miR-33 as a key regulatory molecule that controls cholesterol efflux through the targeting of ABCA1 and ABCG1, we hypothesized that the absence of miR-33 in hepatocytes would decrease cholesterol content, potentially attenuating YAP/TAZ activation. Upregulation of TAZ levels in MASH livers was partially abrogated in mice lacking miR-33 in hepatocytes (Figure 12A and Supplemental Figure 8F). We further verified the activation of TAZ in MASH livers by cellular fractionation and immunoblotting for its nuclear localization (Figure 12B). In line with this, expression of downstream TAZ target genes was decreased in *HKO* livers (Figure 12C). In line with the role of miR-33 in the regulation of cholesterol homeostasis, a decrease in total and free cholesterol content was found the livers from *HKO* mice compared with WT mice fed with a CD-HFD (Figure 12D). To strengthen the potential link between liver cholesterol and miR-33 levels with YAP/TAZ activation, we cultured AML12 mouse hepatocytes in the absence or presence of LDL-cholesterol (120 μg/mL) and analyzed changes in YAP/TAZ signaling. Analysis of the regulation of YAP/TAZ protein levels verified that cholesterol loading of hepatocytes is sufficient to induce YAP/TAZ stability (Supplemental Figure 8, G and H). Thus, we aimed to determine whether the regulation of miR-33 in hepatocytes modulates YAP/TAZ activation in response to LDL-cholesterol. Overexpression of miR-33 in AML12 cells resulted in increased cholesterol content when cells were loaded with LDL-cholesterol (120 μg/mL) (Supplemental Figure 8, I and J). However, we did not observe differences in cholesterol-sensing genes (Supplemental Figure 8K). In line with the increased cellular cholesterol content, YAP/TAZ activation was increased in response to miR-33 overexpression, in accordance with the increased cellular content of cholesterol (Supplemental Figure 8L). Overall, these results suggest that miR-33 regulates YAP/TAZ activation in hepatocytes through the regulation of cholesterol content, linking cholesterol accumulation within the cells with activation of proliferative pathways associated with HCC.

**Figure 13:**
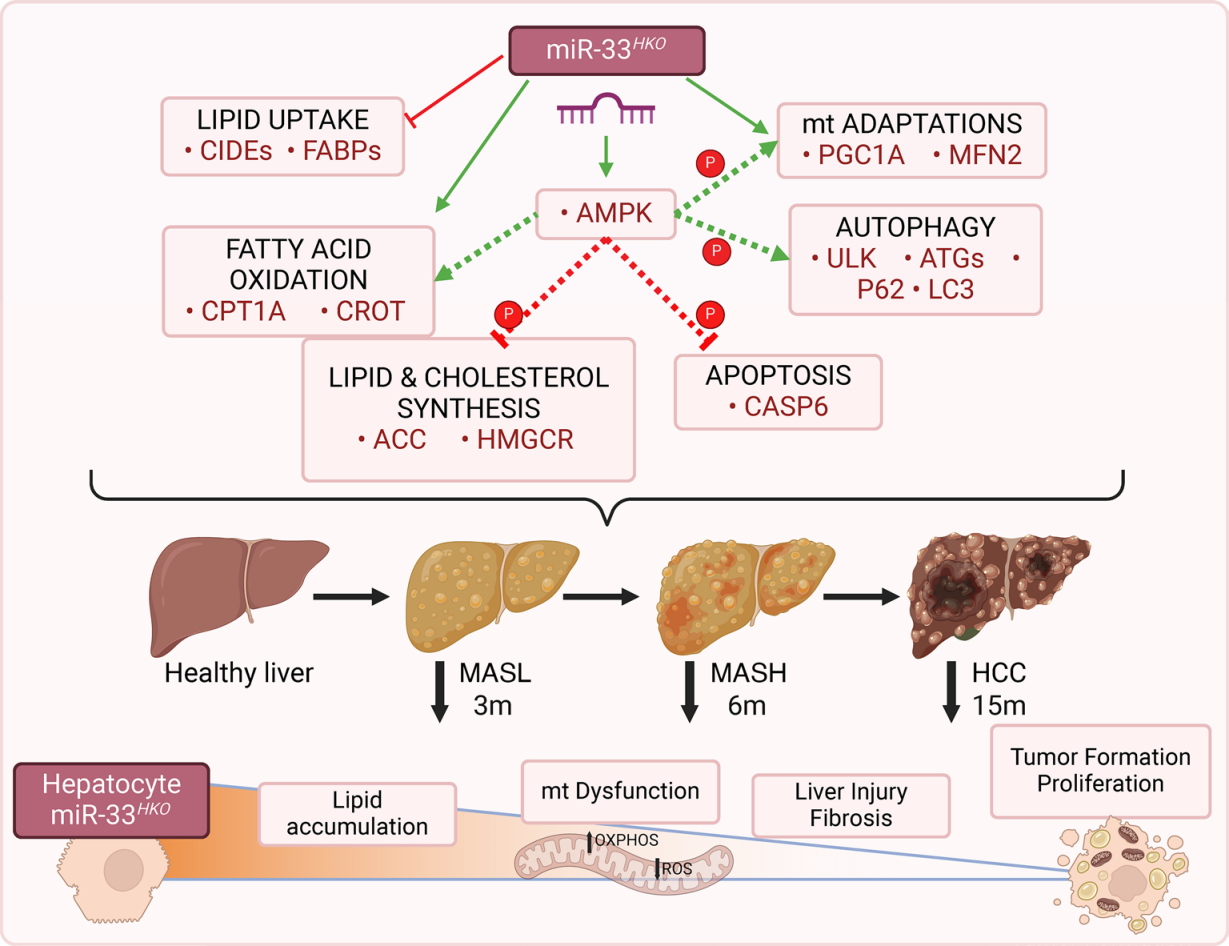
Schematic representation of probable miR-33 mechanisms of action

Taken together, our results suggest miR-33 deletion in hepatocytes improves mitochondrial metabolic function, restraining MASLD/MASH progression and in the long term preventing the development of HCC (Figure 13).

## Discussion

The rise in MASLD associated with overnutrition has reached epidemic levels, but its complexity has hindered the development of effective treatments. This study shows that miR-33 is increased in hepatocytes at different stages of MASLD in human patients and mouse models. Deletion of miR-33 in hepatocytes improves liver function, reducing lipid accumulation and progression of the disease. Numerous studies have demonstrated roles of miR-33 in metabolism, including cholesterol homeostasis, TAG metabolism, autophagy, glucose metabolism, and mitochondrial function (33–41), showing that inhibiting miR-33 reduces atherosclerosis in mice and nonhuman primates (37, 38, 86–91). However, due to the promiscuous nature of miRNAs, the whole-body deficiency of miR-33 has been associated with obesity, dyslipidemia, and insulin resistance (92, 93). These detrimental effects prompted us to study the role of miR-33 in other metabolic diseases, such as MASLD. Recent strategies have emerged to overcome potential undesired effects of miRNA therapies, shedding light on the cell-specific functions of miR-33 and its therapeutic potential. Some of these studies have demonstrated the efficiency of delivering miR-33 inhibitors inside pH low-insertion peptides to the kidney and atherosclerotic lesions (94, 95). Additionally, in a recent study, using a different strategy, our group also demonstrated the safety and efficiency of specifically removing miR-33 from hepatocytes to improve cholesterol and FA metabolism, underscoring the role of hepatic miR-33 in liver metabolism and fibrosis (50). This previous study showed not only that miR-33 suppression in hepatocytes was not responsible for the adverse metabolic effects observed in whole-body deficient mice but also that liver-specific loss of miR-33 improved whole-body metabolism under hyperlipidemic conditions (50). Building upon this model, our current work has focused on the metabolic advantages associated with miR-33 deficiency in hepatocytes, during MASLD-MASH-HCC development, to investigate the long-term alterations in liver function under this chronic inflammatory disease.

The initial characterization of miR-33 *HKO* mice in this CD-HFD model showed a clear impact on the regulation of cholesterol and glucose metabolism, with minimal effects on body weight, consistent with previous results found for *HKO* mice on other diets (50). The reduction in steatosis was associated with the modulation of several pathways involved in liver lipid accumulation, including FA uptake, DNL, and FAO. Interestingly, metabolic benefits observed in *HKO* mice were already observed after feeding the mice for 3 months with the CD-HFD and mostly sustained over the time, suggesting that early protection can prevent the progression of the disease. Taken together, our data suggest that the benefits resulting from miR-33 deletion in hepatocytes are apparent in the context of diet-induced obesity and MASLD. Minimal changes were observed in mice fed a standard chow diet, further suggesting that the improvements in liver metabolism that alleviate lipid buildup are primarily responsible for ameliorating liver injury and progression of the disease. Indeed, most predicted miR-33 targets upregulated in *HKO* livers were associated with known metabolic genes and functions. Additionally, our results suggest that miR-33 deletion in hepatocytes could affect BA metabolism and homeostasis. Although the lack of major changes in BA content in chow-fed WT and *HKO* livers was surprising given the role of miR-33 in regulating CYP7A1 and other BA transporters, the changes observed in MASH livers were in line with miR-33 playing a prominent role in the regulation of BA and cholesterol metabolism (54, 60).

One of the most common features in MASLD is the inability to sustain mitochondrial adaptation to nutrient status (18, 20–26, 75). Mitochondria are highly dynamic organelles with the ability to undergo functional and structural changes in response to environment and energy requirements (74). However, in MASLD, as with other metabolic diseases, increased FAO rates to counteract lipid accumulation are thought to result in increased oxidative stress and ER stress, resulting in mitochondrial injury and impaired oxidative phosphorylation (18, 26, 75, 96). The analysis of hepatic mitochondria from WT and *HKO* mice fed a CD-HFD for 6 months showed that miR-33 deficiency was associated with significant changes in mitochondrial quantity and morphology, suggesting a broader role of miR-33 in metabolism beyond regulation of cholesterol and FAO. The observed changes in mitochondria suggest miR-33 *HKO* mice have increased mitochondrial biogenesis and mitochondrial dynamics, mechanisms directed by PGC1α, AMPKα, and MFN2, among other markers, a phenotype associated with enhanced oxidative capacity coupled with reduced ROS production, resulting in protection from liver injury (24–26, 74). The approaches used here to study mitochondrial turnover and dynamics suggest a positive regulation of mitochondrial biogenesis and mitochondrial fusion; however, as these processes are related, we cannot exclude the 2 events being interdependent. Several studies have described the direct role of miR-33 in repressing mitochondrial biogenesis and FAO by targeting PGC1α and AMPKα, which are known to participate both in mitochondrial biogenesis and in regulation of fusion/fission processes, suggesting that miR-33 could directly regulate these functions in our model through the downregulation of these 2 targets. Despite employing different approaches to elucidate potential miR-33 target genes involved in the regulation of mitochondrial oxidative stress production or antioxidant pathways, no direct evidence of miR-33 action was found, suggesting that oxidative stress reduction is a consequence of improved mitochondrial biogenesis and dynamics.

The deficiency of miR-33 in hepatocytes was sufficient to protect from HCC development in our diet model. Previous studies aiming to determine the potential oncogenic role of miR-33 in HCC and cell proliferation have reported conflicting results, ranging from a role in inhibiting cell proliferation to antioncogenic roles of miR-33 in HCC cell lines. Similarly inconsistent results were reported in murine models and human samples of HCC, muddling the conclusions about the role of miR-33 in HCC (97–100). While the higher levels of miR-33 detected in tumor samples compared with adjacent healthy tissue and their correlation with Ki67 suggest that miR-33 could be directly involved in promoting tumor progression, the direct role of miR-33 within these tumors should be the subject of further research. Finally, our results align with previous studies showing that YAP/TAZ activation in hepatocytes plays a central role in liver fibrosis and the transition to hepatocellular carcinoma in response to increased cellular cholesterol levels (46). Given the role of miR-33 in cholesterol regulation, we hypothesized that miR-33 could also be contributing to attenuation of HCC development through this mechanism. Cell culture experiments in AML12 hepatocytes indicate the involvement of miR-33 and cholesterol accumulation in activation of YAP/TAZ pathway in hepatocytes. However, given the multiple adaptations regulated in *HKO* mice, the decrease observed in YAP/TAZ activation could also be a consequence of AMPKα phosphorylation.

This study contributes to a deeper understanding of the mechanisms involved in MASLD-MASH progression and the potential applicability of miR-33 therapeutic approaches for its treatment. The beneficial or detrimental effects of enhanced FAO in MASLD/MASH have been extensively discussed in the past, and our present results bring insight into the beneficial role of FAO in the disease (15, 25, 101, 102). Our findings indicate that the regulation of hepatocyte metabolism by miR-33 is involved in the progression of MASLD/MASH, as well as MASLD/MASH-derived HCC. The direct deletion of miR-33 in hepatocytes protects from the progression of this disease. This may be particularly relevant for the use of approaches such as *N*-acetylgalactosamine–conjugated antisense oligonucleotides, which have been demonstrated to effectively promote targeted delivery of inhibitors to the liver (103). In the context of human pathology, it is important to note that while mice possess only the miR-33a isoform of miR-33, humans express both miR-33a and miR-33b isoforms, encoded within the *SREBF2* and *SREBF1* genes, respectively, which are regulated by different mechanisms (104, 105). Recent observations of the transcriptional activity of both *SREBF1* and *SREBF2* in both murine and human MASLD (52) suggest that in humans, miR-33b may also contribute to the development of the disease, supporting the therapeutic potential of targeting hepatic miR-33 in human pathology.

This work demonstrates that the depletion of miR-33 in the hepatocytes is sufficient to increase hepatic metabolic activity and reduce lipid accumulation in the liver protecting from MASLD and MASH. However, in this study, we did not determine whether a pharmacological approach to revert the progression of the disease would have a similar impact. Future studies should investigate whether pharmacological approaches can replicate the metabolic benefits observed in this study by selectively inhibiting miR-33 in hepatocytes. Although scRNA-Seq data did not reveal major alterations in miR-33 target genes in cell types other than hepatocytes, it will be important to confirm if the Albumin-Cre used in this study might affect miR-33 expression in liver cholangiocytes. While the study focused on changes in described and predicted miR-33 target genes, future investigations should assess the interaction of miR-33 with mRNA in hepatocytes under MASLD conditions to better understand the precise mechanisms involved. Finally, this study suggests that the protection from HCC development is mainly mediated through the steady long-term effect of miR-33 deletion in hepatocytes. However, based on our data, we cannot discard a potential intrinsic role of miR-33 within the tumor microenvironment, promoting tumor growth and proliferation. Further exploration is needed to elucidate the exact role of miR-33 within the tumor in the context of obesity-induced HCC. By addressing these avenues, future research can deepen our understanding of the intricate roles of miR-33 in liver metabolism and potential therapeutic interventions for MASLD, MASH, and HCC. These investigations will not only advance our knowledge of molecular mechanisms but also pave the way for targeted precision medicine approaches in the clinical management of these complex liver diseases.

## Methods

### Sex as a biological variable.

Animals included in this study involved male mice. In our previous studies (50, 106) we have demonstrated how miR-33 conditional knockout in the liver and other tissues has similar effect in male and females. Thus, sex was not considered a biological variable, and for ethical and availability reasons, studies were performed only in males. Results are expected to be relatable to both sexes. For human liver biopsies, liver core biopsies were from obese men and women undergoing bariatric surgery, have been described previously (107), and were processed for RNA isolation. Sex difference analyses were not performed due to the low frequency of suitable donors.

### Animals.

miR-33–KO mice (*miR-33^loxP/loxP^*) were generated as previously described (50, 106). To generate *HKO* mice, *miR-33^loxP/loxP^* mice were bred with transgenic mice expressing *Cre* recombinase under the control of a hepatocyte-specific promoter: albumin promoter (The Jackson Laboratory stock 003574). To produce diet-induced liver disease, mice were fed a standard chow diet until 8 weeks of age. Then chow diet was replaced by a modified CD-HFD, containing 45% of fat and no choline added (D05010402, Research Diets). Mice were maintained with CD-HFD feeding for 3, 6, or 15 months to induce steatosis, MASH, or HCC at respective time points (51). Body weight was measured throughout diet feeding studies, and analysis of body composition was performed by Echo MRI (Echo Medical System). All mice were sex and age matched and kept in individually ventilated cages in a pathogen-free facility. Mice were fasted for 6 hours at end time point experiments.

### Statistics.

All data are expressed as mean ± SEM unless indicated. Statistical differences were measured using unpaired 2-sided Student’s *t* tests or 2-way ANOVA. Normality was checked using the Kolmogorov-Smirnov test. A value of *P* ≤ 0.05 was considered statistically significant. Data analysis was performed using GraphPad Prism Software Version 9.0. Specific statistics used for scRNA-Seq and downstream analysis are detailed in Supplemental Methods.

### Study approval.

Animal experiments were conducted under the ethical guidelines of, and protocols approved by, the IACUC at Yale University School of Medicine (animal protocol 2019-11577). The use of human tissue was approved by the Monash University Human Research Ethics Committee (CF12/2339-2012001246; CF15/3041-2015001282). All participants gave their written informed consent before participating in this study.

### Data availability.

All Supporting data values are provided in the Supporting Data Values file. RNA-Seq data have been deposited in the National Center for Biotechnology (NCBI) Gene Expression Omnibus (GEO) database (GSE220093). scRNA-Seq data have been deposited in the NCBI GEO database (GSE259277).

## Author contributions

PFT, YS, and CFH designed the research. PFT, MPC, HZ, JS, NLP, NEB, LG, MCS, CEX, WB, and OPR performed research and analyzed data. XY, RAH, AMB, and TT analyzed data and edited the manuscript. PFT and CFH wrote the manuscript.

## Supplementary Material

Supplemental data

Unedited blot and gel images

Supporting data values

## Version 1 08/27/2024

In-Press Preview

## Version 2 10/08/2024

Electronic publication
