# Supplementary material for: miR-33 deletion in hepatocytes attenuates MASLD-MASH-HCC progression
Source: JCI Insight. 2024 Aug 27;9(19):e168476. doi: 10.1172/jci.insight.168476 (PMC11466198; doi:10.1172/jci.insight.168476)
Supplement: Supplemental data [file jciinsight-9-168476-s186.pdf]

miR-33 deletion in hepatocytes attenuates NAFLD-NASH-HCC progression

SUPPLEMENTAL MATERIAL

RNA isolation and Real-Time qPCR. Total RNA, including miRNAs, was isolated from liver tissue or hepatocytes using the miRNeasy miRNA Isolation Kit according to the manufacturer's instructions and reverse-transcribed into cDNA with iscript cDNA systhesis kit (Bio-Rad, USA). qPCR was performed with SsoFast EvaGreen Supermix (Bio-Rad) using the same thermal profile conditions for all primers sets: 40 amplification cycles (95 °C for 5 s and 60 °C for 10 s). All samples were analyzed in duplicate and normalized to 18S. To analyze miR-33, RNAs were reverse-transcribed with the miRCURY LNA RT Kit (Qiagen, Germany) and quantified with the miRCURY LNA miRNA PCR assays (Qiagen). miR-33 levels were normalized to the levels of SNORD68 RNA.

Liver Histology. Mouse livers were perfused with PBS and fixed in 4% paraformaldehyde (PFA) overnight. Then livers were transferred to 70% for immunohistochemistry (IHC) analysis and submitted for staining through the Yale Pathology Tissue Services core, with H&E, Sirius Red, F4/80, TUNEL and Ki67 to analyze liver morphology, fibrosis, inflammation, cell death and proliferation. Additionally, an independent piece of liver was transferred from PFA to 30% sucrose for lipid staining with Oil Red O. For each staining, pictures were taken with an EVOS microscope. Hepatocyte ballooning was quantified as relative number of ballooned hepatocytes per frame. Abundance of macrovesiclular fat, Sirius Red, F4/80 and Oil Red O staining were quantified by number or area percentage with ImageJ software. TUNEL and Ki67 were quantified as number of positive nuclei per total number of nuclei.

Western Blot analysis. Tissues were homogenized by manual disruption and the Bullet Blender Homogenizer in ice-cold buffer containing 50mMTris·HCl, pH 7.5, 0.1% sodium dodecyl sulfate (SDS), 0.1% deoxycholic acid, 0.1 mM EDTA, 0.1 mM EGTA, 1% Nonidet P-40, 5.3 mM NaF, 1.5 mM NaP, 1 mM orthovanadate, 1 mg/mL protease inhibitor mixture (Roche), and 0.2mg/mL 4-(2-aminoethyl) benzenesulfonyl fluoride hydrochloride (Roche). Lysates were sonicated and rotated at 4 °C for 1 h before the insoluble material was removed by centrifugation at 12,000 Å~ g for 10 min. After normalizing for equal protein concentration, cell lysates were resuspended in SDS sample buffer before separation by SDS polyacrylamide gel electrophoresis. Following transfer of the proteins onto nitrocellulose membranes, the membranes were probed with the following antibodies: CROT (Novus no. 3144; 1:1,000), CPT1A (Abnova H00001374-P01; 1:1,000), ACC (Ser79) (CST #11818; 1:1000) , ACC (CST #3676; 1:1000), AMPK (T712) (CST #2535; 1:1000), AMPKα (CST #5831; 1:1000), AMPKβ1(Ser182) (CST #4186; 1:1000), AMPKβ1 (CST #4150; 1:1000), FN1 (Sigma F3648; 1:1000), COL1a1 (Novus Biologicals NB600; 1:500), Total OXPHOS cocktail (Abcam ab110413; 1:2000), PGC1α (Abcam ab54481; 1:1000), TFAM (Abcam ab252432; 1:1000), MFN1 (Abcam ab126575; 1:1000), MFN2 (Abcam ab56889; 1:1000), OPA1 (CST #80471; 1:1000), DRP1 (CST #8570; 1:1000), MFF1 (Ser146) (CST #49281; 1:1000), MFF1 (CST #84580; 1:1000), C6 (Ser257), C6 (CST #9762; 1:1000), LKB1 (Ser428) (CST #3482; 1:1000), LKB (CST #13031; 1:1000), SIRT1 (CST #9475; 1:1000), SIRT2 (CST #12650; 1:1000), SIRT3 (CST #5490; 1:1000), SIRT5 (CST #8782; 1:1000), SIRT6 (CST #12486; 1:1000), SIRT7 (CST #5360; 1:1000), ULK1 (Ser555) (CST #5869; 1:500), ULK1 (Novus Biologicals NBP2-24738SS; 1:1000), LC3bI/II (CST #12741; 1:1000), (CST #39749; 1:1000),, ATG5 (CST #12994; 1:1000), YAP/TAZ, YAP (Ser127) (CST #13008; 1:1000), TAZ(Ser89) (CST #59971; 1:1000), GAPDH (Abcam ab8245; 1:5000), LAMINb (Abcam ab19109; 1:5000), VINCULIN (Sigma V9131; 1:5000), 4-Hydroxynonenal (Abcam ab46545). For preparation of cytosol and nuclear extracts from liver tissue the Nuclear Extract Kit (ab219177, Abcam, UK) was used following manufacturer's recommendation. Subsequent WB analysis was performed regularly.

Lipoprotein profile and circulating lipid measurement. Mice were fasted for 6h before blood samples were collected cardiac puncture, and plasma was separated by centrifugation. HDL-C was isolated by

precipitation of non-HDL cholesterol and both HDL-C fractions and total plasma were stored at –80 °C. Total plasma cholesterol and triglycerides were measured using kits according to the manufacturer's instructions (Wako Pure Chemicals, Japan). The lipid distribution in plasma lipoprotein fractions was assessed by fast performance liquid chromatography (FPLC) gel filtration with 2 Superose 6 HR 10/30 columns (Pharmacia Biotech, Sweden).

Glucose and Insulin tolerance test. GTTs were performed after overnight fasting (16h) by intraperitoneal (IP) injection of glucose at a dose of 1.5g/kg. Blood glucose was measured at 0-, 15-, 30-, 60- and 120-minutes post injection. ITTs were performed following 6h fasting by IP injection of 1.5U/kg of insulin. Blood glucose was measured at 0-, 15-, 30-, 60- and 120-minutes post injection.

Liver triglyceride and cholesterol measurements. For determining total TAGs content in the liver, TAGs were extracted using a solvent chloroform/methanol (2:1). TAG level in the liver was determined by using a commercially available assay kit (Sekisui Chemical Co., USA) according to the manufacturer's instructions. Determination of liver total and free cholesterol was determined gas-chromatography coupled with mass spectrometry (GC-MS). Briefly, liver tissue extracts equivalent to 1 mg/mg.Prot. were mixed with 1.5 ml of methanol/chloroform (Cl3CH) mixture (2:1, v/v) in presence of μ25 L of aqueous KOH 50% (w/v). Cholestanol (5α-cholestan-3β-ol, Sigma-Aldrich, USA) was added on every sample as internal standard. After incubation for 1h at 90ºC, the saponified lipid extract containing the total cholesterol was extracted with 1 mL of Cl3CH and 2 mL of water, the lower phase recovered and dried over nitrogen current. Cholesterol was analyzed by GC-MS as described previously (1). The same extraction protocol, without the addition of KOH, was done to extract free cholesterol from liver tissues.

Fatty acid oxidation (FAO). Ex vivo FAO was analyzed using [^{14}C] palmitate, as previously described (2). Briefly, livers were isolated from WT and *HKO* and homogenized in five volumes of chilled STE buffer (10 mM Tris-HCl, 0.25M sucrose, and 1 mM EDTA and pH 7.4). Homogenate was centrifuged, and the pellet was incubated with reaction mixture (0.5 mmol/L palmitate conjugated to 7% BSA/[^{14}C]-palmitate at 0.4μCi/ml) for 30 minutes. After this incubation, the resuspended pellet containing the reaction mixture was transferred to an Eppendorf tube, the cap of which housed a Whatman filter paper disk that had been presoaked with 1 mol/L sodium hydroxide. The ^{14}C trapped in the reaction mixture media was then released by acidification of media using 1 mol/L perchloric acid and gentle agitation of the tubes at 37C for 1h. Radioactivity that had become absorbed onto the disk was then quantified by liquid scintillation counting in a ß-counter.

***De novo* lipogenesis (DNL).** 250-300 mg of liver was homogenized on ice in 1 mL of a reaction mixture that contained Krebs-Ringer buffer (136 mM NaCl, 4.7 mM KCl, 1.2 mM CaCl, 20 mM HEPES, pH 7.4) with 0.5 mM acetate, 25 mM glucose and 3% BSA (Fatty-acid free). The homogenate was transferred to a 5 mL plastic culture tube and an additional 1 mL of the reaction mixture that contained 2 µci of [^{14}C]-acetate was added to the mixture. The tube was sealed, and the contents were incubated in an orbital shaker (200 RPM) at 37°C for 3 hours. Following the incubation, the mixture was stored at -80°C until lipid extraction. 30 μL of the solution were used for protein quantification. Neutral lipids were extracted from the remainder of the homogenate in chloroform/methanol (2:1, v/v) and purified using 0.9% NaCl by standard Folch extraction. Following extraction, the solution was dried under nitrogen gas in liquid scintillation tubes. The lipids were then mixed with 4mL liquid scintillation fluid and counted on a liquid scintillation counter (Perkin Elmer) for 3 minutes. Data was corrected for background and normalized by the protein concentration.

Fatty acid and Cholesterol synthesis. FASN activity was determined in the liver, as described previously with some modifications (3). Briefly, liver from WT and *HKO* mice were homogenized in tissue homogenization buffer (0.1 M Tris, 0.1 M KCl, 350 μM EDTA, and 1 M sucrose, pH 7.5) containing protease inhibitor cocktail (Roche, USA). The supernatant was collected by centrifuging liver homogenates at 9,400 g for 10 minutes at 4°C. For determining FASN activity, liver homogenate was

added to NADPH activity buffer (0.1 M potassium phosphate buffer, pH 7.5 containing 1 mM DTT, 25 µM acetyl-CoA, and 150 µM NADPH). Malonyl-CoA (50 µM) was added to the assay buffer to initiate the reaction. 340 absorbance was measured for 30 mins at an interval of 1 min (37°C). HMGCR Activity Assay assay was performed as described previously (4).Briefly, a microsomal fraction from cell lysates and the liver homogenate was obtained via ultracentrifugation (100000 x g for 60 min). The reaction buffer (0.16 M potassium phosphate, 0.2 M KCl, 400 µM EDTA, and 0.01 M dithiothreitol) containing 100 µM NADPH and microsomal protein (200 µg/mL) was prewarmed at 37 ºC for 10 min before the reaction. The reaction was initiated by adding 50 µM substrate (HMG-CoA) to the reaction buffer. Absorbance at 340 nm was followed for 30 min with an interval of 1 min.

MitoStress test. Real-time of oxygen consumption rate (OCR) was measured using a Seahorse XF24 Extracellular Flux Analyzer (Seahorse Biosciences, USA) as previously described (5). Briefly, WT and *HKO* primary hepatocytes from CD-HFD fed mice at the indicated time points were isolated by the Yale Liver Center by standard liver perfusion and collagenase digestion followed by centrifugation of the cell suspension at 60g for 4 minutes to pellet hepatocytes. Hepatocytes were then cultured in collagen type I coated XF24 cell culture microplates (Seahorse Bioscience) at 1.5 x10^4 cells per well and incubated 4-6 h at 37 °C. After that, the cells were washed once in 1x PBS and media was changed to low-glucose assay media for overnight incubation at 37 °C. The next morning hepatocytes were washed twice with 1 ml XF Assay Media (DMEM base containing 1 mM pyruvate, 2 mM glutamine and 5.5 mM glucose, pH 7.4) and incubated at 37 °C for 1 h in a non-CO2 incubator. Cells were then assayed on a Seahorse XFe24 Analyzer following a 12-min equilibration period. Respiration rates were measured using an instrument protocol of 3-min mix, 2-min wait, and 3-min measure. The following inhibitors were used at the indicated concentration: oligomycin (1 µM); carbonyl cyanide 4-trifluoromethoxyphenylhydrazone (FCCP) (1 µM); and rotenone (0.5µM)/antimycin (0.5µM). Flux rates were normalized to total protein content following cell lysis at the end of the assay.

RNA-sequencing. Total RNA from livers of control and HKO mice was extracted and purified using a RNA isolation Kit (Qiagen) followed by DNAse treatment to remove genomic contamination using RNA MinElute Cleanup (Qiagen). The purity and integrity of total RNA sample was verified using the Agilent Bioanalyzer (Agilent Technologies, Santa Clara, CA). rRNA was depleted from RNA samples using Ribo-Zero rRNA Removal Kit (Illumina, USA). RNA libraries were performed TrueSeq Small RNA Library preparation (Illumina) and were sequenced for 45 cycles on Illumina HiSeq 2000 platform (1 x 75bp read length). The reads obtained from the sequencer are trimmed for quality using inhouse developed scripts. The trimmed reads were aligned to the reference genome using TopHat2. The transcript abundances and differences were calculated using cuffdiff. The results were plotted using R and cummeRbund using in-house developed scripts. RNA-sequencing data have been deposited in the Gene Expression Omnibus database (GSE220093).

Single-cell RNA sequencing.
a) Preparation of single-cell suspension. Hepatic single-cell suspensions were prepared for submission for 10X single-cell RNA-sequencing and flow cytometry. Livers were perfused firstly with digestion cocktail (1.5mg/mL Collagenase I and 0.5mg/mL DNase I) from the portal vein. Isolated suspensions were centrifuged at 60G for 2 minutes with no brakes to separate parenchymal and non-parenchymal (NPC) fractions. For parenchymal fractions, a 35% Percoll gradient was used to enrich for live cells after spinning at 500g for 5 mins. To enrich for live NPCs, cells were stained with LIVE/DEAD Fixable Aqua Dead Cell Stain Kit before sorting on the FACSAria II Cell sorter. Purified parenchymal and NPC fractions were mixed at a 1:1 ratio before submission for single-cell sequencing.
b) Pre-processing of single-cell RNA-seq data. Single-cell RNA data from this project were processed using CellRanger software (version 7.1) as previously described(6). First, sample demultiplexing, aligning read to the mouse genome (University of California Santa Cruz mm10 reference genome) with Software Tools for Academics and Researchers (STAR) and unique molecular identifier (UMI) processing was

performed. The raw gene expression matrix was filtered with the following criteria. Cells with over 25% mitochondrial gene expression in UMI counts were removed; cells with under 300 detected genes were removed; cells with more than 33,470 UMI were removed. After filtering, a total of 21,920 cells from WT and 11,550 *HKO* were identified for further analysis.

c) Dimension reduction, unsupervised clustering, and cell cluster annotation. Processed gene expression matrix with all retained cells for each sample was imported to the Seurat R package (v4.3.0.1.) for downstream analyses (7). Data were normalized using the 'NormalizeData' function. 2000 most variable genes were identified using the 'FindVariableFeatures' function with the 'vst' method. High variable genes were applied for the principal component analysis (PCA) to identify the top 30 principal components using the 'RunPCA' function of Seurat, which was then applied to dimension reduction using the 'RunUMAP' function in Seurat. Uniform Manifold Approximation and Projection (UMAP) visualization indicated cells from different samples were well mixed into the shared space (8). For clustering, we applied a FindNeighbor graph using Principal components (PCs) 1 to 30 and k=25 nearest neighbors, and then the FindClusters clustering algorithm (1.5 resolution) was used to group the cells into different clusters. Cell clusters were annotated based on top differentially expressed marker genes and mapped to established cell signatures.

d) Identification of differentially expressed genes & pathway enrichment analysis. Differentially expressed genes in different clusters were identified by the 'FindAllMarkers' function in Seurat using the 'MAST' test and setting min.pct=0.25. Heatmaps for the differentially expressed genes were generated by customized R code using ggplot2 (v3.4.2, R package). Gene Set Enrichment Analysis (GSEA) was used to carry out analysis for pathways with differentially expressed genes across samples. Ingenuity Pathway Analysis (Ingenuity Systems QIAGEN, content version: 47547484, 2019) was used to determine pathways with differentially expressed genes across samples. Only genes with an average log2FC > 0.25 and a $P < 0.05$ were included for Ingenuity Pathway Analysis studies.

e) Ligand-receptor cell communication analysis. To identify potential intercellular interactions between different cell types, we utilized the R package CellChat version 1.6.012. In brief, CellChat object was created by feeding the processed Seurat object into the Createcellchat() function and processed using its standard pipeline. CellChatDB.mouse was loaded and differentially expressed genes and interactions were identified in the CellChat object via IdentifyOverExpressedGenes and IdentfyOverExpressedInteractions, respectively. The CellChat algorithm was then run to calculate the communication probability and infer cellular communication network via ComputeCommunProb and ComputeCommunProbPathway.

OxyBlot Analysis. OxyBlot analysis was performed with the OxyBlot Protein Oxidation Detection Kit (Milipore, S7150), in fresh frozen liver lysates according to manufacturer's protocol. Whole SMIR bands was normalized to VINCULIN.

ALT Measurements. ALT activity was determined in serum with the ALT Activity Assay Kit (Sigma-Aldrich MAK052) following the manufacturer's recommendations.

Liver Flow Cytometry analysis. A small piece (250-300 µg) of PBS perfused liver was resected and placed in 2ml cold PBS, then clopped into smaller pieces by mechanical disruption. The homogenate was then transfer into a gentleMACS C Tube and further dissociated with a gentleMACS Dissociator (Program liver 1, x2). Homogenate was digested with liver digestion buffer (5 ml HBSS w/o Ca^{2+}/Mg^{2+}, 200U/ml Collagenase IV (Worthington, USA) (37 °C, 30mins, ~60-80 rpm). After digestion, homogenate was filtered through 70 µm filter, washed with 10 ml blocking buffer (HBSS w/o Ca^{2+}/Mg^{2+}, 2% FBS, 5mM EDTA) and centrifuged at 500g, 5mins, RT. Pellet was resuspended in 10 ml 20% Percoll and centrifuged 1300g, 30 mins, RT. 13. Pellet was resuspended in 1mL of HBSS blocking solution and transferred to a 1.5 ml Eppendorf tube, then centrifuged at 500g, 5 mins, RT. Final pellet was resuspended in 200 µl of ACK (155 mM ammonium chloride, 10 mM potassium bicarbonate, and 0.01 mM EDTA, pH 7.4) and then stained with a mixture of antibodies. B cells were identified as APC-Cy7 B220 (Biolegend, USA); T cells were identified as $CD4^{hi}$ or $CD8^{hi}$ with the following antibodies: BUV395 CD90.2 - 565257 (BD, USA),

BV711 CD4 - 100447 (Biolegend), BV605 CD8a - 100744 (Biolegend) and activation was determined according to CD62L/CD44 status with PE-Cy7 CD62L - 25-0621-82 (eBioscience, USA), BUV737 CD44 - 612799 (BD), BV605 CD8a - 100744 (Biolegend); macrophages were identified as FITC F480 - 157310 (Biolegend); neutrophils were identified as $CD11b^{hi}Ly6G^{hi}$ with Pacific Blue CD11b - 101224 (Biolegend), APC Ly6G - 127614 (Biolegend). All antibodies were used at 1:300 dilutions.

Blood Flow Cytometry analysis. Blood was collected by heart puncture. For FACS analysis, erythrocytes were lysed with ACK lysis buffer (155 mM ammonium chloride, 10 mM potassium bicarbonate, and 0.01 mM EDTA, pH 7.4). White blood cells were resuspended in 3% fetal bovine serum (FBS) in PBS, blocked with 2 µg mL−1 of FcgRII/III, then stained with a mixture of antibodies. Monocytes were identified as $CD115^{hi}$ and subsets as $Ly6\text{-}C^{hi}$ and $Ly6\text{-}C^{lo}$; neutrophils were identified as $CD11b^{hi}Ly6G^{hi}$; B cells were identified as $B220^{hi}$; T cells were identified as $CD4^{hi}$ or $CD8^{hi}$. All antibodies were used at 1:300 dilutions.

Bile Acid measurements. 200mg of frozen liver samples were homogenized in 50% methanol using a rotor-stator homogenizer. Deuterated BA standard (20 mL of 25 mM d4-cholic acid) was added to 200 mL of each sample, and calibrator curves were generated for each BA in charcoal-stripped tissue. To each sample/calibrator, 2 mL of ice-cold acetonitrile was added, then samples were vortexed for 1 hour at 2000 rpm and centrifuged for 10 minutes at 11 000g. Supernatants were transferred to a clean glass tube and dried down at 45 °C under N2. Each sample/calibrator was extracted a second time in 1 mL of ice-cold acetonitrile, vortexed for 1 hour at 2000 rpm and centrifuged for 10 minutes at 11 000g. The supernatant of the second extraction was combined with the first and dried down at 45 °C under N2. Each sample was resuspended in 200 mL of 55:45 (vol/vol) methanol:water, both with 5 mM ammonium formate. Samples were centrifuged in UltraFree MC 0.2-mm centrifugal filters (Millipore) and transferred to LC-MS vials, and 10 mL were injected into ultraperformance LC-tandem MS vials (Waters Corporation, Milford, MA, USA).

Liver Hydroxyproline assay. Hydroxyproline content in the liver was measured with the following protocol. Liver tissue (20-40mg) was homogenized in 6N HCl (1:20 w:v) and incubated 16-24h at 110 ºC. On the next day, livers were cooled down and filtrated with a 0.22 µm syringe filter. Homogenates were then neutralized with 2.2% NaOH in Citrate-Acetate-Buffer at a 1:10 ratio. Reaction was set as follows: 500 µl of samples was incubated with 250 µl of chloramine-T (20 min, RT), then 250 µl of PCA were added (20 min, RT), and 250 µl of Dimethylbenzaldehyde were added to the mixture and incubated (20 min, 60 ºC). Absorbance was measured at 565 nm.

DHE. Dihydroexiethidium staining was performed in 8µm sections from OCT-embedded livers. Sections were incubated with MnTBAP (150µM, 1h, RT), stained with DHE (Sigma-Aldrich) (5µM, 30 min, 37 ºC) and mounted with VECTASHIELD(R) Antifade Mounting Medium (Vector Laboratories, USA). Stained area percentage was calculated using ImageJ software.

Lipid Peroxidation. Liver lipid peroxidation was measured with the Lipid Peroxidation (MDA) assay kit MAK085 (Sigma-Aldrich), following manufacturer's recommendations for colorimetric detection at 532 nm.

Glutathione Reductase Activity. The reductase activity of glutathione was calculated in liver homogenates as the reduction of GSGG observed in the presence of NADPH. Briefly, livers were homogenized in assay buffer (0.2 M KPH, pH 7.0, 2 mM EDTA). Assay was performed to measure changes in NADPH absorbance at 340 nm. Reaction buffer was prepared as follows: 100 µl of assay buffer was added with 10 µl GSSG (20mM) and 10 µl NADPH (2 mM) and brought to a final volume of 200 µl with H_2O. Sample was added to reaction solution and absorbance at 340 nm was monitored for at least 30 mins. Changes in absorbance were normalized to protein concentration in liver lysates.

NAD/NADH. NAD/NADH were measured in liver samples with the NAD/NADH Assay Kit ab65348 (Abcam, UK) following manufacturer's recommendations.

Electron Microscopy and Mitochondrial Analysis. Sample preparation and imaging was performed by the Center for Cellular and Molecular Imaging (CCMI) Electron Microscopy Core Facility at Yale University. Briefly, Liver pieces were fixed with 2.5% glutaraldehyde and 2% PFA in 0.1 M sodium cacodylate (pH 7.4) for 2 h, RT. Cells were postfixed in 1% OsO_4 in the same buffer for 1 h, then stained en bloc with 2% aqueous uranyl acetate for 30 min, dehydrated in a graded series of ethanol to 100%, and embedded in Poly/bed 812 for 24 h. Thin sections (60 nm) were cut with a Leica ultramicrotome and poststained with uranyl acetate and lead citrate. Digital images were taken using a Morada charge-coupled device camera fitted with iTEM imaging software (Olympus). Mitochondria analysis was performed as described previously described (9). Mitochondria cross-sectional area and mitochondria aspect ratio (major axis divided by minor axis, minimum value is 1.0) were calculated as a measurement of mitochondria size and shape, respectively. Probability plots were utilized to estimate changes in mitochondria size and shape, and statistical differences were tested using Kolmogorov–Smirnov test. Mitochondria density was estimated by dividing the number of mitochondria by the cytosolic area. Mitochondria coverage was estimated by dividing the total area of mitochondria by the cytosolic area.

ETC activity Complex I and Complex II. Frozen liver tissues were homogenized with 20mM KP buffer (pH 7.4) with a glass homogenizer and centrifuged at 800g, 10 mins. Enzyme activities were measured at 30 ºC, monitoring the reaction for at least 2 h and normalizing the changes in absorbance to CS activity and protein concentration. Complex I activity was measured by tracking the oxidation of NADH at 340 nm in 20mM KP buffer (pH 8).0) with 200 μM NADH, 1mM NaN_3, 0.1% BSA-EDTA and 100 μM ubiquinone-1 with and without rotenone (5 μM), to calculate the rotenone-sensitive rate of NADH oxidation. Complex II was assayed following the reduction of 2,6-dichlorophenolindophenol (DCPIP) at 600 nm. Reaction was set in 50 mM Tris-KP (pH 7.0), with 1.5 mM KCN, 100 μm DCPIP and 32 mM succinate. Citrate synthase (CS) activity was measured at 420 nm in 75 mM Tris-HCl (pH 8.0) buffer with 100 μM DTNM, 350 μg/ml acetyl-CoA, 0.5 mM oxalacetate and 0.1% Triton X-100).

Caspase-3 and Caspase-6. Caspase-3 activity was assayed as previously described (10). Briefly, livers protein lysates were incubated with fluorescent substrate Ac-DEVD-AMC Caspase-3 Fluorogenic Substrate (BD Biosciences) at 37°C and the reaction was monitored for at least 4 h to track changes in fluorescence (λ excitation of 390 nm and λ emission of 510 nm). For Caspase-6 activity assay, similar procedure was followed for colorimetric detection with Caspase-6 Colorimetric Assay Kit K115 (BioVision, USA), monitoring absorbance at 405 nm.

AFP Measurements. AFP levels in serum were determined with the AFP kit - MAFP00 (R&D, USA) following manufacturer's recommendations.

Cell culture studies. Mouse *alpha mouse liver 12* (AML12) (CRL-2254) cells were purchased from AATC. Cells were cultured for three passages before being subject to experimental treatment. AML12 cells were maintained in DMEM-F12 (Thermo Fisher Scientific, USA) media supplemented with 10% FBS (RndSystems), 1% penicillin/streptomycin (Gibco), insulin and dexamethasone, under 5% CO_2 at 37° C in a humidified incubator. For cholesterol enrichment assays, cells were cultured in the same media with 120 μg/ml native LDL (nLDL) and 2μM of the ACAT1 inhibitor Sandoz 58-035 (CAS 78934-83-5, Santa Cruz) to inhibit the esterification of cholesterol. Cholesterol in cells was measured with the AmplexRed commercial kit (Invitrogen) following manufacturer's recommendations.

REFERENCES

1. Araldi E, Fernández-Fuertes M, Canfrán-Duque A, Tang W, Cline GW, Madrigal-Matute J, et al. Lanosterol Modulates TLR4-Mediated Innate Immune Responses in Macrophages. *Cell Rep.* 2017;19(13):2743-55.
2. Huynh FK, Green MF, Koves TR, and Hirschey MD. Measurement of fatty acid oxidation rates in animal tissues and cell lines. *Methods Enzymol.* 2014;542:391-405.
3. Bays NW, Hill AD, and Kariv I. A simplified scintillation proximity assay for fatty acid synthase activity: development and comparison with other FAS activity assays. *J Biomol Screen.* 2009;14(6):636-42.
4. Kleinsek DA, Ranganathan S, and Porter JW. Purification of 3-hydroxy-3-methylglutaryl-coenzyme A reductase from rat liver. *Proc Natl Acad Sci U S A.* 1977;74(4):1431-5.
5. Fernández-Tussy P, Fernández-Ramos D, Lopitz-Otsoa F, Simón J, Barbier-Torres L, Gomez-Santos B, et al. miR-873-5p targets mitochondrial GNMT-Complex II interface contributing to non-alcoholic fatty liver disease. *Mol Metab.* 2019;29:40-54.
6. Zheng GX, Terry JM, Belgrader P, Ryvkin P, Bent ZW, Wilson R, et al. Massively parallel digital transcriptional profiling of single cells. *Nat Commun.* 2017;8:14049.
7. Stuart T, Butler A, Hoffman P, Hafemeister C, Papalexi E, Mauck WM, 3rd, et al. Comprehensive Integration of Single-Cell Data. *Cell.* 2019;177(7):1888-902 e21.
8. Becht E, McInnes L, Healy J, Dutertre CA, Kwok IWH, Ng LG, et al. Dimensionality reduction for visualizing single-cell data using UMAP. *Nat Biotechnol.* 2018.
9. Dietrich MO, Liu ZW, and Horvath TL. Mitochondrial dynamics controlled by mitofusins regulate Agrp neuronal activity and diet-induced obesity. *Cell.* 2013;155(1):188-99.
10. Fernández-Ramos D, Fernández-Tussy P, Lopitz-Otsoa F, Gutiérrez-de-Juan V, Navasa N, Barbier-Torres L, et al. MiR-873-5p acts as an epigenetic regulator in early stages of liver fibrosis and cirrhosis. *Cell Death & Disease.* 2018;9(10):958.

SUPPLEMENTAL FIGURES

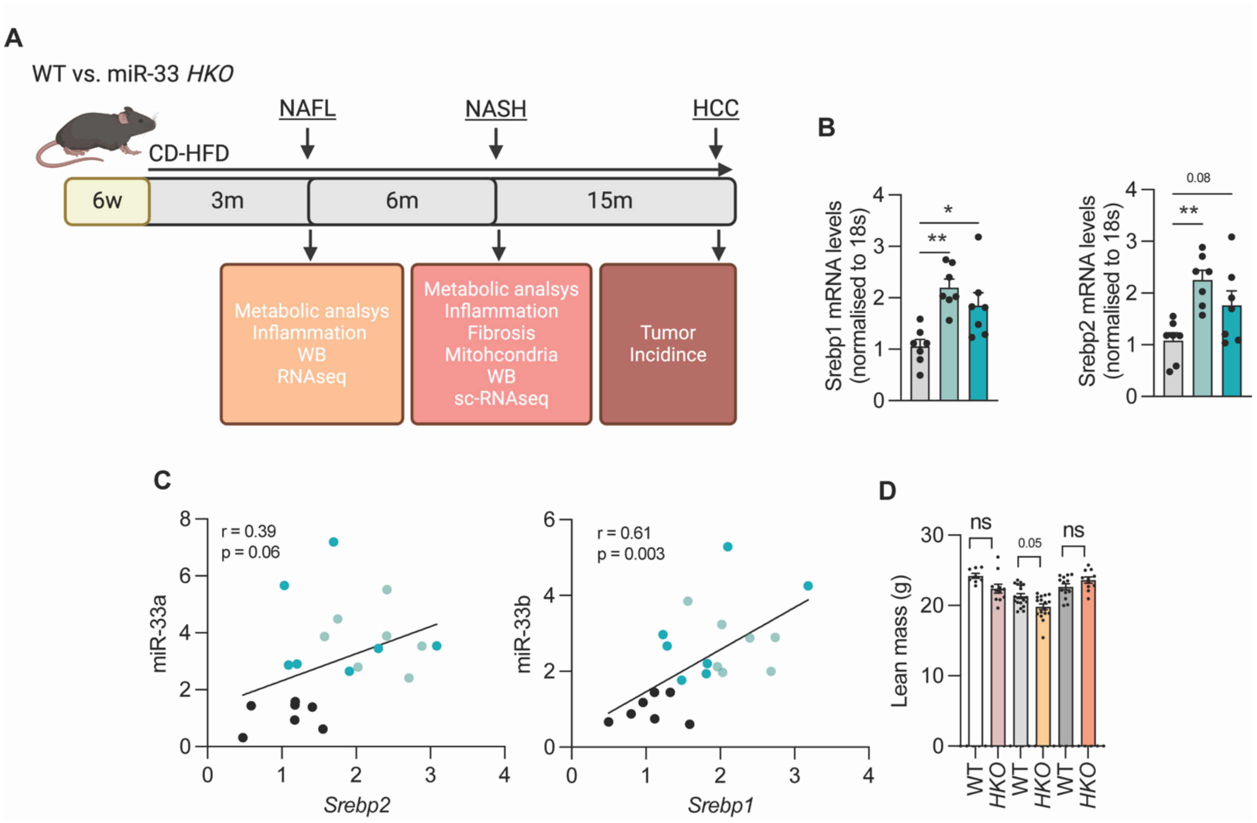

Supplemental Figure 1. *SREBF1* and *SREBF2* are upregulated in human NAFL NASH livers and correlates with *miR-33a/b* expression. **(A)** Schematic representation of the experimental design to analyze whole NAFLD-NASH-HCC progression from steatosis/NAFL to NASH to HCC in WT and hepatocyte specific miR-33 knockout (*HKO*) mice fed with CD-HFD for 3-,6- and 15-months. **(B)** mRNA expression levels of *Srebp1* and *Srebp2* in livers from human NAFL and NASH patients compared to healthy human livers. **(C)**. Pearson's correlation of miR-33a/*Srebp2* and miR-33b/*Srebp1*. (**D**) Lean mass analysis measured by MRI. Data represent the mean ± SEM (*P ≤ 0.05 compared with healthy condition, One-Way ANOVA Tukey's multiple comparison.

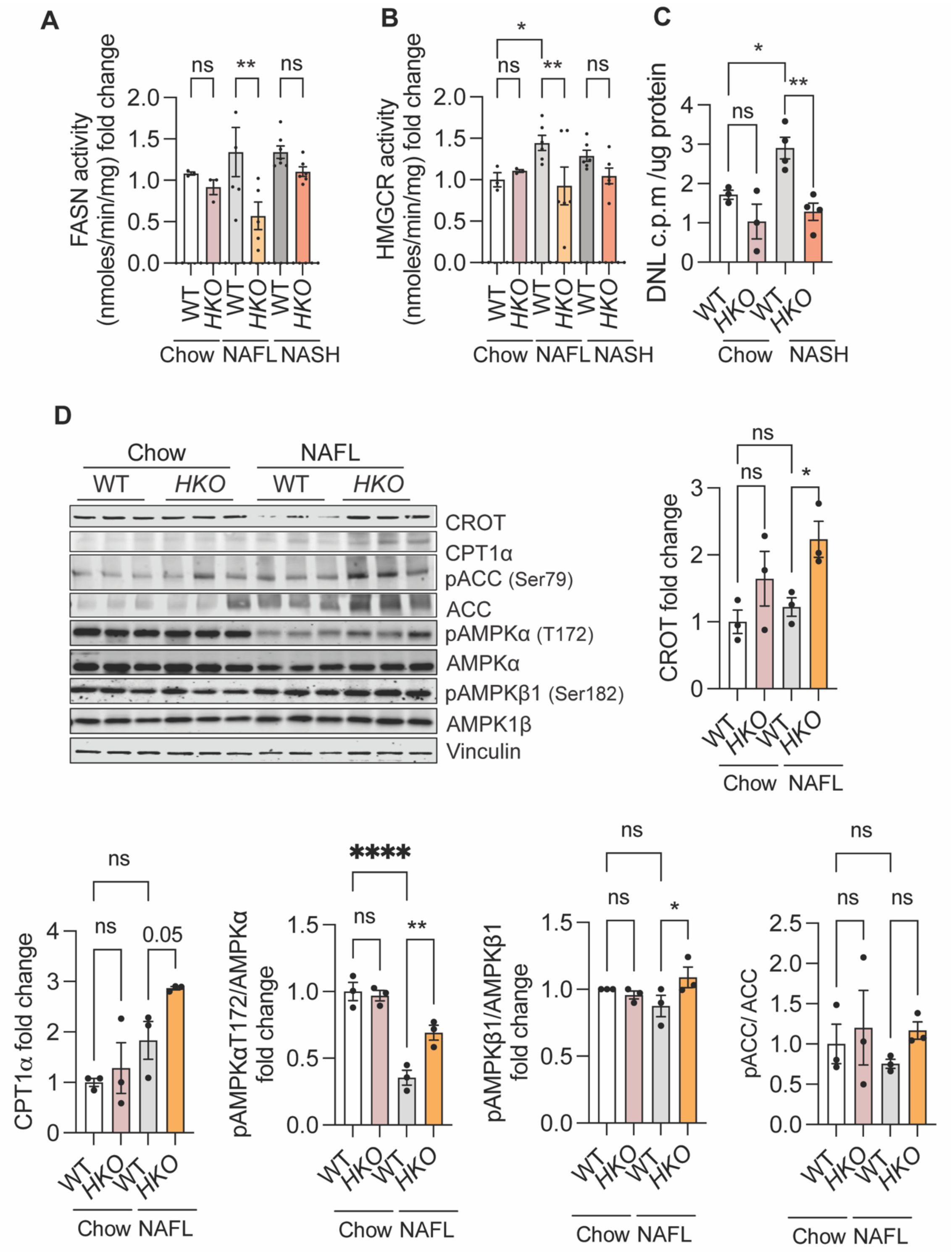

Supplemental Figure 2. CD-HFD model in WT and *HKO* mice. (A,B) Enzymatic activity of FASN and HMGCR in WT and *HKO* liver microsomes. **(C)** Measurement of *de novo lipogenesis* (DNL) in WT and *HKO* livers. **(D)** Western blot and densitometric analysis of CROT, CPT1α, pACC (Ser79), total ACC, pAMPKα (T172), total AMPKα, pAMPKß (Ser182), AMPKß and housekeeping standard VINCULIN in WT and *HKO* livers during NAFL. Data represent the mean ± SEM. *P ≤ 0.05, **P ≤ 0.01, ***P ≤ 0.001 compared with WT animals, comparisons and 2-way ANOVA followed by multiple comparison.

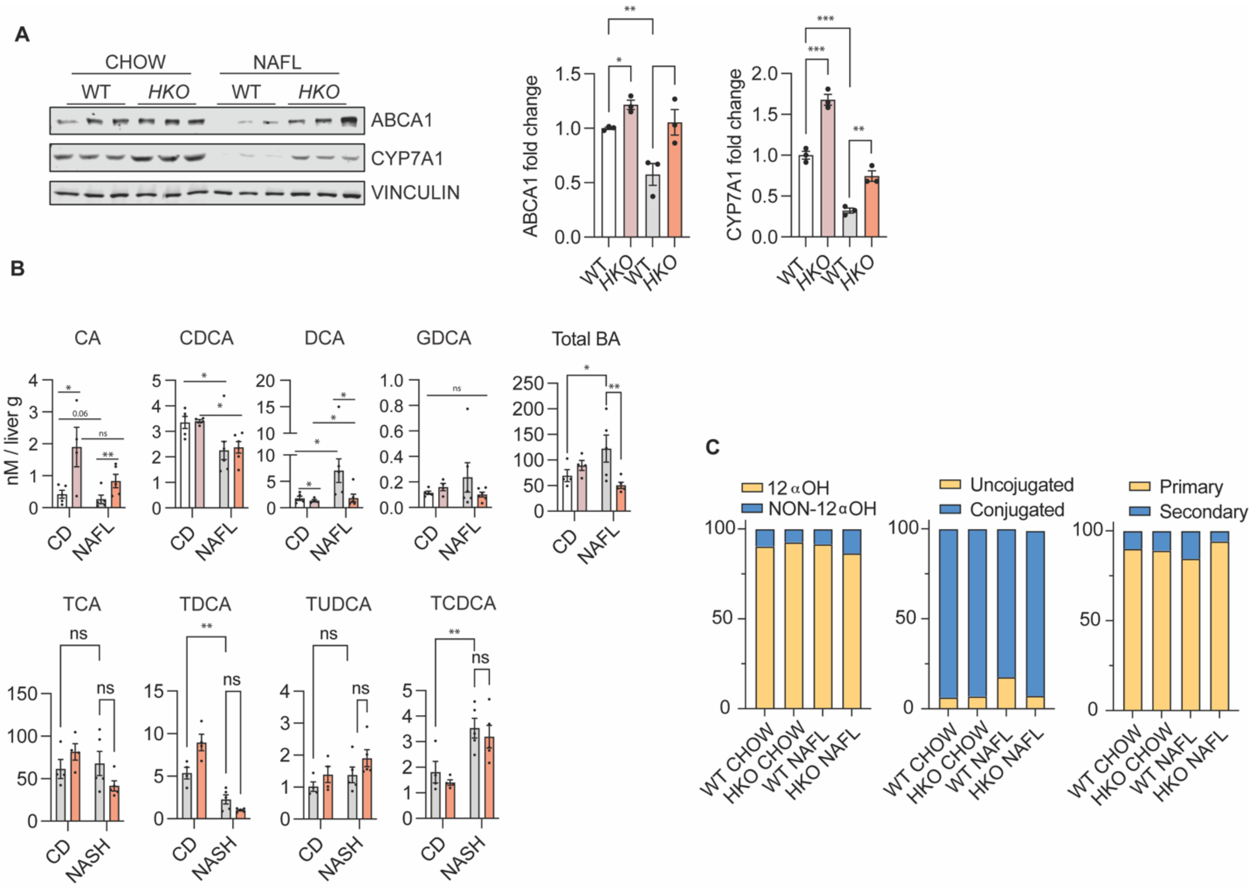

Supplemental Figure 3. miR-33 regualtes bile acid composition in the liver in NAFLD. (A) Western blot and densitometric analysis of ABCA1 and CYP7A1 and housekeeping VINCULIN in in WT and *HKO* livers. **(B)** Measurement of liver bile acid content of indicated species and unconjugated/conjugated ratio and secondary/primary ratio in mice WT and HKO mice fed a chow or CD-HFD (CA: cholic acid; CDCA: Chenodeoxycholic acid; DCA: Deoxycholic acid; GDCA: Glycodeoxicholic acid; TCA: Taurocholic acid; TDCA: Taurodeoxycholic acid; Tauroursodeoxycholate; TCDCA: Taurochenodeoxycholic acid); MCA: muricholic acid. **(C)**. Representation of bile acid distribution in livers as classified by 12α-hydroxy/non–12α-hydroxy ratio, unconjugated/conjugated ratio and secondary/primary ratio. *P ≤ 0.05, **P ≤ 0.01, ***P ≤ 0.001 compared with WT animals, comparisons and 2-way ANOVA followed by multiple comparison.

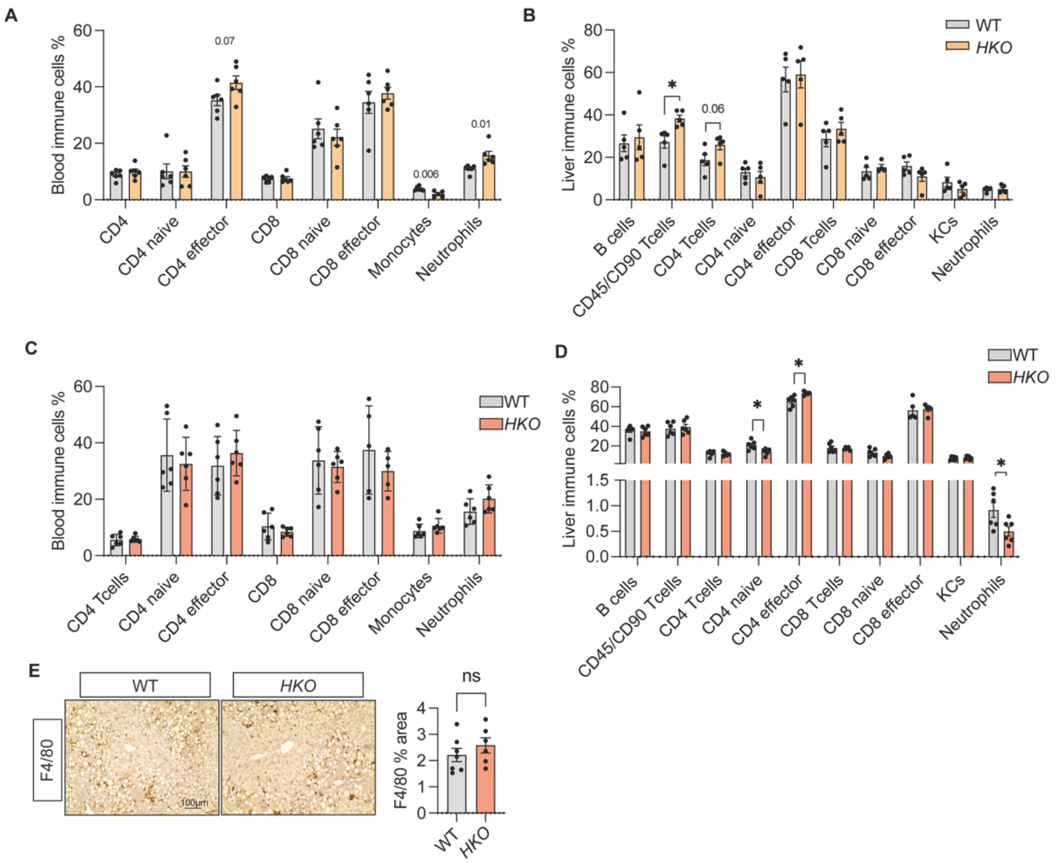

Supplemental Figure 4. Analysis of inflammatory markers in NAFL and NASH. Flow cytometry analysis of blood **(A** and **C)** and liver **(B** and **D)** leukocytes from WT and hepatocyte miR-33 knockout (*HKO*) mice at 3 **(A, B)** and 6 **(C, D)** months of CD-HFD feeding. **(E)** Representative images of F4/80stained livers from WT and *HKO* mice. Indicated quantification on the right. Data are expressed as percentages of live cells. Data represent the mean ± SEM. *P ≤ 0.05 compared with WT animals, unpaired Student's *t* test.

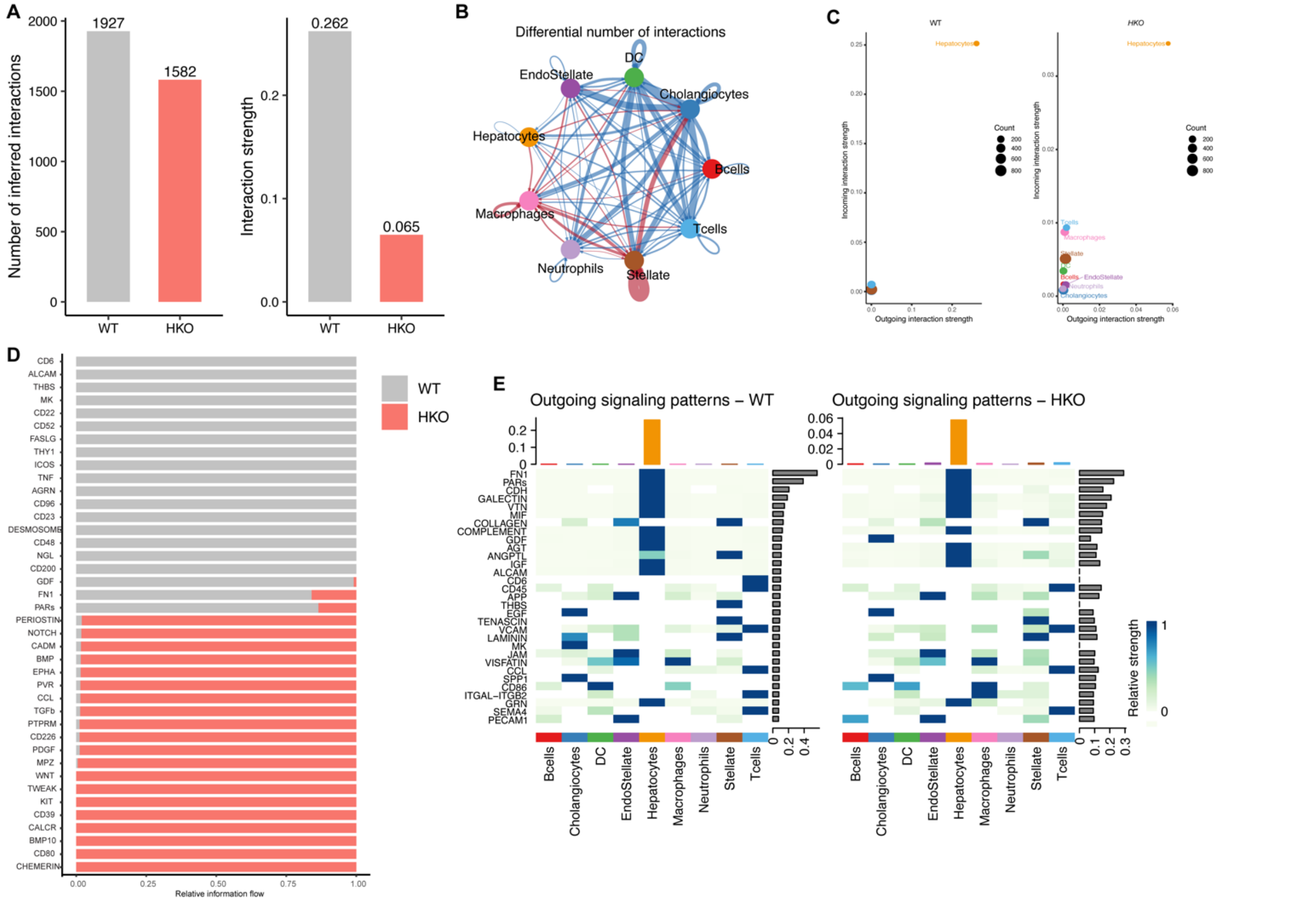

Supplemental Figure 5. Impact of miR-33 *HKO* in intracellular communication network in the liver niche during NASH defined by single-cell RNA-sequencing. (A) Comparison of the total number of interactions and interaction strength between WT and *HKO* mice. **(B)** Circle plot highlighting the differential number of ligand-receptor (L-R) interactions between WT and *HKO* mice. **(C)** Scatter plots comparing the outgoing and incoming interaction strength in the 2D space between WT and *HKO* mice. **(D)** Comparison of the signaling pathway enriched in WT and *HKO* mice based on the relative information flow between pairwise datasets. **(E)** Heatmap displaying outgoing signaling patterns. The scale above the heatmap represents the overall signaling strength in each cell type. The horizontal grey bars to the right of the heatmap depicts the signaling strength of each signaling pathway from all cell type in the liver. The color gradient represents the relative contribution of a cell type to the pathway.

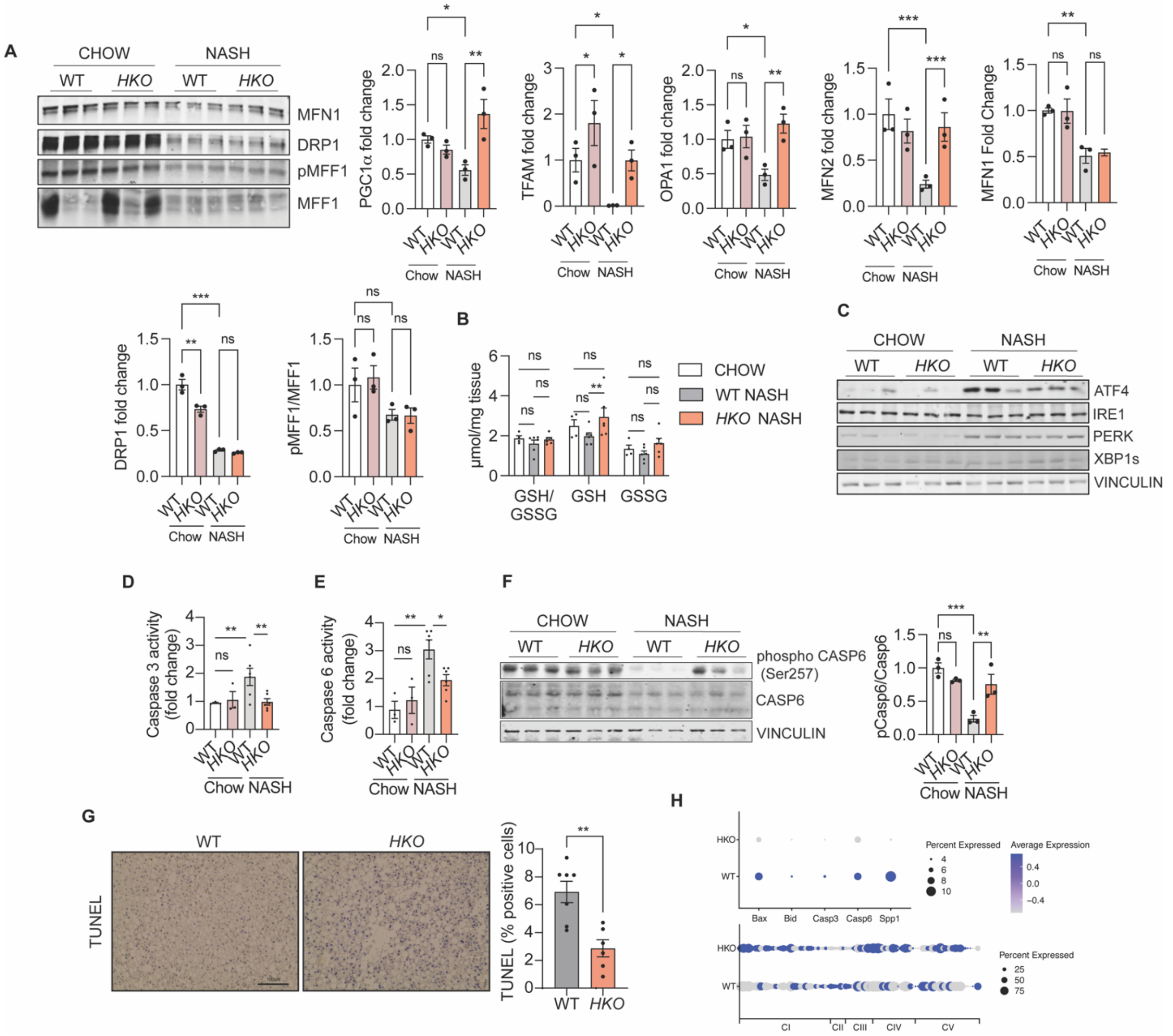

Supplemental Figure 6. Analysis of mitochondrial content, ER stress and cell death in WT and *HKO* livers. (A) Western blot densitometric analysis of MFN1, DRP1 and pMFF1/MFF1 and western blots from Figure 8. **(B)** GSH and GSGG levels and ratio in livers of WT and hepatocyte specific miR-33 knockout (*HKO*). **(C)** Western blot and densitometric analysis of ATF4, IRE1, PERK, XBP1s, and housekeeping VINCULIN in NASH livers from WT and HKO mice. **(D, E)** Caspase 3 and Caspase 6 activity assay in WT and *HKO* liver lysates from mice fed a chow or CD-HFD. **(F)** Western blot and densitometric analysis of phospho-CASPASE6 (Ser257), CASPASE6 and housekeeping VINCULIN**. (G)** Representative images and quantification of TUNEL-stained NASH livers. **(H)** dot plot. Data represent the mean ± SEM. Data represent the mean ± SEM. *P ≤ 0.05, **P ≤ 0.01, ***P ≤ 0.001 compared with WT animals, comparisons and 2-way ANOVA followed by multiple comparison.

A

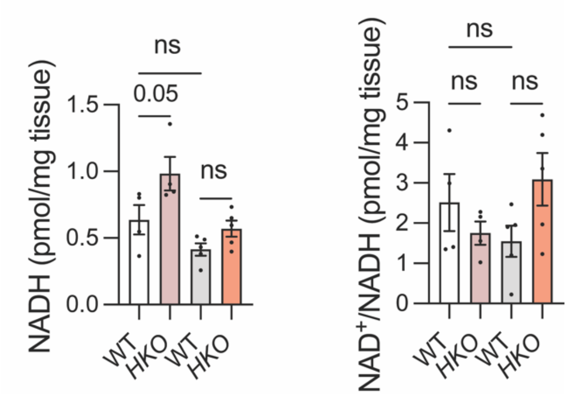

Supplemental Figure 7. NADH/NAD$^+$ regulation in *HKO* livers. (A) NADH and NAD$^+$ levels in WT and hepatocyte specific miR-33 knockout (*HKO*) livers at 6 months of CD-HFD feeding. Data represent the mean ± SEM. *P ≤ 0.05 compared with WT animals, comparisons and 2-way ANOVA followed by multiple comparison.

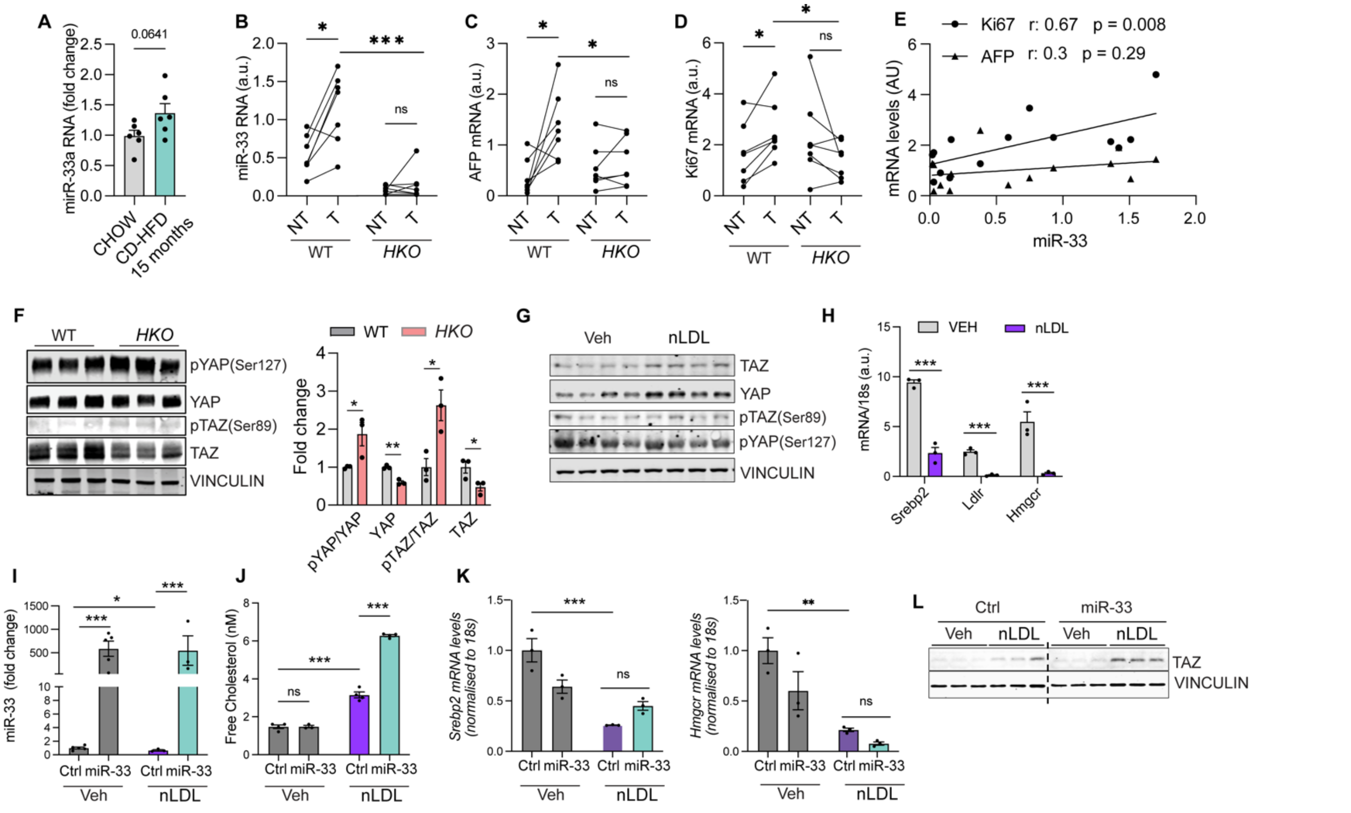

Supplemental Figure 8. miR-33 regulation in liver mouse tumors and effect of miR-33 and cholesterol on YAP/TAZ signaling. (A) miR-33 expression levels in livers from WT mice fed a chow or CD-HFD for 15 months. **(B)** miR-33 expression levels in Tumor (T) and Non-Tumor (NT) samples from WT and *HKO* mice fed the CD-HFD for 15 months. **(C)** AFP expression levels in Tumor (T) and Non-Tumor (NT) samples from WT and *HKO* mice fed the CD-HFD for 15 months. **(D)** Ki67 expression levels in Tumor (T) and Non-Tumor (NT) samples from WT and *HKO* mice fed the CD-HFD for 15 months. **(E)** Linear regression of miR-33/AFP and miR-33/Ki67 in T/NT samples. **(F)** Western Blot and densitometric analysis of YAP/TAZ signaling in WT and *HKO* NASH livers. **(G)** Western blot analysis of YAP/TAZ signaling pathways in AML12 cells loaded with LDL (120μg/ml). **(H)** qPCR analysis of cholesterol response genes. **(I)** miR-33 expression measured by qPCR in AML12 cells with or without LDL and transfected with mimic-miR-33a. **(J)** Cholesterol levels in miR-33 overexpressing or miR-Ctrl AML12 cells treated with or without LDL loading. **(K)** qPCR analysis and **(L)** Western blot analysis of total TAZ levels in AML12 cells with or without miR-33 overexpression and loaded with LDL (120μg/ml). *P ≤ 0.05, **P ≤ 0.01, ***P ≤ 0.001 compared with WT animals, comparisons and 2-way ANOVA followed by multiple comparison.

Table 1. List of genes found in the different intersections from the Interactive Veinn analysis.

[miR-33 Targets] and [upregulated chow] and [upregulated NAFLD:	[miR-33 Targets] and [upregulated chow]:	[miR-33 Targets] and [upregulated NAFLD]:	[upregulated chow] and [upregulated NAFLD:
Abca1	Cyp4f13	Ccrl2	Xlr4a
Ski	Asl	Ccdc15	Col13a1
Atp11c	Zfp281	Cd5l	Rps27
Ddx3x	Foxp1	Veph1	Kit
Il17ra	Smarca5	Clasp1	Meis2
Zbtb2	Nfix	Slc25a25	Srgn
	C8b	Zc3h12c	Vopp1
	Cep68	Hipk2	Ctdspl2
	Ttll7	Ttc28	Flt1
	Dusp4	Fam84a	Cd300lg
	Cbx5	Nnt	Zfp143
	Slc25a51	Gls	Junb
	Atp8b1	Zbtb46	Sirt1
	Ppp4r4	Crk	Gm15638
	Kcnq1	Unc79	Srrt
	Oaf	Sntb2	Fcna
	Dmgdh	Metrnl	Chsy3
	Smad7	Rassf2	Bmp2
	Bcl11b	Cep170	B3gnt2
	Larp4b	Ncoa7	Arhgef10l
	Desi1	Celf1	Fiz1
	Ptbp3	Senp5	Txlng
	Nrn1	Tlr4	Notum
	Csrp2	Bend3	Slc6a6
	Zfp697	Plxnc1	Rai14
	Car8	Tab3	Clec4g
	Dmxl2	Clpx	Npr2

	Slc16a10<br>Fubp1<br>C9<br>Tmx4<br>Sp1<br>Prr16<br>Atp11a<br>Hspb8<br>Sfpq<br>Slc1a2<br>C8a<br>Ern1<br>Zbtb43<br>Tfb1m<br>Rgn<br>Cdh1<br>Map2k6<br>Aass<br>Atrnl1<br>Eif4e3<br>Taf7<br>Ccnd2<br>Zfp141<br>Tfdp2<br>Egfr<br>Lin7a<br>Raph1<br>Atp1b1<br>S1pr1<br>Slc30a1<br>Irs2<br>Sec23ip<br>Ormdl1<br>Abhd13	Mlxip<br>Samd4<br>Mapk6<br>Synm<br>Rasgrp3<br>Camsap2<br>Axl<br>Zfp516<br>Slc2a13<br>Qser1<br>Igsf10<br>B3galt2<br>Anxa3<br>Rhod<br>Gm9992<br>Myo9b<br>Atg2b<br>Scrib<br>Zbtb42<br>Bdp1<br>Cep290<br>Dennd1b<br>Enpp6<br>Nbea<br>Zeb2<br>Gm17484<br>Lyz2<br>Dusp1<br>Slmap<br>Pin1rt1<br>Nab1<br>Fabp7<br>Cyyr1<br>Ubap2l	Tspan7<br>Abcb11<br>Tab2<br>Sash1<br>Osbpl9<br>Zfp318<br>Sf1

	Plg<br>Ttc39c<br>Scara5<br>B3galt1<br>Ctnnd1<br>Atxn1<br>Ythdf1<br>Gm4788<br>Zfp595<br>Irf5<br>Gm10033<br>Sf3a1<br>Anxa13<br>Edem1<br>Gpbp1<br>Ttll4<br>Klf15<br>Zfp81<br>Cdc37l1<br>Arhgap29<br>Lurap1l<br>Uap1<br>Hmgn5<br>Serpind1<br>Crem<br>Zfp236<br>Wdfy1<br>Atxn7l3b<br>Camk4<br>Chic1<br>Cav2<br>Cep85l<br>Socs4<br>Tgfbr3	Ccr9<br>Txnrd1<br>Cybrd1<br>Ahrr<br>9930111J21Rik2<br>Dmxl1<br>Mak<br>Zfp429	

	Ireb2<br>Tob2<br>Srgap3<br>Ammecr1l<br>Magi3<br>Cpsf6<br>Ccdc127<br>Cutc<br>Ang<br>Ctdsp2<br>Acadsb<br>St3gal6<br>Ramp2<br>Rnase4<br>Cfhr1<br>Tom1l1<br>Gpcpd1<br>Ppp1r3c<br>Slc12a8<br>Cfh<br>Cfhr2<br>Tk1<br>Prps1l3<br>Ergic2<br>Mdm4<br>Tmem229a<br>Rpp14<br>Pdgfc<br>Pitpnc1<br>Cpne3<br>Lama3<br>Mbd1<br>Smim13<br>Gm11437		

	Prok1<br>Spin1<br>Ccdc117<br>Itih5		
