# Supplementary material for: miR-33 deletion in hepatocytes attenuates MASLD-MASH-HCC progression
Source: JCI Insight. 2024 Aug 27;9(19):e168476. doi: 10.1172/jci.insight.168476 (PMC11466198; doi:10.1172/jci.insight.168476)

BLOTS FROM FIGURE 2K

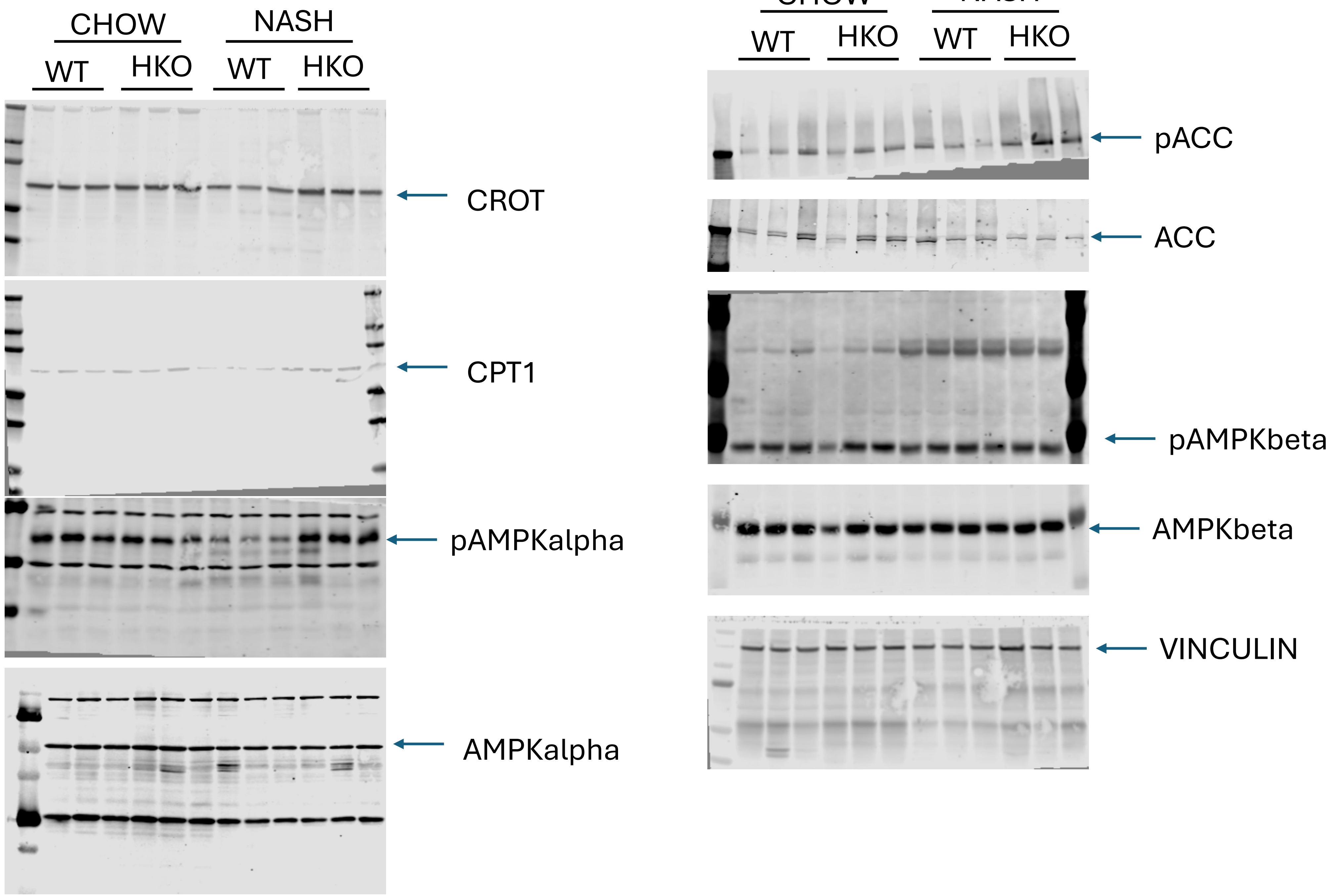

BLOTS FROM FIGURE 4c

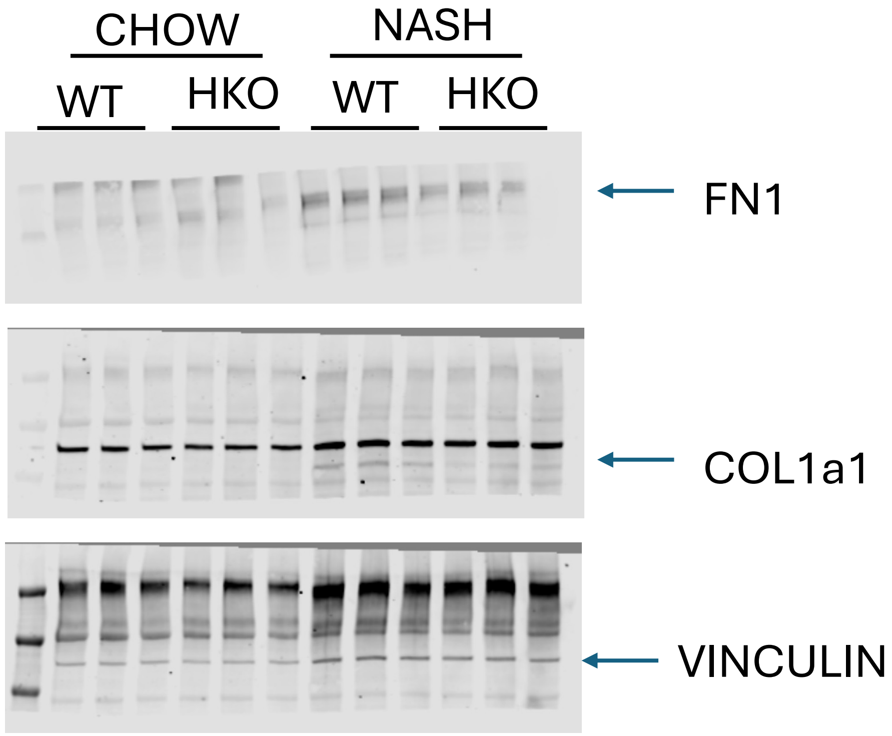

BLOTS FROM FIGURE 6

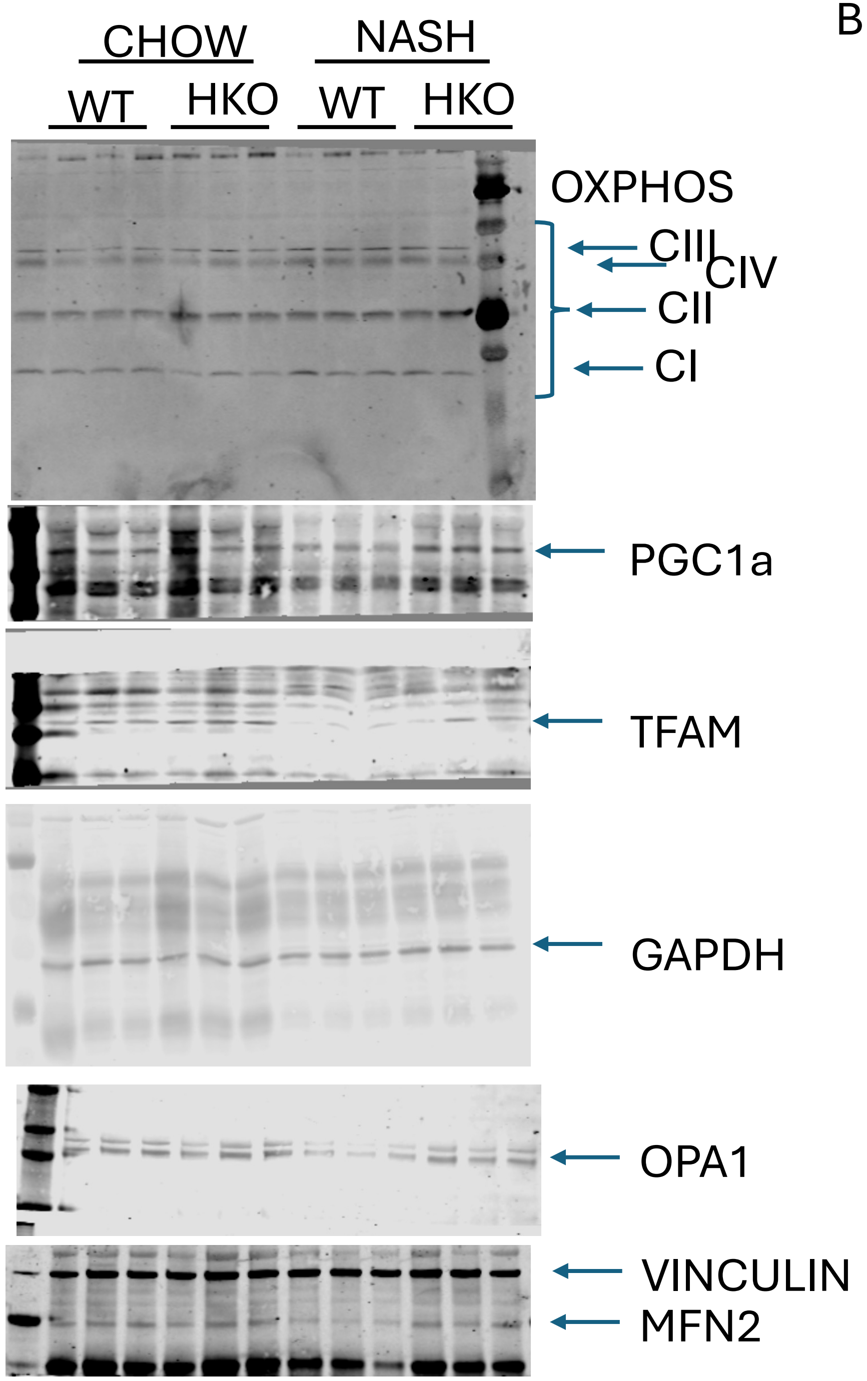

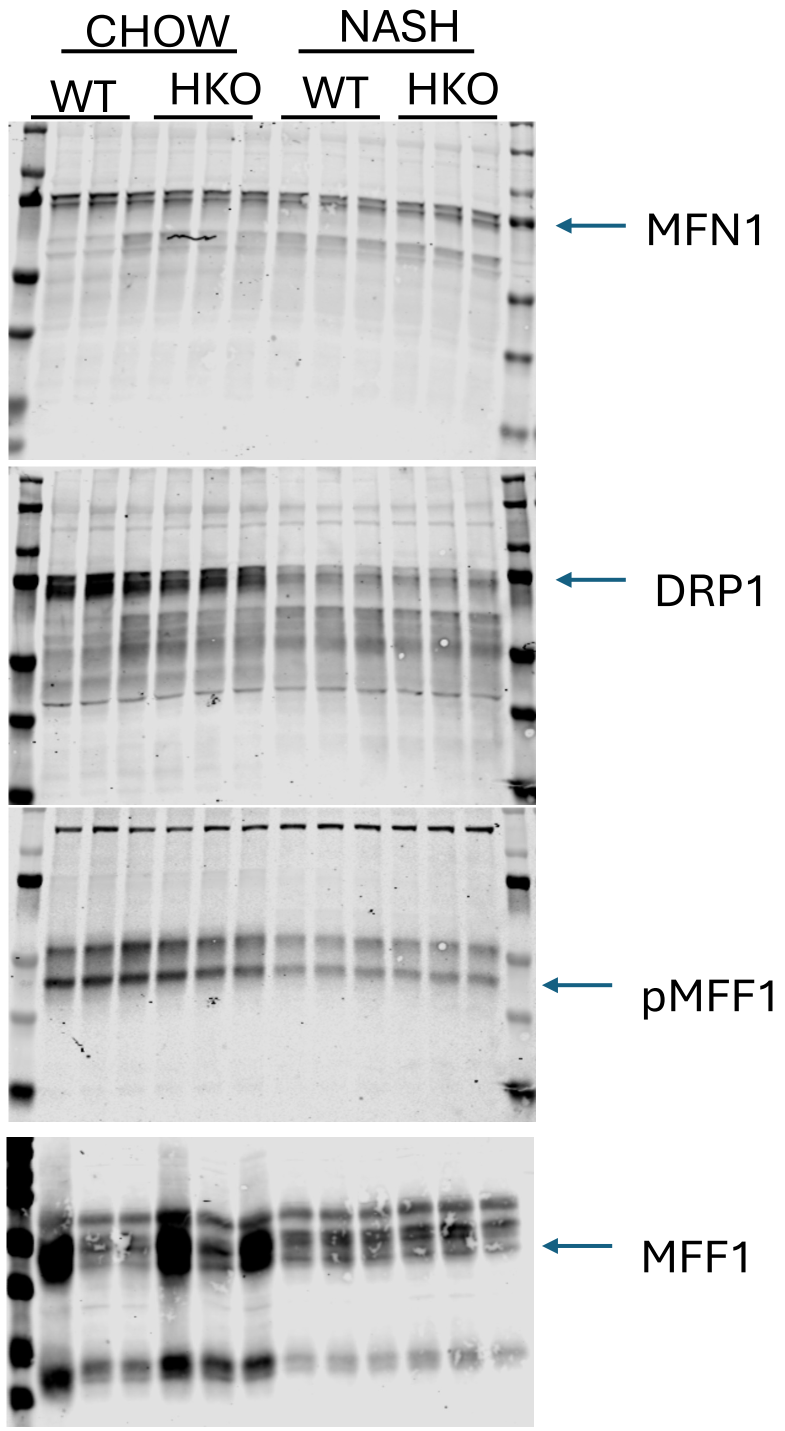

BLOTS FROM FIGURE 7A

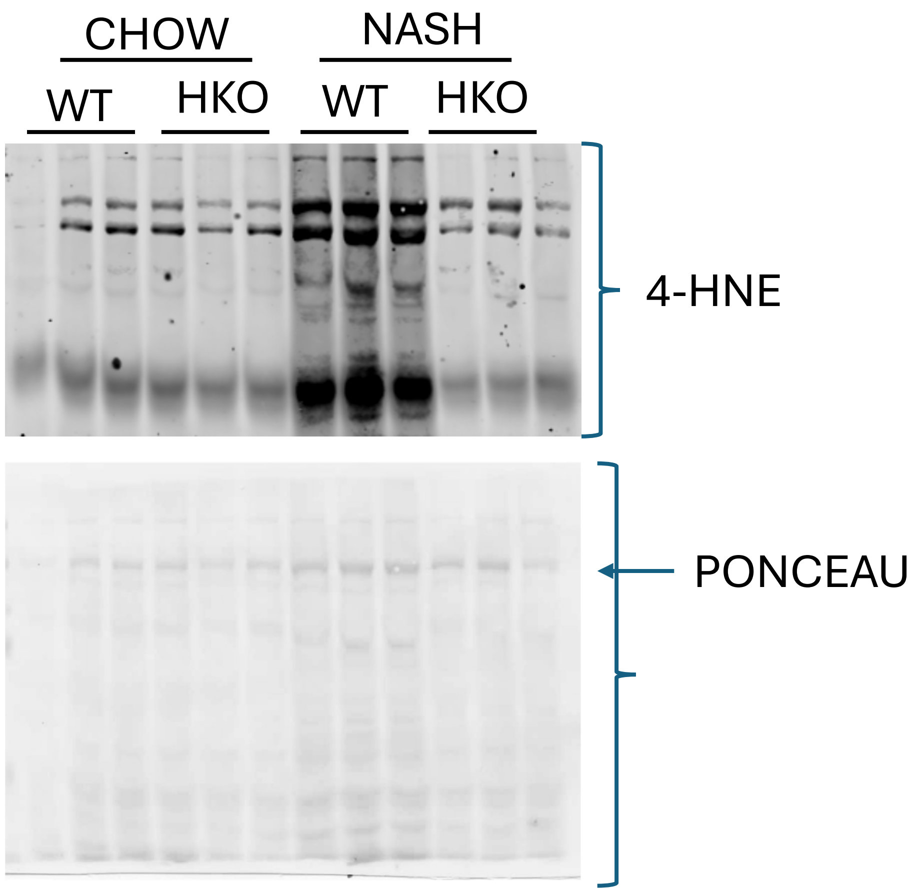

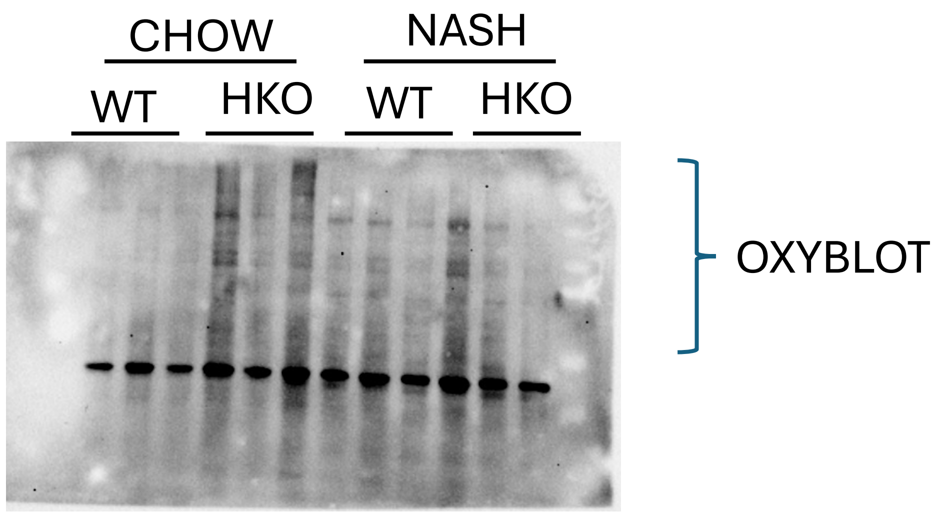

BLOTS FROM FIGURE 8A

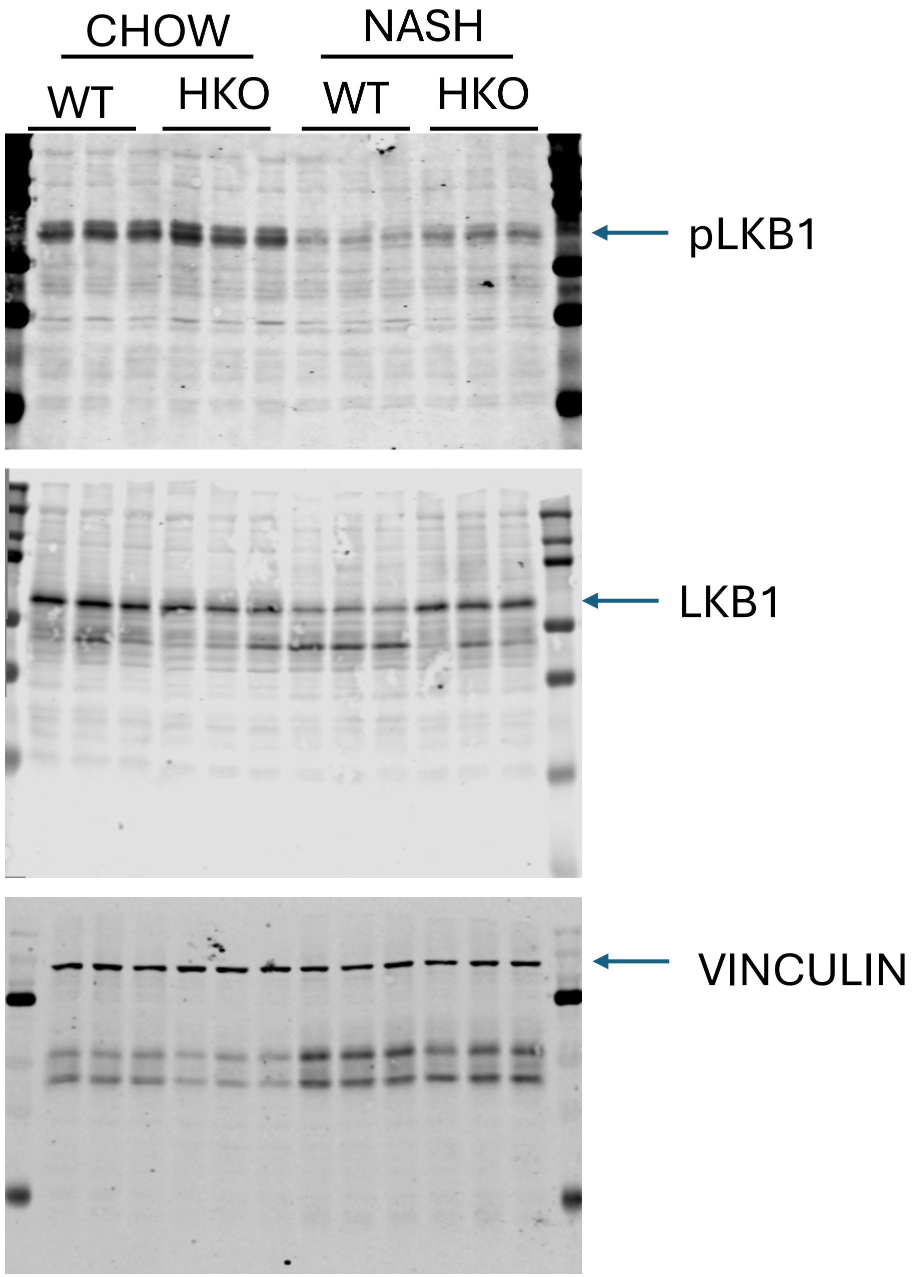

BLOTS FROM FIGURE 8C

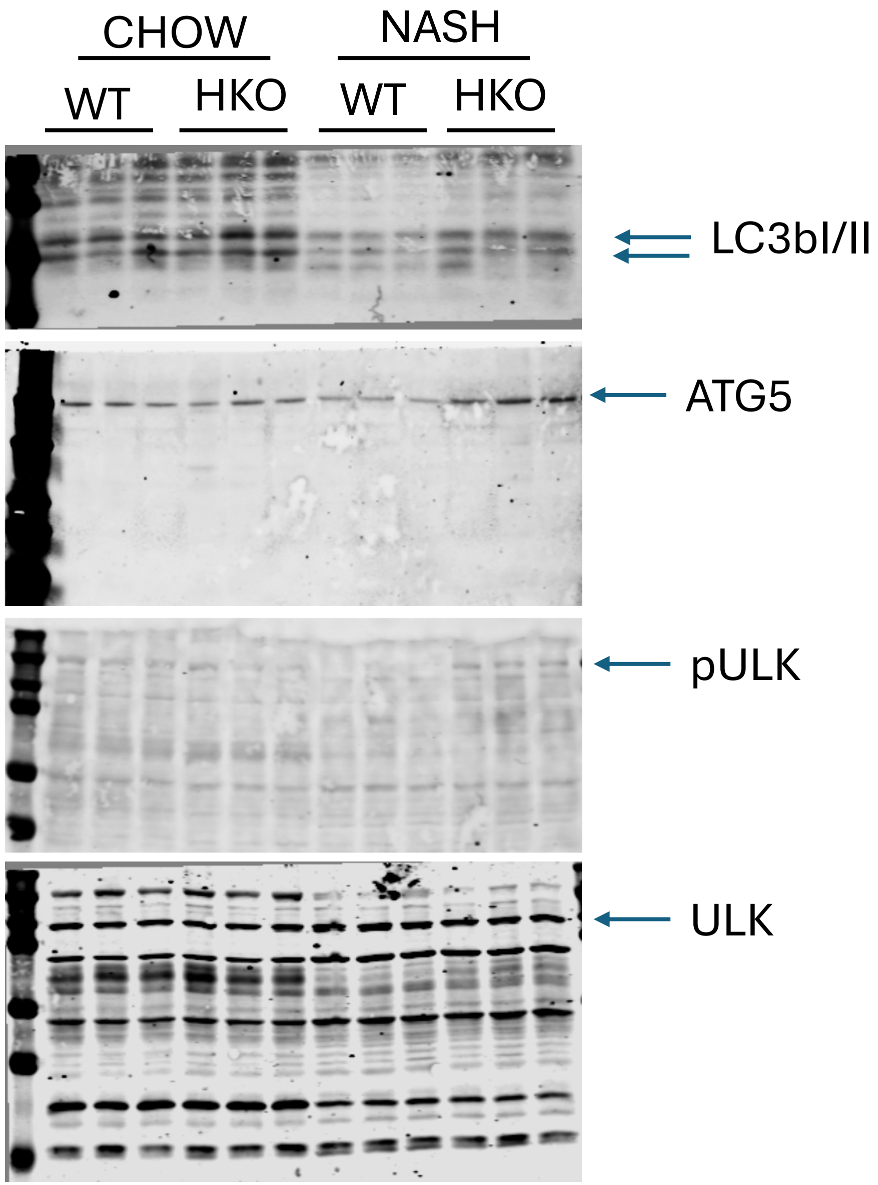

BLOTS FROM FIGURE 8B

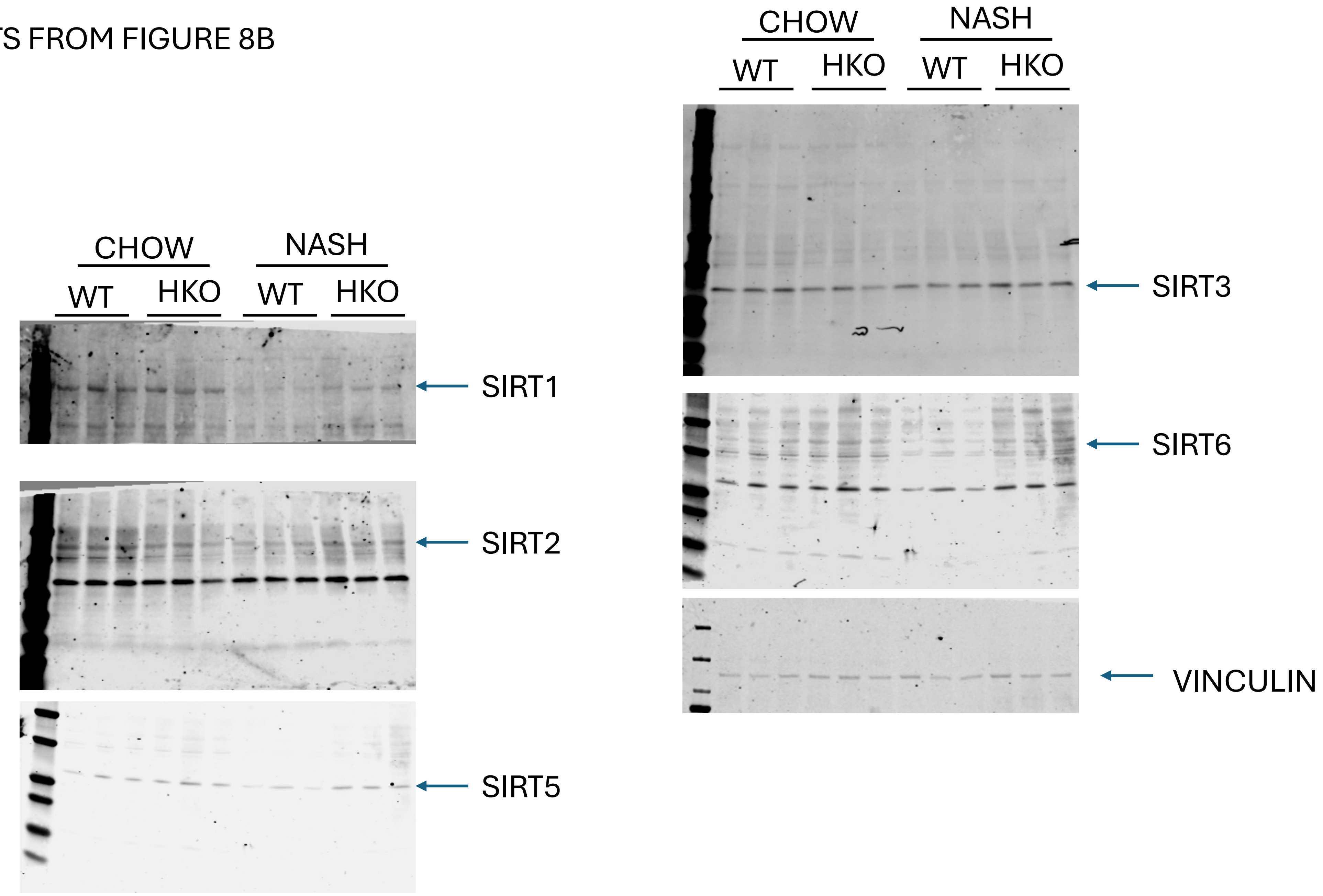

BLOTS FROM FIGURE 9F

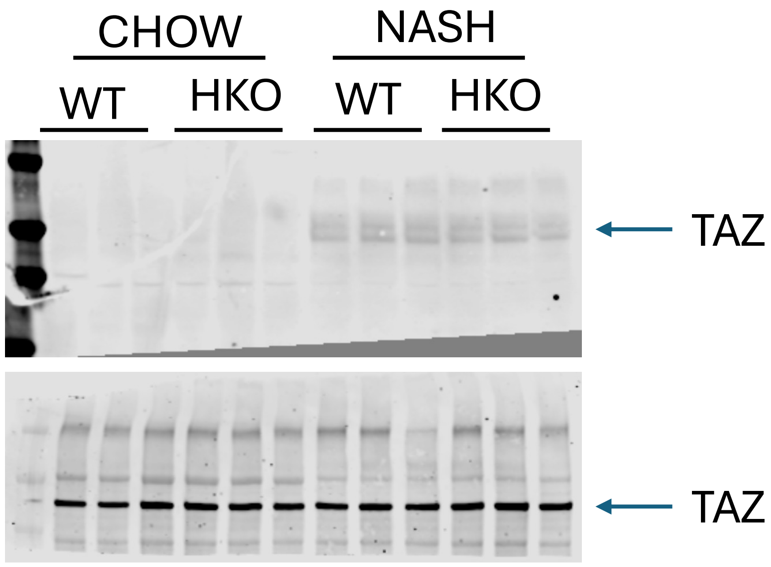

BLOTS FROM FIGURE 9G

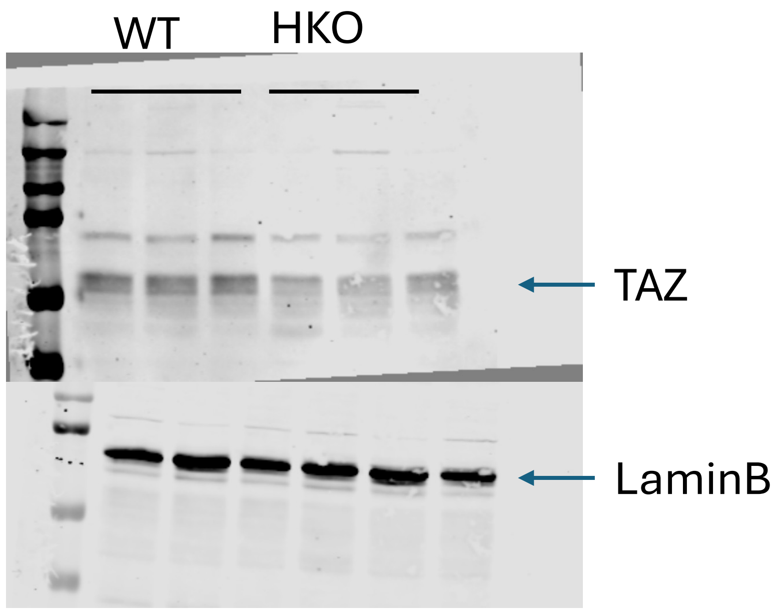

BLOTS FROM SUPPLEMETNAL FIGURE 2

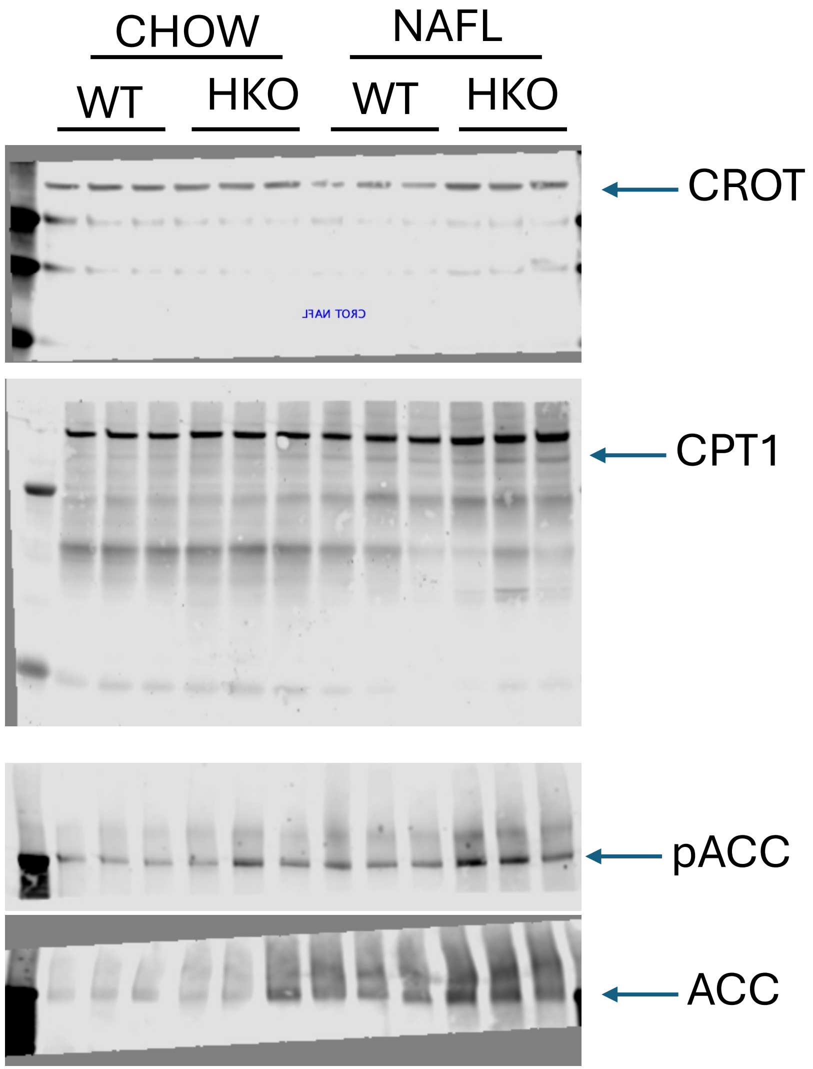

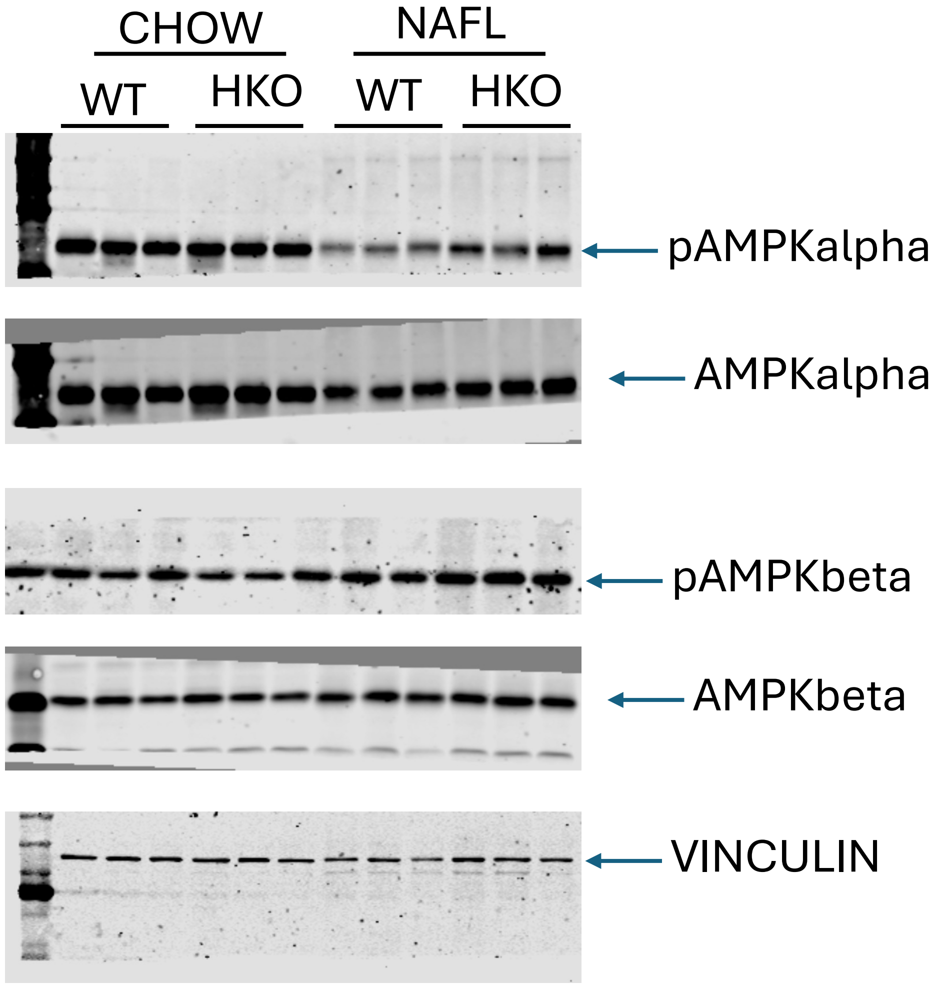

BLOTS FROM SUPPLEMETNAL FIGURE 3A

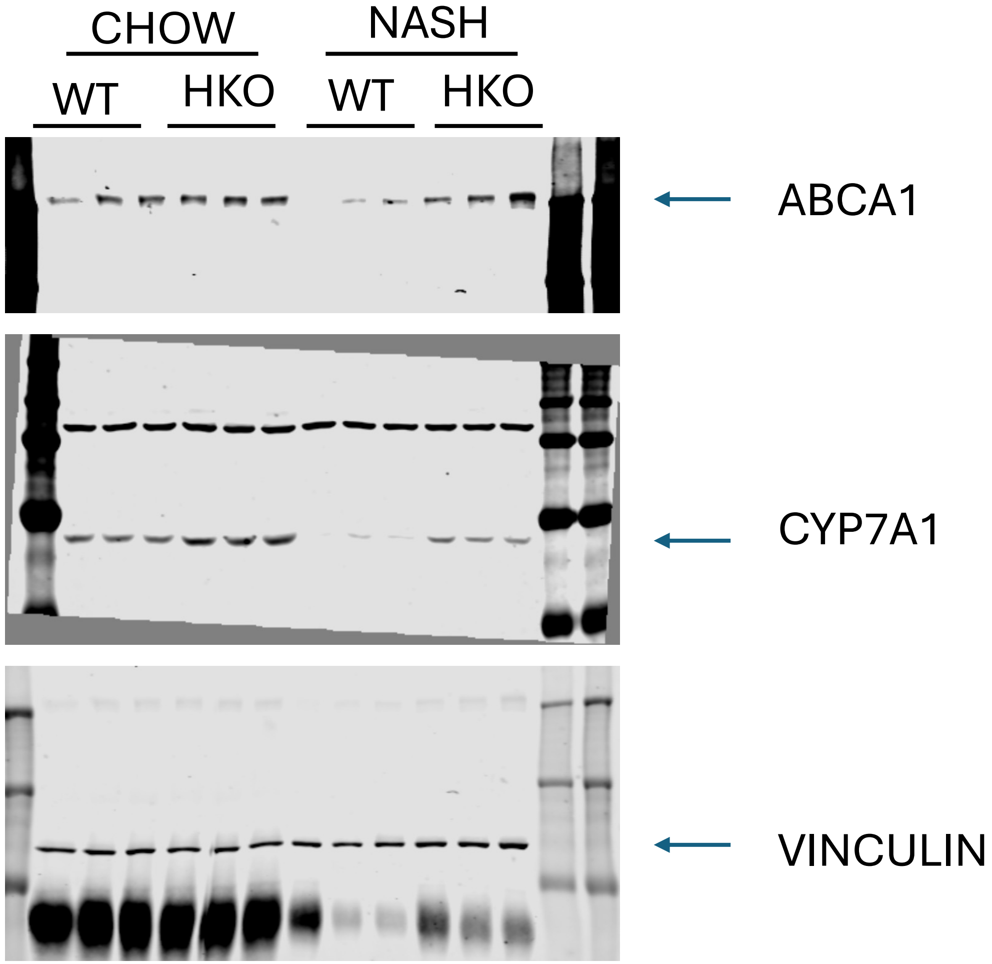

BLOTS FROM SUPPLEMETNAL FIGURE 6C

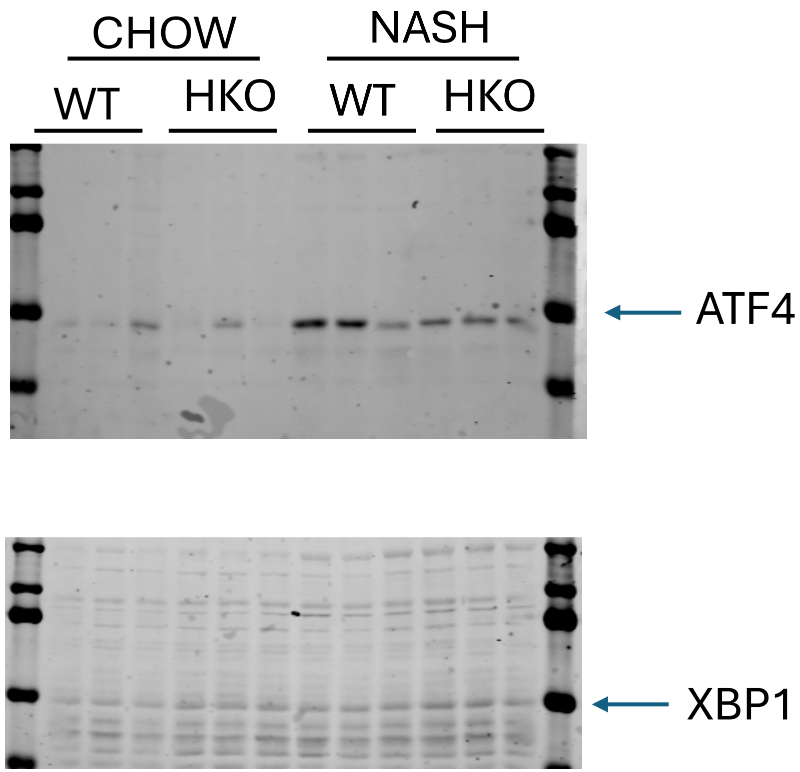

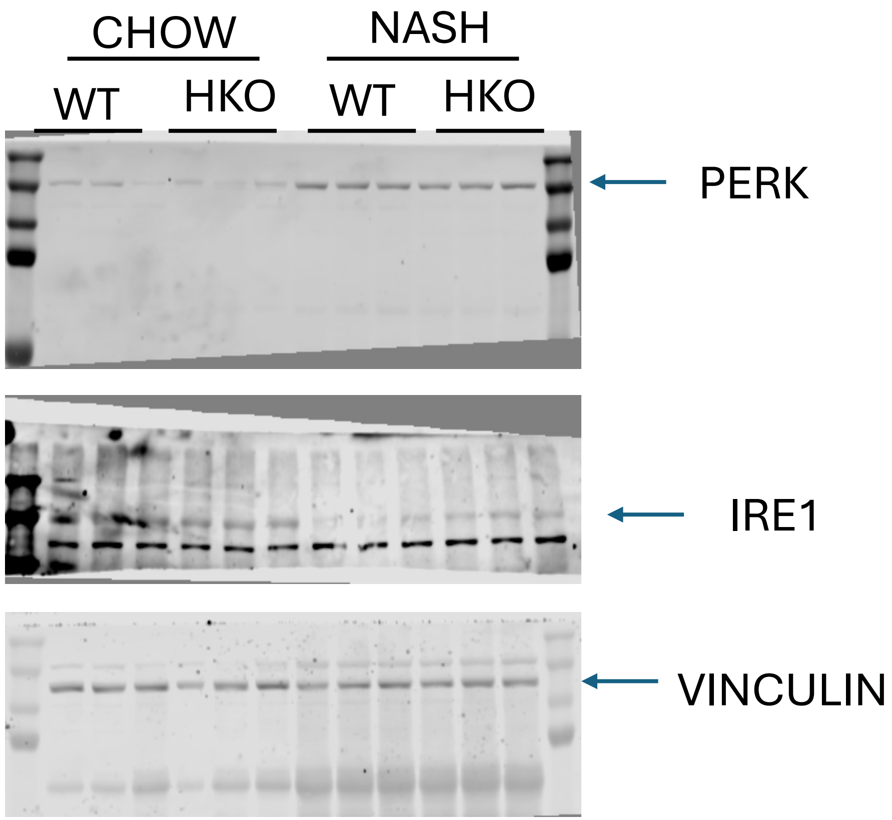

BLOTS FROM SUPPLEMETNAL FIGURE 6F

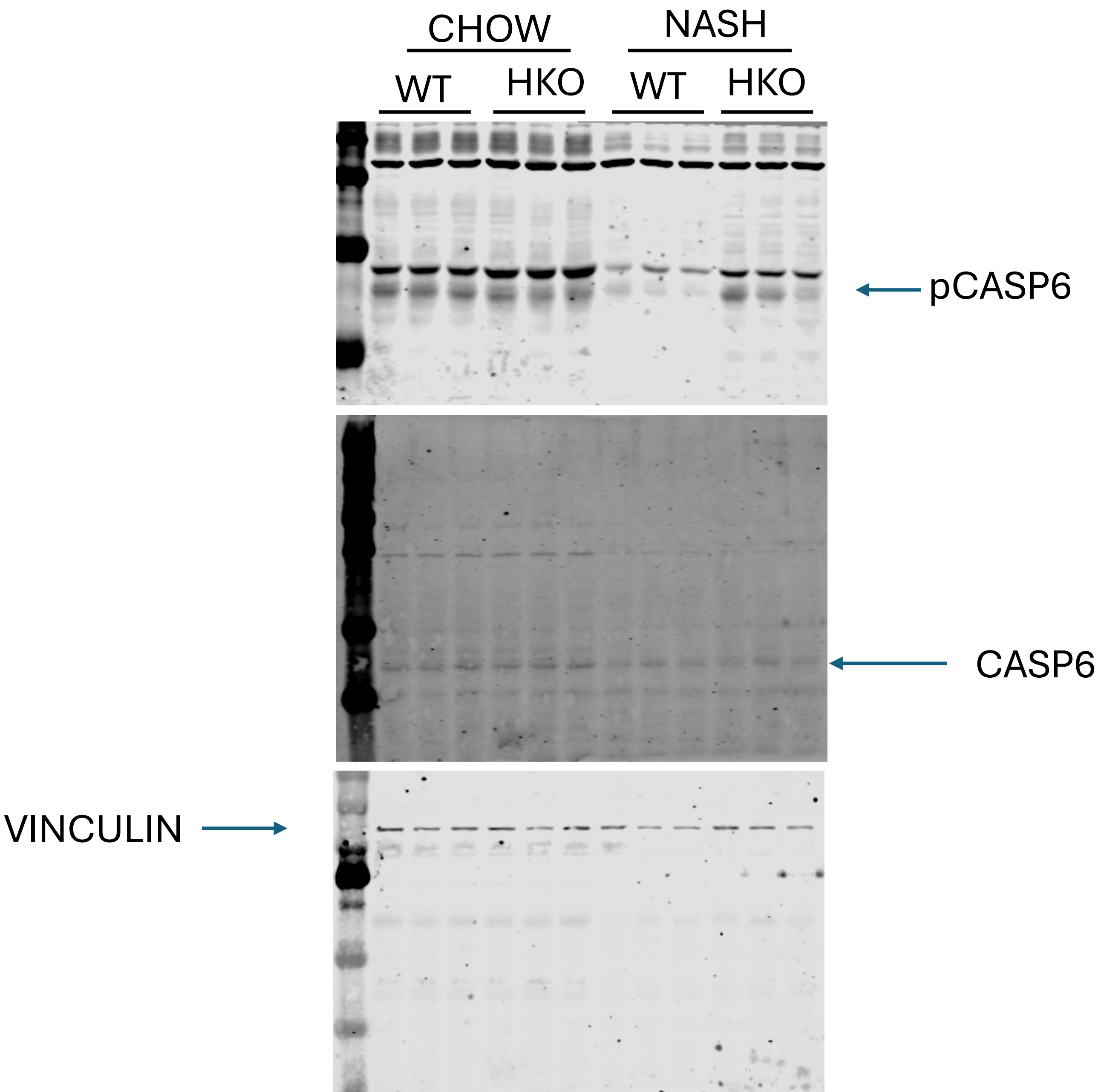

BLOTS FROM SUPPLEMETNAL FIGURE 8F

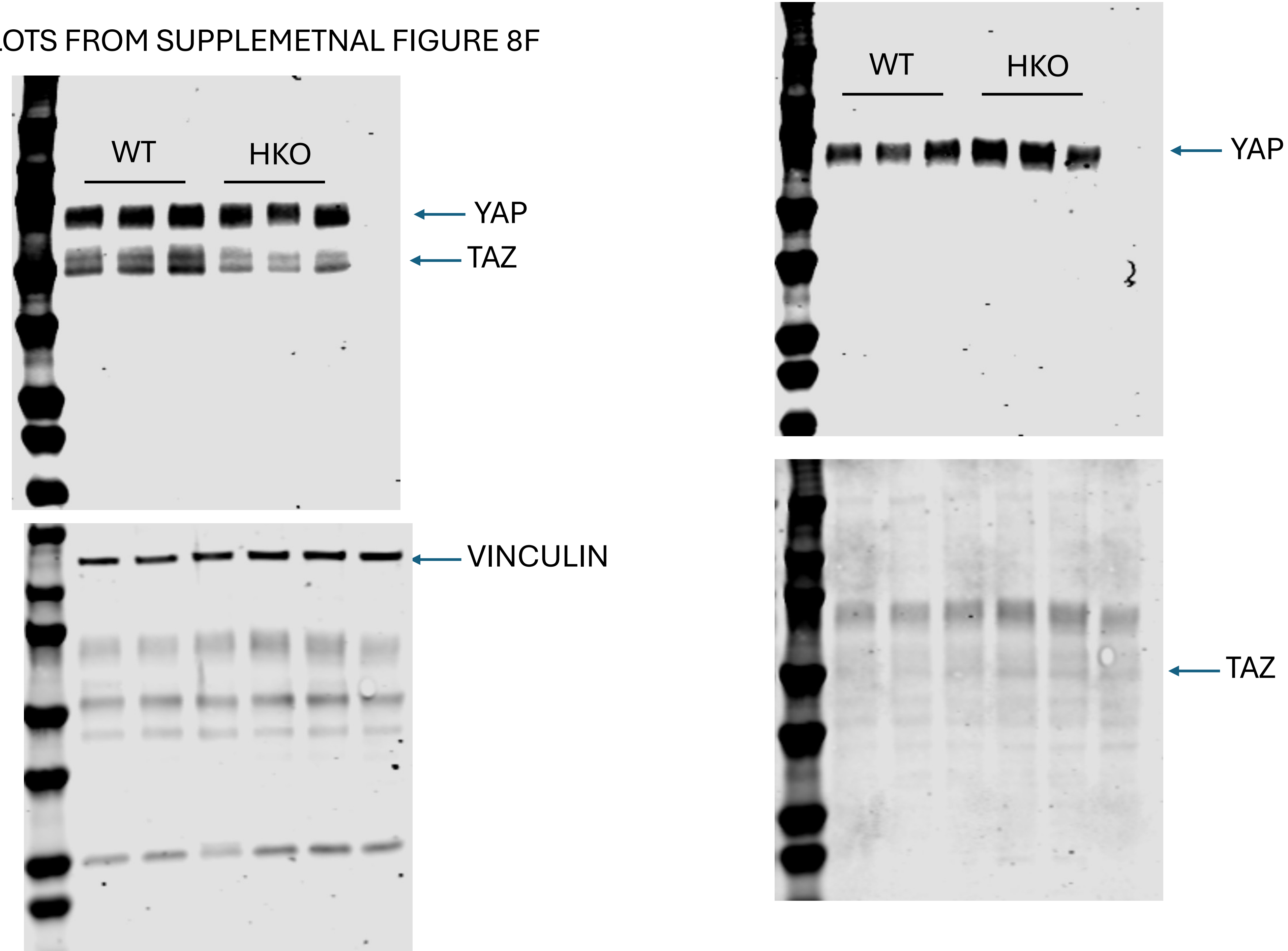

BLOTS FROM SUPPLEMETNAL FIGURE 8G

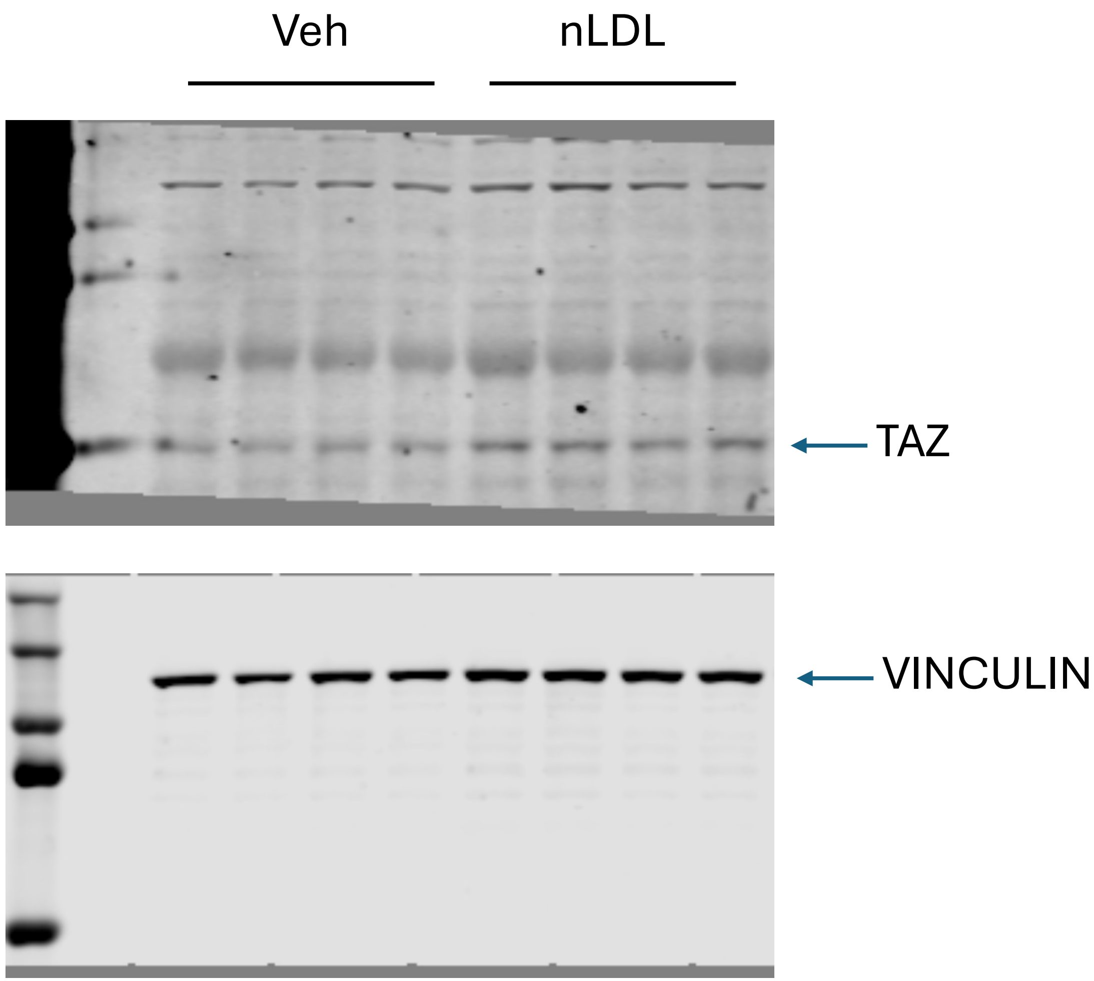

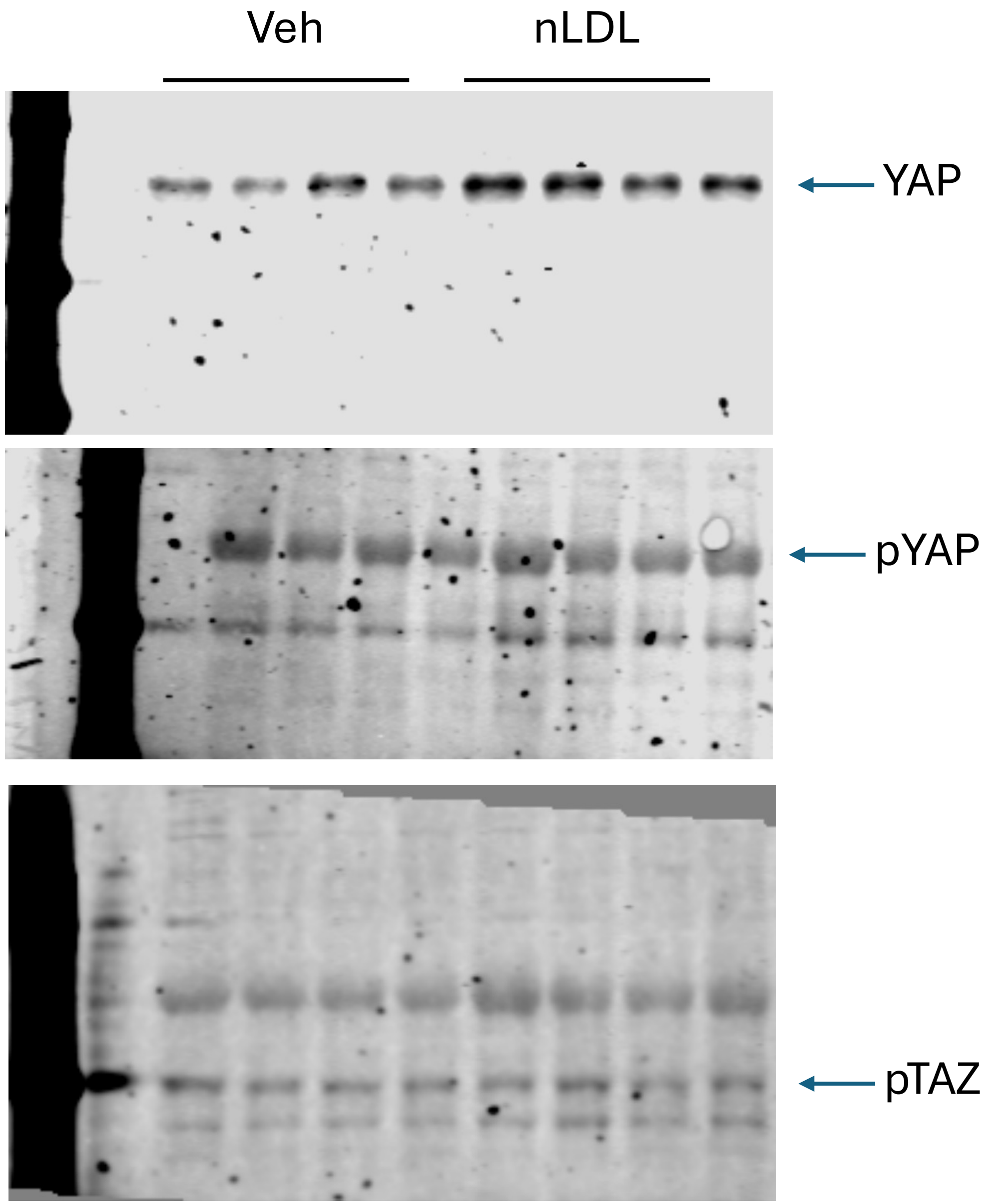

BLOTS FROM SUPPLEMETNAL FIGURE 8L

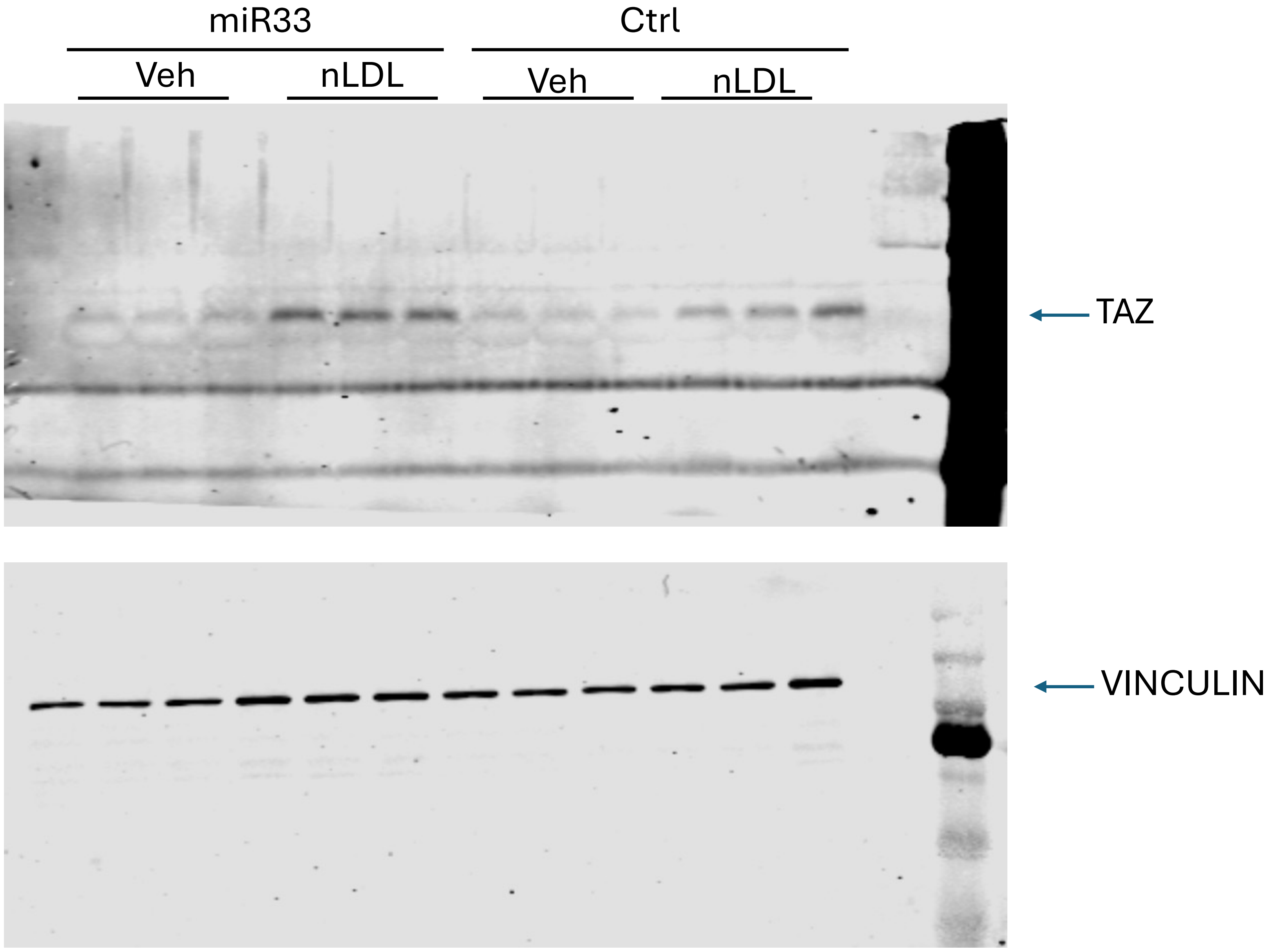

Supplement: Unedited blot and gel images [file jciinsight-9-168476-s187.pdf]
